# Reproductive switching analysis of *Daphnia similoides* between sexual female and parthenogenetic female by transcriptome comparison

**DOI:** 10.1038/srep34241

**Published:** 2016-09-27

**Authors:** Ya-Nan Zhang, Xiu-Yun Zhu, Wen-Ping Wang, Yi Wang, Lu Wang, Xiao-Xue Xu, Kun Zhang, Dao-Gui Deng

**Affiliations:** 1 College of Life Sciences, Huaibei Normal University, Huaibei, China

## Abstract

The water flea *Daphnia* are planktonic crustaceans commonly found in freshwater environment that can switch their reproduction mode from parthenogenesis to sexual reproduction to adapt to the external environment. As such, *Daphnia* are great model organisms to study the mechanism of reproductive switching, the underlying mechanism of reproduction and development in cladocerans and other animals. However, little is known about the *Daphnia*’s reproductive behaviour at a molecular level. We constructed a genetic database of the genes expressed in a sexual female (SF) and a parthenogenetic female (PF) of *D. similoides* using Illumina HiSeq 2500. A total of 1,763 differentially expressed genes (865 up- and 898 down-regulated) were detected in SF. Of the top 30 up-regulated SF unigenes, the top 4 unigenes belonged to the Chitin_bind_4 family. In contrast, of the top down-regulated SF unigenes, the top 3 unigenes belonged to the Vitellogenin_N family. This is the first study to indicate genes that may have a crucial role in reproductive switching of *D. similoides*, which could be used as candidate genes for further functional studies. Thus, this study provides a rich resource for investigation and elucidation of reproductive switching in *D. similoides*.

The water flea *Daphnia* are planktonic crustaceans commonly found in freshwater environments. They are sensitive to environmental changes and are a primary food source for fish and other invertebrate predators, thus playing a key role in aquatic ecosystems^1^,^2^,^3^.

As a response to environmental stimuli, *Daphnia* can switch their reproduction mode from parthenogenesis to sexual reproduction. This unique reproductive strategy includes two stages: they produce clonal female offspring by parthenogenesis under normal conditions; and in response to certain unfavourable environmental and biological factors, such as shortened daylight, low temperature, overpopulation, and lack of nutrients, they can switch to sexual reproduction and produce male offspring^4^,^5^,^6^,^7^,^8^,^9^. Barton’s studies showed that sexual reproduction leads to an increase in genetic diversity and survival rate, while parthenogenesis contributes to rapid propagation during favourable seasons^10^. These findings indicate that both different reproductive tissues (for ovarian development and breeding of offspring) and chemosensory tissues (for chemosensation of environmental changes such as temperate, food, population density, and so on) may be involved in reproductive switching of *Daphnia*. Hence, *Daphnia* is a great model organism for studying the mechanism of reproductive switching in cladocerans and other animals. However, very little is known about the molecular processes involved in *Daphnia*’s reproductive behaviour.

To thoroughly explore the mechanisms of reproductive switching, molecular identification, differential expression analysis in sexual females (SF) and parthenogenetic females (PF), as well as functional analyses of genes related to reproductive switching are the primary steps that should be performed. RNA-seq is considered to be a timesaving, cost effective, and highly efficient method, which has been used lately in large-scale studies identifying target genes in *Daphnia* and other invertebrates whose genomes have not been sequenced^11^,^12^,^13^,^14^,^15^.

The aim of the present study was to construct a genetic database of genes expressed in SF and PF of *D. similoides* and thus provide a rich resource of data for investigation and elucidation of reproductive switching in *D. similoides*. We further identified reproductive switching-related genes by comparing the transcriptome sequencing data and, as the first step toward understanding the physiological processes involved in reproductive switching, conducted a comparative analysis of differentially expressed genes (DEGs) and identified a set of up-regulated and down-regulated genes in SF and PF.

## Results

### Transcriptome sequencing and sequence assembly

**Figure 1:**
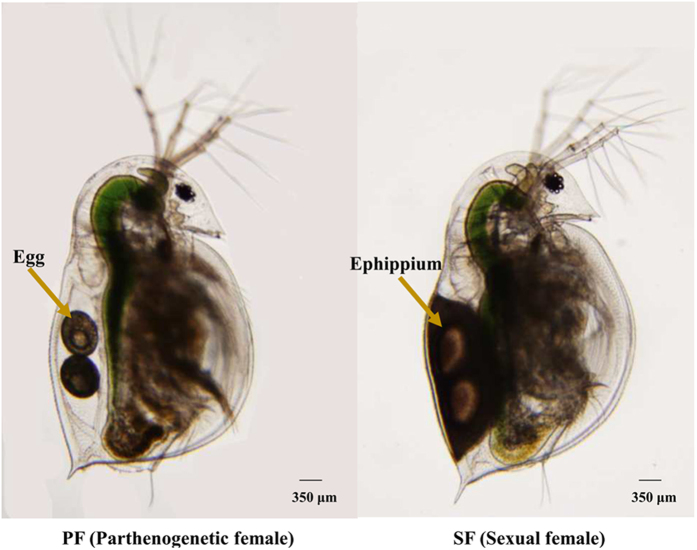
The photograph of *D. similoides* female.

**Figure 2:**
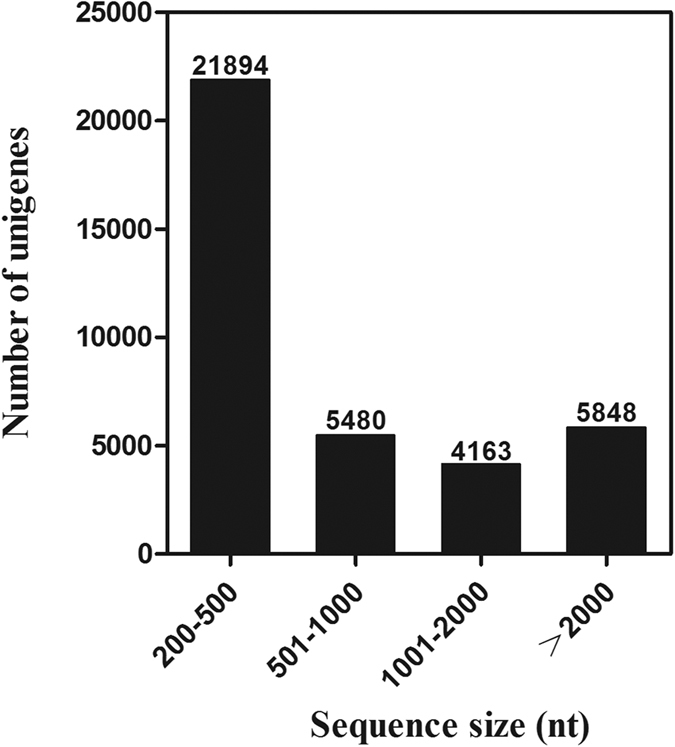
Distribution of unigene size in the *D. similoides* transcriptome assembly.

We carried out a next-generation sequencing project on a cDNA library constructed from *D. similoides* (Fig. 1) using an Illumina HiSeq™ 2500 platform. Low quality raw reads and reads with adapters and N content of more than 10% were excluded. The number of clean reads obtained from SF and PF of *D. similoides* was 62,395,405 and 52,037,554, respectively. All clean reads were assembled into transcripts by Trinity software; the longest copy of redundant transcripts was regarded a unigene^16^,^17^. In total, 61,047 transcripts were obtained and assembled into 37,385 unigenes. Of the 37,385 unigenes, those with a sequence length more than 500 bp accounted for 41.43% of the transcriptome assembly (Fig. 2). All the clean reads of SF and PF are deposited and available from the NCBI/SRA data base (SRA experiment accession number: PF: SRX1645097, SF: SRX1645182).

### Homology analysis and gene ontology annotation

**Figure 3:**
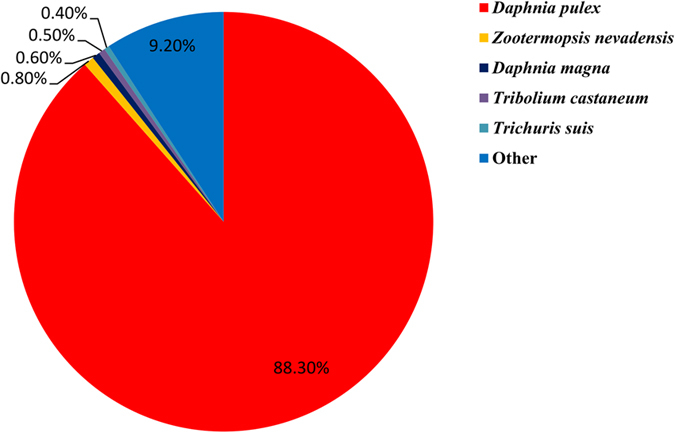
Percentage of homologous hits of the *D. similoides* transcripts to other species. The *D. similoides* transcripts were searched by Blastx against the non-redundancy protein database with a cutoff E-value 10^−5^.

Among the 37,385 unigenes, 13,072 were matched by the BLASTX homology search to the entries in NCBI non-redundant (Nr) protein database with a cut-off E-value of 10^−5^. The highest percentage of matching bases (88.30%) was to the *D. pulex* sequences, followed by the sequences of *Zootermopsis nevadensis* (0.80%), *D. magna* (0.60%), *Tribolium castaneum* (0.50%), and *Trichuris suis* (0.40%) (Fig. 3).

The gene ontology (GO) annotation was used to classify the transcripts into functional groups according to the GO category. Of 37,385 unigenes, 12,113 (32.40%) were annotated based on sequence homology. In the molecular function category, the genes expressed in SF and PF were mostly enriched to binding, catalytic activity, and transporter activity. In the biological process category, the cellular, metabolic, and single-organism processes were the most represented. In the cellular component category, the cell, cell part, and organelle were the most abundant (Supplementary Fig. S1).

### Differentially expressed genes

**Figure 4:**
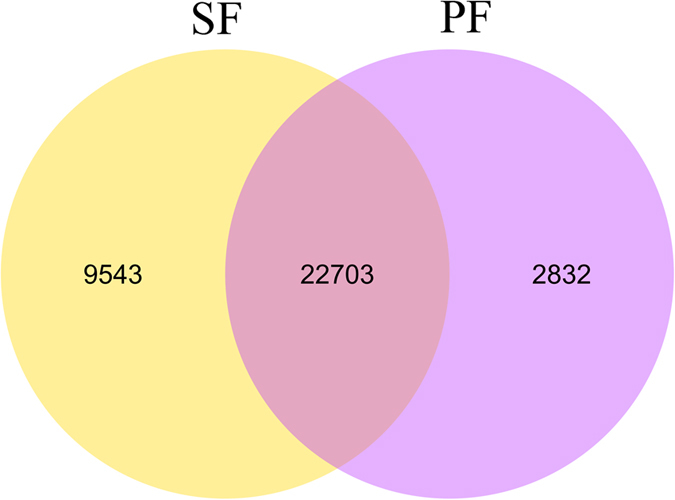
Venn diagram of the number of unigenes with reads per kilo bases per million mapped (RPKM) > 0.3 in SF and PF. SF: sexual female, PF: parthenogenetic female.

**Figure 5:**
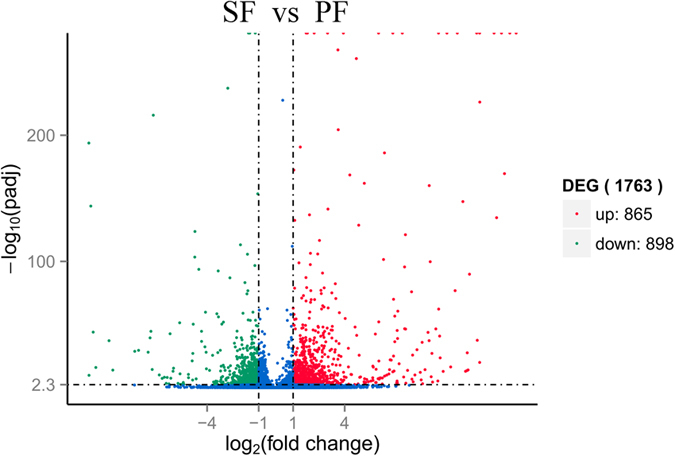
Volcano plot of differentially expressed genes in SF and PF. Differentially expressed genes were selected by q-value < 0.005&|log2 (fold change)|> 1 according the method of Storey *et al*.^18^. The x-axis shows the fold change in gene expression between SF and PF, and the y-axis shows the statistical significance of the differences. Splashes represent different genes. Blue splashes means genes without significant different expression. Red splashes means significantly up expressed genes. Green splashes means significantly down expressed genes. SF: sexual female, PF: parthenogenetic female, −log10(padj): the corrected p-value.

DEGs were selected by RSEM with conditions of log_2_ fold change > 1 and *q*-value < 0.005^18^. The number of unigenes with RPKM > 0.3 expressed specifically in SF or PF was 9,543 and 2,832, respectively, and 22,703 unigenes were common in both SF and PF (Fig. 4). In the comparative analysis, 865 and 898 unigenes in SF were expressed at significantly higher and lower levels, respectively, compared to those in PF (Fig. 5).

### GO enrichment analysis

**Figure 6:**
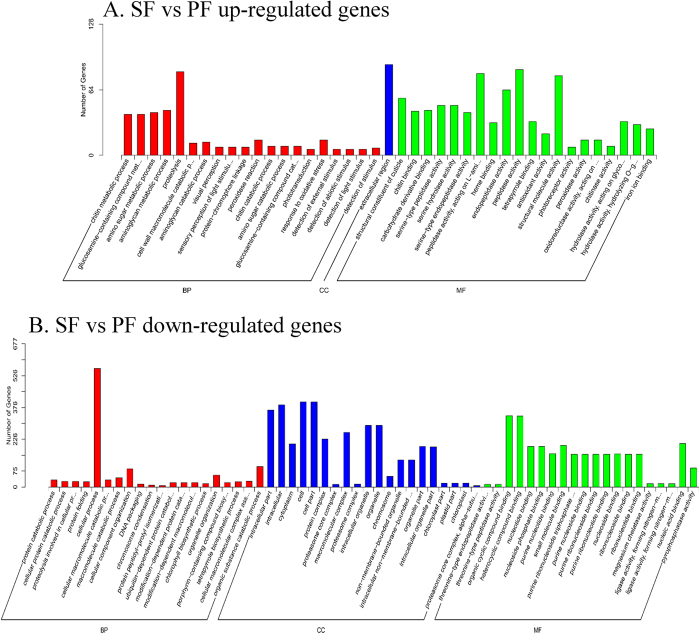
GO enrichment analysis of differentially expressed genes in SF and PF. BP: Biological Process, MF: Molecular Function, CC: Cellular Component, SF: sexual female, PF: parthenogenetic female. (**A**) SF vs PF up-regulated genes; (**B**) SF vs PF down-regulated genes.

To compare the functions of these DEGs in SF and PF, we conducted GO enrichment analysis of the identified 1,763 DEGs with the threshold value for corrected *P* < 0.05 (Fig. 6). The results of the GO enrichment analysis showed that all up-regulated genes (SF vs. PF) were mostly enriched in the extracellular region, peptidase activity, proteolysis, structural molecule activity, and endopeptidase activity GO processes. However, all down-regulated (SF vs. PF) genes were mostly enriched in the cellular process, cell, cell part, intracellular part, and organic cyclic compound binding GO processes.

### Reproductive switching-related genes

To better understand the mechanism of reproductive switching in *D. similoides*, we compared the SF and PF transcriptomes and identified 30 of the most differentially (based on the *q*-value) up-regulated and down-regulated unigenes from SF (Table 1 and Supplementary Table S1 available). Of the top 30 up-regulated SF unigenes, 4 unigenes (gene order: 1, 3, 4, and 14) belonged to the Chitin_bind_4 family, 10 were unknown functional unigenes, and the rest of the unigenes might have participated in the defence, digestion, enzyme metabolism, redox reactions, and transmission of nerve signals. In contrast, of the top down-regulated SF unigenes, 3 unigenes (gene order 1, 2, and 3) belonged to the Vitellogenin_N family, 10 were unknown functional unigenes, and the rest of the unigenes might have been involved in cell growth, differentiation, defence, and regulation of signals.

### Validation of transcriptome data by qPCR

**Figure 7:**
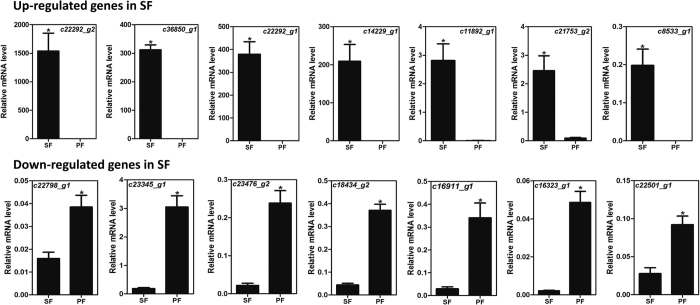
qPCR results of differentially expressed genes in SF and PF. SF: sexual female, PF: parthenogenetic female. The relative expression level is indicated as mean ± SE (N = 3). “*” Means significant difference between SF and PF (*P* < 0.05, Student t-test).

In order to validate the transcriptome results, we randomly selected 14 significant DEGs from Table 1 for quantitative real-time PCR (qPCR) conformation. The primers used for qPCR are shown in Supplementary Table S2 available. The results of the qPCR were consistent with the RNA-seq data (Fig. 7).

## Discussion

*Daphnia* undergo parthenogenesis in suitable environments, forming a large population, while they enter sexual reproduction under unfavourable conditions, producing fertilised eggs^2^,^10^,^19^. However, studies that explore reproductive switching in *Daphnia* at molecular level are scarce or non-comprehensive, so more data was a research priority for investigating the mechanism in this genus. In the present study, we used comparative transcriptome analysis to investigate the differences in gene expression of *D. similoides* and identify those that are involved in reproductive switching.

We carried out the transcriptome *de novo* assembly with short reads because of the lack of *D. similoides* genome sequences. In this study, the N50 of the unigenes were 2,685 bp long, much longer than those reported in other studies^20^,^21^, suggesting high quality sequencing and assembly. Among the 37,385 unigenes identified, only 34.96% gene translations shared significant similarity with entries in the NCBI Nr protein database, indicating that large numbers of the unigenes were either non-coding or specific to *D. similoides*. Additionally, we found that *D. similoides* shared highest similarity with *D. pulex*, 88.30% of sequence similarity, indicating a relatively close phylogenetic relationship between these two species of *Daphnia*.

By comparing the differences in gene expressions in SF and PF of *D. similoides*, we found that the number of genes expressed specifically in PF was greater than that in SF, suggesting that there is a certain correlation between the development of egg chambers as well as embryos and these genes in PF^22^. Remarkably, up-regulated and down-regulated genes of *D. similoides* (SF vs. PF) had different GO enrichment: extracellular region and peptidase activity were the most frequently enriched groups of up-regulated genes. The extracellular region refers to the space external to the outermost structure of the cell; it does not include gene products uniformly attached to the cell membrane. Since the reproduction in SF is sexual under unfavourable environmental and biological conditions^19^,^23^,^24^, the SF receives more stimuli from the external environments, such as perception of the temperature and interaction with the sperm, and resist better the unfavourable conditions compared to PF. Regarding the peptidase activity, some peptidase genes in *D. pulex* might have specific adaptations to the lifestyle of a planktonic filter feeder in a highly variable aquatic environment^25^. In addition, some studies in other animals showed that peptidases play key roles in reproduction; for example, peptidase 2-like gene (*Immp2l*) can impair fertility in mice^26^, and DmCatD is involved in the process of follicular atresia of *Dipetalogaster maxima*^27^, which suggests that certain peptidases in SF of *D. similoides* may have similar functions in the process of adaption to external environment and reproduction. In contrast, cellular and intracellular processes were the most frequently enriched groups of down-regulated genes. The development of an embryo from a female gamete without any contribution by a male gamete indicates that some genes involved in cellular and/or intracellular process (e.g., mutation, hybridisation, microbial infection, and genetic contagion) may be responsible for the transition from SF to PF^28^,^29^,^30^ and the development of reproductive organs^22^,^31^. Additionally, previous studies based on molecular biology showed that embryo-associated^22^ and meiosis-suppression genes^24^,^32^ play key roles in PF of *Daphnia*, and they belong to cellular and/or intracellular processes. Herein, we acknowledge the challenging nature of interpreting the SF/PF up- regulated GO terms. Nonetheless, these GO terms can be broadly associated with morphological plasticity of *D. similoides* and may participate in the reproductive switching between SF and PF.

The PFAM annotation of up-regulated and down-regulated unigenes of SF vs. PF identified two gene families, the Chitin_bind_4 and Vitellogenin_N families, that were more differentially expressed compared to other genes in SF and PF, respectively. The Chitin_bind_gene family is associated with the cuticle in cladoceran^33^ and other invertebrates^34^,^35^. The cladoceran cuticle consists of proteins and chitin and can withstand adverse conditions of the external environment^33^,^36^,^37^. Therefore, the up-regulated expression of cuticle-related genes in SF may help *D. similoides* undergo a series of corresponding changes in cuticle structure as a response to adverse external environment conditions. In contrast, the genes belonging to Vitellogenin_N family play a key role in the formation of yolk proteins in cladoceran crustaceans^38^,^39^,^40^ and help to ensure normal development of the embryo. Previous results^41^ and the results presented herein indicate that some genes from the up-regulated Vitellogenin_N family in PF participate in the process of ovarian development of *D. similoides*. Other DEGs in SF vs. PF may have different functions in sexual reproduction and parthenogenesis, which warrant further studies along with integrated functional studies.

## Conclusions

In conclusion, we identified genes related to reproductive switching by comparing transcriptome sequencing data from SF and PF of *D. similoides*. Our findings suggest that some DEGs are similar to those reported for other *Daphnia* species, which in turn indicates that functional requirements in *Daphnia* are conserved. Thus, these DEGs could be used to study the molecular evolution in *Daphnia.* Our results will not only provide indispensable contributions to the studies of reproductive and evolutionary biology in *Daphnia* but they can also be used as a model to study other cladocerans.

## Methods

### Sample preparation

*D. similoides* was originally obtained from the Lake Chaohu in Anhui Province, China. The field studies did not involve endangered or protected species, and no specific permissions were required for these research activities in these locations. Healthy parthenogenetic organisms were identified and cultivated by a monoclonal method in our laboratory. Briefly, under optimal environmental conditions, the eggs produced by the adult female of *D. similoides*, although not fertilised with sperm, developed directly into juveniles. Such adult females were regarded parthenogenetic females. With worsening of the environmental conditions (such as high population density and food deficiency), some eggs produced by the parthenogenetic females of *D. similoides* developed into males, while others developed into females, among which then mated with the males. Such adult females that mated and produced ephippia or resting eggs were regarded as sexual females. Usually, the ephippia were observed on the dorsa of sexual females (Fig. 1). *D. similoides* was incubated at 25 °C, under a 12-h light (2500LX)/12-h dark photoperiod, and fed *Scenedesmus obliquus* for 3–4 weeks. As a result of the particular reproductive behaviour in cladocerans, when population density reached certain level, reproductive switching occurred. We selected and confirmed different developmental stages of the offspring using an OLYMPUS CX21FS1 microscope (OLYMPUS, Tokyo, Japan). Fifty virgin SF and 50 mature PF were collected for transcriptome sequencing. All samples were immediately frozen in liquid nitrogen and stored at −80 °C until use.

### cDNA library construction

Total RNA was extracted using a TRIzol reagent (Invitrogen, Carlsbad, CA, USA), and cDNA library construction and Illumina sequencing of the samples were performed at Novogene Bioinformatics Technology Co., Ltd., Beijing, China. The mRNA was purified from 3 μg of total RNA using oligo (dT) magnetic beads and fragmented into short sequences in the presence of divalent cations at 94 °C for 5 min. The first-strand cDNA was generated using random hexamer-primed reverse transcription, followed by synthesis of the second-strand cDNA using RNaseH and DNA polymerase I. After the end repair and ligation of adaptors, the products were amplified by PCR and purified using a QIAquick PCR Purification Kit (Qiagen, Valencia, CA, USA) to create a cDNA library; the library quality was assessed on an Agilent Bioanalyzer 2100 system (Santa Clara, CA, USA).

### Clustering and sequencing

The clustering of the index-coded samples was performed on a cBot Cluster Generation System using TruSeq PE Cluster Kit v3-cBot-HS (Illumina, San Diego, CA, USA) according to the manufacturer’s instructions. After cluster generation, the library preparations were sequenced on an Illumina Hiseq 2500 platform and paired-end reads were generated.

### *De novo* assembly of short reads and gene annotation

Clean short reads were obtained by removing reads containing adapters and ploy-N, as well as low quality reads from raw reads. Transcriptome *de novo* assembly was conducted with these short reads using assembling program Trinity (r20140413p1)^42^,^43^ with min_kmer_cov set to 2 and all other parameters set to default. The resulting sequences were named unigenes. The unigenes larger than 150 bp were first aligned by BLASTX against protein databases Nr, Swiss-Prot, KEGG, and COG (E-value < 10^−5^), retrieving proteins with the highest sequence similarity with the given unigenes along with their protein functional annotations. GO annotation of the unigenes was conducted using Blast2GO program^44^, and GO functional classification was carried out by using WEGO software^45^. The similarity searches of unigenes were performed by using the NCBI-BLAST network server (http://blast.ncbi.nlm.nih.gov/).

### Expression abundance analysis of the unigenes

The expression abundance of the unigenes was calculated by the reads per kilobase per million mapped reads (RPKM) method^46^, using the formula: RPKM (A) = (1,000,000 × C × 1,000)/(N × L), where RPKM (A) is the expression abundance of gene A, C is the number of reads that are uniquely aligned to gene A, N is the total number of reads that are uniquely aligned to all genes, and L is the number of bases on gene A. The RPKM method eliminates the influence of different gene lengths and sequencing discrepancy on the calculation of expression abundance.

### Differential expression and GO enrichment analysis

Differential expression analysis of two samples was performed using the DEGSeq (2010) package. *P*-value was adjusted using *q*-value, and the *q*-value < 0.005&|log 2 (fold change)|> 1 was set as the threshold for significant differential expression^18^. GO enrichment analysis of DEGs was implemented by the GOseq packages based on Wallenius’ non-central hyper-geometric distribution^47^, which can adjust for gene length bias in DEGs.

### RNA isolation and cDNA synthesis

Total RNA was extracted by an SV 96 Total RNA Isolation System (Promega, Madison, WI, USA) following the manufacturer’s instructions, in which a DNaseI digestion was included to eliminate genomic DNA contamination. RNA quality was checked with a spectrophotometer (NanoDrop 2000, Thermo Fisher Scientific, Waltham, WI, USA). The single-stranded cDNA templates were synthesised from 1 μg of total RNA from various tissue samples using a PrimeScript™RT Master Mix (TaKaRa, Dalian, China).

### Quantitative real time-PCR validation

The qPCR was performed on an ABI 7300 (Applied Biosystems, Foster City, CA, USA) using a mixture of 10 μL 2 × TransStart Top Green qPCR SuperMix (TransGen Biotech, Beijing, China), 0.4 μL of each primer (10 μM), 2.5 ng of sample cDNA, and 6.8 μL of sterilised ultrapure H_2_O. The reaction program consisted of an initial step for 30 s at 94 °C, followed by 40 cycles at 94 °C for 5 s and at 60 °C for 31 s. This was followed by the measurement of fluorescence during a 55 °C to 95 °C melting curve in order to detect a single gene-specific peak and to check for the absence of primer-dimer peaks; a single and discrete peak was detected for all primers tested. Negative controls were non-template reactions (cDNA was replaced with H_2_O). The results were analysed using the ABI 7300 analysis software SDS 1.4. The qPCR primers (see Supplementary Table S2) were designed using Beacon Designer 7.9 (PREMIER Biosoft International, Palo Alto, CA, USA).

Expression levels of these genes were calculated relative to two reference genes *DsimGAPDH* (glyceraldehyde-3-phosphate dehydrogenase) and *DsimACT* (actin) using the Q-Gene method in Microsoft Excel-based software Visual Basic^48^,^49^. Each sample comprised three biological replicates and each biological replicate was measured in three technical replicates.

### Statistical analysis

Two-sample analysis of the data (mean ± SE) was performed by the Student’s *t*-test for the mean comparison using SPSS 17.0 software (SPSS Inc., Chicago, IL, USA).

## Additional Information

**How to cite this article**: Zhang, Y.-N. *et al*. Reproductive switching analysis of *Daphnia similoides* between sexual female and parthenogenetic female by transcriptome comparison. *Sci. Rep.* **6**, 34241; doi: 10.1038/srep34241 (2016).

## Supplementary Material

Supplementary Information

## Figures and Tables

**Table 1:** The top 30 differentially expressed genes in sexual females (SF) vs. parthenogenetic females (PF).

Up-regulated			Down-regulated		
Gene ID	PFAM Description	q-value	Gene ID	PFAM Description	q-value
c22292_g2	Chitin_bind_4	0	c22798_g1	Vitellogenin_N	0
c18268_g1	Leucine Rich Repeat	0	c23345_g1	Vitellogenin_N	0
c36850_g1	Chitin_bind_4	0	c23476_g2	Vitellogenin_N	0
c22292_g1	Chitin_bind_4	0	c18434_g2	Chitin_bind_4	5.189E-238
c13553_g1	unknown	0	c16077_g1	unknown	1.327E-216
c13267_g1	unknown	0	c2265_g1	unknown	1.606E-194
c14229_g1	unknown	0	c20736_g1	Tubulin_C	2.782E-154
c20567_g1	7tm_1	0	c16114_g1	unknown	1.474E-144
c1950_g1	unknown	0	c21178_g1	Endoribonuclease XendoU	1.992E-124
c11892_g1	Defensin propeptide	0	c15033_g1	unknown	6.966E-114
c7151_g1	Ferritin	0	c16911_g1	Reticulon	2.035E-106
c19046_g2	unknown	0	c21985_g2	Animal haem peroxidase	3.889E-104
c16684_g1	SecA DEAD-like domain	0	c16323_g1	Tubulin_C	1.9075E-97
c21753_g2	Chitin_bind_4	0	c14544_g1	unknown	2.0145E-94
c22095_g1	FHb-globin	0	c31684_g1	unknown	3.7586E-93
c18106_g2	unknown	0	c9802_g1	Caveolin	1.072E-87
c22854_g1	Animal haem peroxidase	0	c31718_g1	Lectin C-type domain	6.5101E-84
c20987_g1	Short chain dehydrogenase	2.35E-268	c13264_g1	TCP-1/cpn60 chaperonin family	1.8185E-77
c23239_g2	Pyridoxal-dependent decarboxylase conserved domain	1.76E-261	c31673_g1	HMG-box domain	5.4632E-75
c8533_g1	unknown	5.34E-227	c22501_g1	SPRY domain	1.7373E-60
c17941_g1	unknown	4.32E-205	c23556_g2	unknown	1.8503E-60
c17991_g3	Trypsin	2.27E-191	c16151_g1	Rapamycin-insensitive companion of mTOR	1.2664E-59
c20854_g1	Kazal	9.92E-187	c16413_g2	unknown	3.332E-59
c19171_g1	DEAD	3.94E-173	c12941_g1	unknown	9.6416E-57
c20224_g1	Fasciclin	2.78E-170	c19155_g1	Innexin	2.5575E-54
c22933_g1	Animal haem peroxidase	3.2E-169	c16110_g1	unknown	4.416E-54
c23017_g1	Ligand-gated ion channel	1.3E-162	c9752_g1	Tubulin_C	5.821E-54
c19046_g1	unknown	8.23E-161	c15362_g1	Protein of unknown function (DUF1075)	1.3176E-52
c16960_g1	unknown	3.91E-148	c13754_g1	Trypsin	4.4932E-52
c3162_g1	Cysteine-rich secretory protein family	3.38E-142	c16548_g1	Chitin binding Peritrophin-A domain	5.1991E-51
