# Supplementary material for: Reproductive switching analysis of Daphnia similoides between sexual female and parthenogenetic female by transcriptome comparison
Source: Sci Rep. 2016 Sep 27;6:34241. doi: 10.1038/srep34241 (PMC5037449; doi:10.1038/srep34241)
Supplement: Supplementary Information [file srep34241-s1.pdf]

Reproductive switching analysis of *Daphnia similoides* between sexual female and parthenogenetic female by transcriptome comparison

Ya-Nan Zhang, Xiu-Yun Zhu, Wen-Ping Wang, Yi Wang, Lu Wang, Xiao-Xue Xu, Kun Zhang, Dao-Gui Deng*

College of Life Sciences, Huaibei Normal University, Huaibei, China

Additional information

Supplementary information accompanies this paper at http://www.nature.com/scientificreports

Figure S1. Gene ontology (GO) classification of *Daphne similoides* transcripts with Blast2GO program.

Table S1. Nucleotide sequences of top 30 differentially expressed genes in sexual females (SF) vs. parthenogenetic females (PF).

Table S2. Primers used for qPCR.

Figure S1. Gene ontology (GO) classification of *Daphne similoides* transcripts with Blast2GO program.

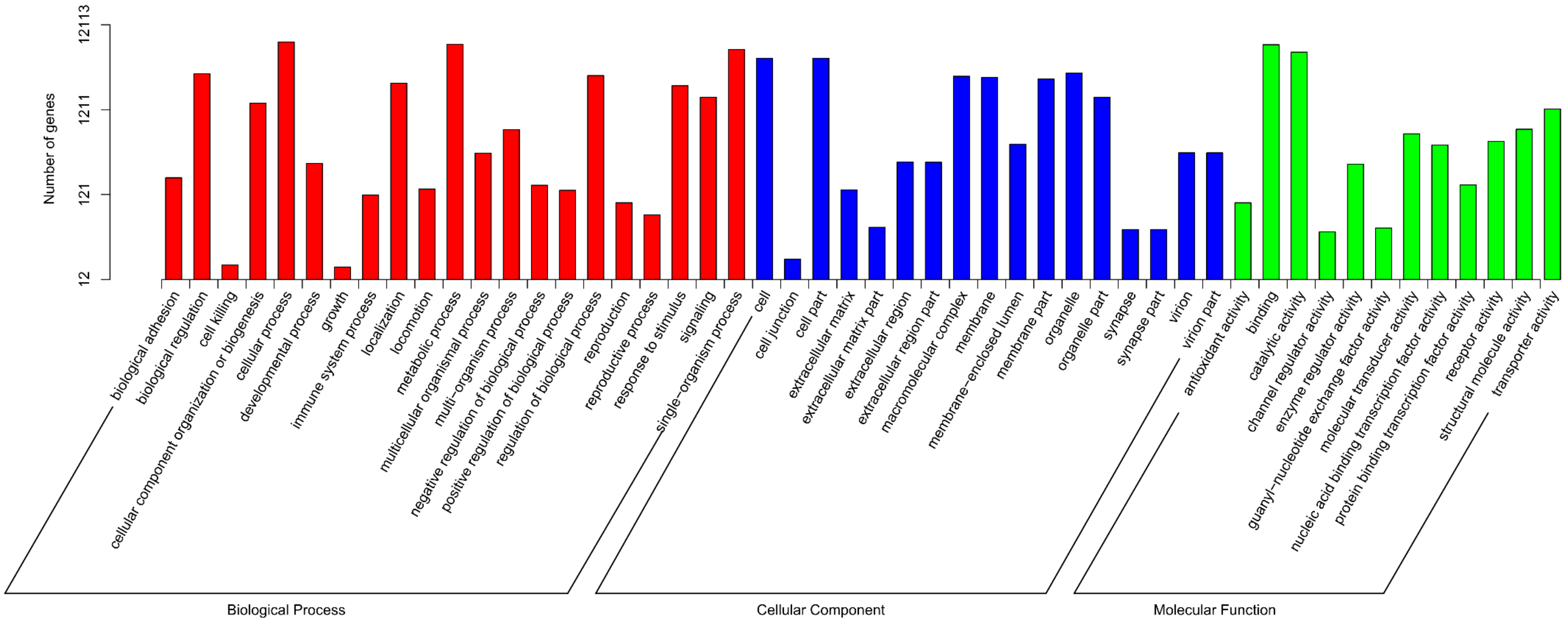

Table S1. Nucleotide sequences of top 30 differentially expressed genes in sexual females (SF) vs. parthenogenetic females (PF).

SF vs PF up-regulated genes

>1c22292_g2
GGGGGGCACAGTTTCATTTGAACTATCTCAGCTCCAACATGAAGATTTTCCTTTTGGCCGCTCTTGTCGCCGT
TGCTGCCGCTGGTGGAGTATACGACAAAGACCCCTACGCCAAGGGTGGTTACCAGCCGATGAGCTACGATA
AAGACTCCTACGCCAAGGATGGTTACCAGTCGATGAGCTACGGTCAGGACTACAAAAAAGACAACAAACAA
GACTACAACATGGGTTACAATCAAGACAAGAAAGGCTATTCTACCCCCGACTACTACCCGGTTCCAACACCC
TACAACTTCGCCTATAAAGTAAAGGATGACTATACATACAACAACTTTGGCCAACAAGAGTCTGGTGACGGC
TATGGAAACGTTAAAGGGTCCTACTACGTCTACCTCCCCGACGGCACCACTCAGACGGTCAACTACAAG

>2c18268_g1
TTTTTTTTTTTTTTTTTTGATTTTATGATTGAAATGTATTTTCCGTCAATGAAGCGCCTGTGTTAACTTCTTGTC
CATACCAAAACATTATAGAATCGATATTAAAAACCAGTTACAGCACAAGGAAAAATGTTAAGAGAATTTTGAA
GTAAACAAGTCGTAATTCCATTAACAATATAGAAACTATATTTGATGGGCATCGGCCAGCAAACGGCTCAGTT
TGACGGGGTTCAGTATAAAGGGGTTTGGCTTTGTGTCGTCATCGTTGATCGGTGTACTCGGGTAGGAAAATA
GTATCTGGAATAACCTAAACCATCGCCATACCAACGTCTTCTTCGACTTCAGCTTCCATGCGTGGTTCTACAAC
AACTGCATCCTCTTCTTCGGTCTGCTCAATCTGATCCAGGCTAGCTTCGGCGTCACTAGTGGCAGTAGCTTCG
GCTTCACTGGTGATAACAACCTCGAAGTCACTGGTGATAGCATCTTCGGTGTCATTGGTGGTAGCAGTCTCG
GTGTCTTTGGTGGTAGCAGCCTTTTGATCTTCGGTTATGTTGGCCTTTGCTTCGACAACAATATCTTTCGTTTT
CAATTTCTTATCGAAGCATTCCGTGTCGAAAAGGCACAGATCCTCTTCGTTGGAGAGTAGTTGAGAGAAAA
GCTCGGCCAAGATGATGTCCTCATTGCTGTTCGTGTTGCTATTAATGTTGATGTTGGTGTTGTCTGATTGGGG
TCGCATCACCCAAGGACGATTGGGACGCTGAGGCTTTGGTCGATGAGTCGAGGGACGCTGAGGCTTGGGA
TGTTGAGGCTTAGGTTTGTAAGGTGTTGGATGTTGGGGCTTGGGCTTGTAAGGCGTTGGGTGTTGAGGCTT
GGGGTGGTAAGGCAAGGGTTTAATGGGTTTAGGGTGGTAAGGCAAAGGACGATGGGGCTTGACAGGAAG
GGGCTTGGGGTGGTGTGTAGGCCGATATGGAGATGGTCGATGTCCTCCATACGGCGAAGTACGACGTTCAT
CCGCATGAACATAGACCAAACAAACGGCCAAGAAAATAGCTGCTGAAGCCGAGAATTTCATGTCGTTTCGT
GGAGAAGGAGCTTGGATGCTGTAGAAGAGAGGGGGAAGGCAAGTCTGCTGAATTTCCATCGATTTTTGCC
TCTTTTTATACCGAAGTGACGATGTTTAACAGTCATTCAACACGGGCATCACGCTAATTACGGTATCCATTTTC
TGAAGGATTACTTTCAAGTAATTAAAGCGTTCTCCAGTTTTCAATCGTTAAACAACGTGAACCTCGTTATCATC
TTGTCGAATTTCCTTGATTGCCATCTCGTACAGCTTCGATATTATTTGATTAAATTAACGAAGGCACCGACTAA
AAGGCCACCACTCCTTCCCCTCTCTTTGACGCATTTTGGTCTTTTCCTTTTCAAGCCTTGGAGAAATGTATTCC
AGACACTGACATCATTCCTTTCTTCCTTGGAGACCCGTTTGGCCGGCCAGTTGAATCCGGCTTGTGTTTCAC
ATCAGTTGGGTTTTTTTTTACTGGAGATGTAACGGGTGCTGGTAGAAATCAGGTATGCACTGTGCACTTAATT
TTTTTCACGTTACCATTATCCTTTTTCAACAGGACGTCAAAACTGGCTGCGCTGGACATGGAACGTGGGATA
CGTAGAATGACCGTCTCTTGACGATCTATTGCCACAGCCGTCCAAGAAAATTTTTGATAACTGTACCGTAGAG
TCAATGTTGTTAAACTGCGTTCTTTTTGTAAAATTCGATGCGGAATATCGCTAAACCAAAGTCAATAAAAAAA
AAAAATTTTGATTCGATGGACGTGAGGTTATCACAATAATCACGTCATATACAATCGCAAACATTTCCGCCGTC
TAGGCTAGTTCTCGATTTCATTGACCCAGTCGTATTCCGAAGCCGCCTTCTCCACTAAAACTTAAAAACATACA
GAGTAATACGTATTTTTTTTAAATATCTTAAAGTTAAGTTGAAAAACCCTTGTATCAAACTGTCAAATGAAAAA
AGAATCCTGAGATCATCTATGAAAATTAAATGTGCATATGCGTTTAAGAGATCGACGTGCCAATTATCGGCAG
CTGTATATTAAAACGAAAGCAGTGTTTTTGCAATACACTTTCTAATCTCTGAGAAGAAAAAATGATGCGCATT

ATTCCCTATAAATGAAGTATTAGAATTGTGTACCGCAAGTTAGAACTCTGCTTGTGCTGAACGATAGCGTTTCT
TCCCGTTCATGATAGGCAGTGGTCGTAGCCTTGAGGATTCAAATTCTCGAACAGAGTACCGTTGGAGCACCT
GCCGTTGTGCACGCGTTTCAAAAGGTGTCGTTTATCACGAATCAACCACGAGAGATGACAATCGTCACAGAC
GAAAGGATTGTTGTGGACGTAAAGGCCAGCCGTGGATGTCACGTAACTCATTTGCTGCAGCATGTCTTCGTA
AACGGCCGAATCGAACCGAGTCAGCCGATTGAAGTCGAGATAAACGAGGGCTTCAGAAAAATCTCCTTGG
AAAGCACCTGGCTCGATGTCCGTTAGGGCATTCTTAGACAAGATAAGGAACGCGACGCGAACGTCTGGACT
AAAAGCGAGTGATCCTTTAGGAATTGTGCGGATGGCATTCCCGAAGAAATTGACGTAGCTCAACTTCTTGAA
AGAACTGACACCGTCTGGGATACGTGTCAATCGATTTTCCCACAAACTCAGCTGTTGTAGAGTTTCGTTTCCA
GTTGGCGACGAAGTAATCGAATTCACTAGTTTATTCACAGGGCGATCTCCCAGCTGAGTTCCGTCCAAGAAG
AGAGATTCCAGCAGAGGCAGTCGATCAGCAATTTGATTCCAGTGCTGAAAATCCAGGCATTCGTAAATTCTC
AACGATCTGAGACGGGCGAGAAAAGCCATCGGTGGGAATCCTCGAAAATTGATGGATCGAGTAATACTCAG
ACTAATCAATCGAGTACTATTAGCCAAGAATTCAAAACCCAGCCGGCTGAGGTCACATCCAGCTACTTCGAA
ATGTCCGATGTGATGGAGCGATGATCTGAAAGCATCCGGATGGATTTCCAAACGCAAAGGGCCAGCAATCG
AGGTACAATTGATGGCGATTCGATCGACGCTTTTGCCATTCAGCAAATCAGCTGGTATCTTGATGATGTCACT
AGACTCGCTCTCGGTGGCCAAGGAGAGCAAATCTAGCCAATAAATGTGTCTAACCTTTGTGCGATTGAAAAC
TTTCCAAACGTCGAGGGCCGTCACGGGTAACTTGTCGTCTACGGCACCTCCGCAATGCACAACCAATCCGTA
AGGTTCGTAAATCTCACAGACACAAGGCGAATAATCGCTACAGACGAAAGCATCGCGTGACGTGTAATGCC
GCACGATGATGAGAGCGTCCATGGTCAACACGAGAAACAGGATAAACAGCATCACAAGAAAGTAGACTGG
ATTCAACGGGCTCAGTCTCTGCGTACCTACTGGGCCATGGTTGACGT

>3 c36850_g1
TTTTTTTTTTTTTTTTTTTTCATTGTTAGATATTGTATTTTTCTGTCTCTCTTTCATTAAGCCACGTTGCCATGATT
TAGTTGAAACAGTGACATAAAAATTCCCATTGTATCCCAAATTTAACTTATCATAAATTTATCAGAATAGGTCTT
TAGTAGCAGAAGAGGCGTCAAAAGGAGATTCAACCAGAATATTCATTAGCGTATCCAGATCCCATTTGAAGG
TAAGGGGAAGGTGGGGGGGTTGGCAAATGGGTGCCGGTAGGCTGGTAGCCATTTTCGTCGGCAACCCAG
GACAAGGAGATTTTCTCGCCTTCTGGAGAAATCCAGTAAACAGTTCCCTGGTTGGTGTTTCCGTAAGCTTTA
TCGTAAACAGGTTTGCCGTACGAATCGTAGTTCGTTGAGGGGATCAATTTCTGGTACTGGGACTCTTGGCGG
GCGGTGTAGTCAGACTGAAGGTAGTCGAAAGAGCTGCTGCCATCAGGTTTGCGGTAATCGGACTGGCTGAT
GATGTAGATACCGGAGTAATCTTGGCCATACTTTTGGGAGCCATAGTTCGACTGCATGTAGCCAGAGTCCTTC
ATGTAGCCAGAGTCCTTCATGTAGCCAGAGTCCTTCATGTAGCCAGAGTCCTTCATGTAGCCAGAGTCCTTCA
TGTAGCCAGAGTCCTTCATGTAGCCAGAGTCCTTCATGTAGCCAGAGTCCTT

>4 c22292_g1
TTTTTTTTTTTTTTTTTTTTTTTGCGGTCAAATAAATCTATTTACTTCTTAGTCTTCATACAAATAATTATGAATAA
AATATGTTGTACAAGAATTGGTCGTCAGCAGAGGCACATCATTGCTTAGTACTGGCTTAGTACTTTTTGGGTG
GTGGGTAATCGTTTTTGTAAGAGTCCGATGAGTAAGCAGATGGAGTTGGGTATTTATCCATGTACTGAGTAG
GTGGGTACATTGGTGGGTATTCAGGCTTGTATGAAGGGGTGGGTGGAGTGTAAGAAGAAGCACCTTTGTAT
GATGTTGGGGGGTAAGACGAAGATCCCTTGTATGAGGGTGTAGGTGAGGAGTAAGACGAAGCACCCTTGT
ATGGAGCCGGGCCGTAGTTGTCACCGTACTTGGGTTCTCCGGTGTATTGAACGTCAGCGACATATCCGTAAG
AGTCGGCCTTGTAGT

>5 c13553_g1
AAAAAAAACTCATTGTCGACCTTTGCAGCAGTCACAGGTCTCTAAACCTAATCATCAACATGCAGAAAAACC
TCATTAGCTTGATGTTGCTGGTGGCAGCCGTTACATCAGCCAATTTAGATCCAAACAACGTCGAGCCATTTGC

CAACTGGATTCCCAAAGAATCTCCCATCAATTACCGTATCGATTCTCGTATACGCAGACAAGTTCCTTCTACTG
ATACTAAAGAGGAAGCATCAGCTTCAAACGATGTGCCAATTATTGACGGATCTACTCTCATAACGGAATTGGC
AACTGTTCAGAGTCCTACCACGAGCGTTAAGCAGGCAAGTTCAGCCATTAGCTTGCCCGTAGACACTTCAGC
AATATCTCTCAACAGCTGCATTTTCATTAAGTTGAAACTTGTCATCATCATCAACATTCCGGATCCCGTTTCCTG
CAGCACAATCTGCGCCCGTAATTCCCATTGCACCCATATCTCGTACGAACCTCAGATGAGCGGAGGCACCTG
CACGCTGCATAAAGCTCCCGGTTTGGGTAACGAGTGGTCGAGTCCCGCACCCCGAAGCTCAGGTGCCACTT
GTGGTCAGGTCCCGAAGAAGAACAAGTGTGTTTCTGGCGACGGATCGCTTCTGAATCTGAACTTGTGCCTC
GATCTGAGCATTAGCCTAGGTCTACTTTAAATTGGGAAAATTAAATTATATCCATAGGTCTTCGCTTACGAATG
GCAAAAGGATGTGGGATTTTCAATTGGGATTTTTTTGGCTTTAATTGGTTTTGGTTTTTTTCGGGAACGATG
TAATACACCCTTTAGAACGACAAAACGACAAAACGA

>6 c13267_g1
TTTTTTTTTTTTTTTTTTCGTTTTCACAAGGTATGTTTTTTTTTCTTGTTTTTACCAGTGTGTATTAACCAAGTAT
CCAATATTAGAAATCCATTTGCTTTGCAAGTCGAAAATCTAGTCTAGCCCATAAAAATTTATAGCAATAAGAAT
TTAAAAATAAAATCGTTGGTCAGTGCCTTGATTGGTCCGTTTCAGATCACTCGGTGATTGAAGAATCTTAGAG
AAATCAGCAGGATTTGTGTGTCATTTTTGCTTGGGGTTTTAGTTTTCAGATTCCATGTTTTGCGACTCCATGTC
TGAAGTCTCGCTCTCCGGTGCAGCTGTGCGGAAGTTAGTTTCGAGAATGTCCTCATTGAACAGCTGTGTTGC
CTCGCTCGATTTTGCGTCGGACTCTGATTTTGCGTTTTCGCTTGTTTTAAAATTGAAGAAAGGATCTAAATTT
AAACCGAATGGGAATTCTGAAGGCTTCTCTGCGTGAGAACCGTGCGATGTTGGGTAGGAAGAACAATGTTT
GCAAGGCTTTTCTTTGTAGTGCTCCATGGTGGTCGGCTTGTAGATAGGGACCACTGGAGGTCTAGGCTTGTA
GGTCGGCTTGGGCTTGTAGGTCGGTGTGGGTTTGTAGGTAGGCTTGGGCTTGTACGTCGGCTTGTGTGTCG
GTTTGTATGTCGGCTTGTATGTCGGCTTGTGTGTCGGTTTGTGGGTAGGCTTGTATGTCGGTTTGTGGGTAG
GCTTGTATGTCGGTTTGTGGGTAGGCTTGTAGGTCGGCTTGTGGGTAGGCTTGTACGTAGGCTTGTACGAAG
GCTTGTGAGTAGGCTTGTACGAAGGCTTGTAGGTCGGCTTGGGCCTGTAAGTGGGGACTACTGGAGGCCT
GGGTGTCTCAATCGGAATGGGTTCCTCTGGGCCAGACTCTGCAATGCTCACCAGAAAGTCGAACAAGATGT
TATCGTTACTATTGCTGTTGTCGTTGATGTTCGTGTTCGTTTGTCCTGCATGTATAGCACTCGAATTAACG

>7 c14229_g1
ATTTTTTTTTTTTTTTTTTTTTGGTTTTTAAATGCAATTTTATTCGTTTTTTCAGTGTCTAAAAATACAGGGATC
TAAAAACACTCAACAGATTTTCCAATACGTTTAAATTAATTGTTAGAACAACGAATGCAAGTTTCAGATTTTA
AGAAGTGGTCGTTGGTAGCGTCGAAGTAACTGAAAAATCTAGTAGCTTGGGTATTTGTATGCTGGACGGTAC
ACGGGTCCTTTATTGTTACCGTAAGACATGGGCGAGTACCCGTTGTTACCATTGTAACTATCCATCGGAGATG
GGCCTTTACCGTATCCGCCATTAGAGTATCCGCCCTTTTGGTATGCATCCATGGGAGATGGGCCTTTACCATAT
CCACCATTGGAGTATCCACCGTTAGAGTATCCACCG

>8 c20567_g1 +2
CCGGGGATAGCGTCAGTCGTCAAAGCTCTTGCTTTCGAGGTCGCTTCTTCCGCCTAACTGCTTTCAAGCATT
ACAAAGTCTTTTTTCGCCCGATATTATCACTGAATTTGATTTATCTTCTTTTGAATCTAAATCTCATATCCAGTTT
GAAAAAATGGCAAACTTGACTGGTGATGCTGTGGTGGCCATGGCCCAGAAGCAGGCTTTCGATCCATGGGC
TTTGCCCGATACCTTCACCATCTATGCCTACGCCCCAGAGGACATTCGCAGCTTCCTTCATCCTCACTGGCATA
CCTACAAGGCTTTACATCCTGCTTGGTATTACTTCTTGGGATTGATGTACTTGGTCATCGGTACTTGCGCCGTT
GCCGGTAACGCCGTCGTTCTGAAGATCTTCAGCCGATTCCCAGCACTTAGAAGTCCCGCCAATTTATTGGTG
ATGAATTTGGCTGTCTCGGATTTTCTTCTGATGCTCGCTCTCTTCCCCGAATGCGTCTATAACTTCTTCCTTGGC
GGTCCATGGCGCTTTGGAGAAATGGGCTGCCAGATCCACGCTTTCTTGGGTGCTTGTTTCGGATACAACCA

GATTTTCACTTTGACCATGATCTCCTACGATCGCTATAATGTGATTGTAAAGGGATTCAGTGGAACGCCATTGA
CGTTTAACCGCGCTGTAACAATAATCACGATGAGCTGGATTTGGGCTTTGGGCTGGTCAATCTGCCCGTTGG
TCGGTTGGGGTGCGTACGCCATGGATGGTATCATGGGAACATGCTCGTATGACTACGTCTCCCAAAACATGA
ATAACAAGAGCCACATCTTGGCTGCCACTTTCGCCAACTATATCCTGCCAATCATGGTTATTGCTGGTTGTTAT
TACTTCATCGTTCATGCAGTCTTCAAGCACGAGGAAGAACTTCGCGCCCAGGCCAAAAAGATGAATGTTGCT
TCTCTTCGTAGCAATACCGACCAGCAACAGGTGTCGGCCGAGATTCGCATTGCAAAGGTCTCAATCATGAAC
GTATCGTTGTGGTTGACTGCATGGACTCCTTTTGCCGTTATCTGCATCATGGGCACATGGGGTGATGTCTCCA
AGATTACTCCTTTGGTATCTGCTATTCCTGTGATCTTGGCTAAAACATCTTGCGCGTACAATCCTCTCATTTATG
CCATCTCTCATCCTAAATACCGAGAATGCCTCAAGCAGATGTTCCCCTGGATGTGTATCGTTGAAGAGAAGAA
GACAGCAGCCGACAATCAATCCGTTATCACAGAGAAAACTGAAACAACCGAAATTGTCAAGTGCGAATCAG
CCTAAGCCTTTGGAGCCTGAAAAATGATCCTCTTTGGTGTTGGTAGTGATAGGTTTGTTGGTCATGGACGGC
TATCATTTTGGATGACGACACTGATTATCGATCTAGACCCAGCGAATGTGAATTTTACTAAGCGATTTATTATG
AATTTCTGTTAAATATCGTTCTTTCTCGTCCCAAATATTTTGTTTGGATTTTATGTTTTCTCGAAAACATTTTCGT
ATTTGACAACAAAGAGCTGTTGAATATACTTAAATGCAAAATATGGTGTTTACTCTTATATCTTCGTTTATATCT
TTCTGTGCTTGC

>9 c1950_g1
TGGCAGTGGATCCATGTATGGTAACGATGGTTACATGAAACCAAACTACAACAACGGCGGGTACAACAAGG
GCAGCTCACCTTACGGATCAGGATACAACAAAGGCATGGGCTACATGGGCTAAACCACACAGCAACTCCATT
TCAACTCACATTTAGCAATCCGACTAGCTGATGATCGATTTCAAGAATTTTATCTTCTCCAATTGGCTATAGTTG
AAGTCTCCTTAGGCTTTCTACTTGTTTCCCCCCCGCCCCCAAAAAAAAATATTTAAAAAATCCTTTTCTGTTTG
TCTAAGGTGAAATAAACAAAGGCAGCATAAAAAGCAAAAAAAAAAAAAAAAAATATGGTGACAAGATTTGA
ATAAAAAAAATATGGTAATTAATCGAGATATAAAGAATAGTGAGGGATAAACTCTGTTGGGGAGAGAGAAAG
TCATTTGTTATTTATAGCGTGCTCAAGAAAGAATCGATCTGGCCGCTAGCGAAAGTGGGGCGAGCTTCTTGA
GGGATCACCCTCGGTGCCTGCGGTTGCTGGAACTGCGGCCTGGCCTGCTGCTGGAACTGACCGGGAAATT
GCTGCTCGGCCGTCAGTCGGGGAGCCTGAGGAACACCCTGAGGTACGGGAGCGAAGACAACGGCCGGCT
GCAGACGGCGGCGTTCGTTCTCTTCGGCGATGCGCTGGCGTTCGTCGGCCAAACGCTGCTGTTCCAGCTGC
TGTCGGCGAGCTTCCGCTTCCTGGCGAGCGCGTTCCTGTTCGTTCACGTTGGCCTGGATCTGAGGGAAAAC
TGGGCGTTGTTCCTGGAACGATGGAGTCTGGAAGACCTGAGTGTTCTCGTTCAACTGAGGGAATTGCGGAC
GTTGTTCACGGGGCTGCTCACGGGGCTGCTCACGGGGCTGTTCACGAACTTGTTCCGGACGAGGGGTGGT
AGACGGAGAGAAAGCAATCAGCTGATCGCGGAATCGGAAGTCCTCCTGAGTGGCCGGGATGAACTGCTGA
GAGGCTGACTGGAACTGGTTGGACCTGGGATCGAAAACGAGCTCAGTTGCAAAGCTGCGTTGGCCGGTG
GGCCTGGGAGGTTCGGGACGCTCTTCTCTTGGCTGTTCATTGCGCTGGACAAAGACTTCCTCTTCTTCGTCG
AAAATGGGACGCTCGGCGGGCGGGGCTGGACGAGGAGCGACGGGGGTCAAGACTGGACGGAATCCTGG
CCGAGATGGAAGAGCTCCACGGATGACTTCCTGTTCAGGAGCGGCTTGCGGGCGTTGGGGTGCGGCTTGC
GGGCGTTGGGGTGCGGCTTGCGGGCGTTGGGGT

>10 c11892_g1
TTTTTTTTTTTTTTTTTTTTTGCTAATAGACATCCATTTATTCATTAAACCTCTGCAATTACAAGGACAGAATTG
AAATTCCCATCTTCTGCGATCTTACCGGTTCATTAAAAAGTTTTTCCCAAAACTAGTCCGTTCGTAGTGGTCGT
ATTTTGTCAGTGGGCTAAAGAAGATCTAAAAGTCTTTTGTCATCATCGTTTGGATGAACTTGAACTGAGAAG
CCATATCCTCTGGCTGAGGCTCGTCTTGTTGGGCCTCTTCTTCTTGGACCTCTTCTTGTTGAAGCTCGTCTTGT
TGAGGCTCTTCTTGTTGAGGCTCAGCTCGTGGGGCAGACTCATCGATGGAAGCAACTTCAACCACGCTATCA
TCAGCAGGTGTTTCGATCTCAACAACGGCTTGCTGATTGTCTTCTTGAACATCGGCAACTACTGGCTTGGCG

GTGGTTACGGCAACCGGAGACGCAACACTAGCTCGGCTTGCAACTGCTTTGGTCGCGGCTTTGGCTTTTCT
TTGTTGAATCCACGAGCTGAGGAAACTGGACAGGATGTCGAAATTGGAGTTTTCGTTGCTGTTTTGATTGAT
GTTGGTGTTGCCTGTCGGTTTGCCTTGGCTAGTGACAACGCAGACGGCCAAAACGAGAGCAGAGAGAACG
ACAAAAGTCTTCATGTTGATTCGTTGGAATGAAAGGAGAGCTGATGCTTTGAGAAAGAAGCTTGGAAACTG
ATCCCGGCTTG

>11 c7151_g1
GTTGTATGCGGATTAACCTGAGTGAGCCGTTTATTATGCGATTCTTTAATATCTAATTAACAGCATTGCAAAGG
TAACTAAAATAGCAGCTTTATTTTGGACGCGCTGTTGTAAGATTTTTCACGAGAATGACGTGAAACTAACACA
ATAATTTTTAAAATTTTCTCAGTTAACGGCTAAGGAAAATTCTTAGCAAGTGCATTCAATGGTTGTCCAAAAG
ATAGAGCCACAAAGCAATAGAACAATCGTGTTCATCGTGATCAAATTCCAGTCTAAGCCTTTGCACTGGGGA
TGTAAAATATATCATTGTATAAAATGAACTTATTGCTTAGTACTTATTGCTTAATACTTCAAATGAGATTAAGTTG
CTCGGCTTTTGGAAGAGGGAGAAGTTTTGATAAACATCGTGCTTTCCTTCTTTTCAACAAACTCAAAGAAGT
TTAGCGAGTGCCGTGAAAAGTGCAGTAGATAGAGCTCAGCTTTGTTCTGCTTTTTCTGCAGTATAGACCACG
TCCGGGTCTGGTTGTCTTATTTAATTAGGCTATAGAATCTCCCGTAAAAGAAACTGAAGGTGAAGGCCGCTG
TGAAGACTACCTCTACACTCCTGCCATCACTATGAGACAATCGTGGTCTCTTTCCGGTGGGTGCATGCACCTG
GCAGATTGCAATATTGCTCGACATCCAGCCACTCACAATGCAAACACTGTTTGTTGTGATTAGAGGTATTAGA
CGAAACATTTAAAAGAGACACCTGAAAGTTCTACAAAATTTCTGAAATAGTTCATGGTGTATCGGGAAGGGC
CACATAAAATTAAGGCATTATAAACAAATAAAACAACACATTAGGTTACGTAAATTATTTCGTAAATATATTACA
GCAAAGTTTTCAGATGGACGGAAAATTGTGTCGTCCTTTTCCAAATGCCGATCATCGAAATACTGAACCATCT
ACTTCAGTCAAACATGGTTTGTTTGTGAGTGGCAGCTAACATACAAGTCTGTAGGCAATAATTTCACCACTGC
AATTAGCAATGTAAAATTAGGTAAATGAAAACAAAGTAGTTGATGGTGATAATGGTTTTATTCAACAGCCCAA
TAAGGAGGAGCATCTGCAAACTATTATGTCGATCGACTTTAAGATATACTGGTACACATGTGCTCCAAACTTC
AGATGTTTTCTATCTTGATTCCAGTATGTATGGCAAGAACATTTAGGATTGTAGTTCCTTGTCATACAAAAAGA
CTCCAAGGCCATCACCCACACGCAACAAGTTTGTATGATGCTTGGCCAACTTGTTAATGGATTCCACTTGCTC
TTCCAAGAAGTGCTCCTCCAAATAGTTGGAAAGATGTGGGTCATTGTGACCACTGGCCACCTTGTGTAAATC
CAGCAGACTCTGATTGACTTGTTTTTCTAGGTTCAGAGCAAATTCCATTGCAGCCAGAGGAGATGCCCATTC
CTGCTGGGCAGGACGGTTGATGGCAGTCAGTACAACTCTTCCACCACGGACATTTTGGTATTTTATCAGCTT
CTGGGCATGCTCGTGTTCTTCTTCAGCAGACTCTTTAAAGAACTTAGCGAAGCCTTTAAGGGCTACATCATCG
CGATCATAATACGCAGCCAAAGCCAGGTACTGATAATGGGCATTCAGTTCAATATTGATTTGCTTGTTGATAGT
TGCTTCCGACTCCTCATGGTAGTTGTGACGACCTTTACCCACCATTTTTGAAATTTTGTAAAAAATTTGGAATA
CGTGACGACTCGAATGAGAAAAGTGAATTGGTGGAAACTGCTTGTTCACACACTGGCGAAGCAAAACAGA
TGAATGACAGTAATGCCCTGCAGGCTGGCTTTATATGCCCTGTCCTCTCCCCGCATGTCGCCATAGCAACCAA
TGGTAGTTTCTCTCACCCAATTGTAGACGCCCTTGTTGAGAAACGCCAACTCACGTTGCAATTCCTTGTTGCG
TTGTGGTTAGTAACATGTTAAAATTTGACATTTCCAATTTGTCTTTCTCTTTCATGAAAAAAGCGAGGCATTCA
CTTTGCCCCAGAAAGCTGTTTGTGTTTTCTCTTTTTCGTGTGCCATGCCACCATTTTAGCTGGTGATCTCACTT
TCGCCAACCACACACAACATAAATAAAACACACAACAATTGGCGTTAAAATGTTTTATTCAGCTTAAATGTTG
AATCGAGAAATTTAAATTAAAACCTTTGCTGTTTTCGGTATTTAAAATGTGTAGCGGATTTAATGAAACATATT
TGTAAGAATTTAGTTCAATCCAAATTCGACAGACCTGTGCCTTTGCGTGCTGATTCATACAATTAGTAATCACG
TGTCAGGGAGGATGCTTCTTGCATCAGGTTTTTAAGATGCAAACGAAATCATGGAACCACGTAGAACGTTCT
GACATGAATATCAGCTGTTGTTCGTTCTATCGACCCAAGTTATCATATTTCAGGGCAGTCGTCGGGAAATTGC
ACCGTCATAGTGACCCGAAAAAATATTGGTCACGTGTTGGCCATAGTGCCGTAGGACGTTTATTCGCTCATCT
TGGCTACAATTTTAGTTAAGCGTAAACGATTTTATAAATTTCAGACTATACTTGTTTGATTCAGTTACTAACTGA
CTATGTTGATGAAATGGAAATCTGTCAACCACTGTTAGGTATACTGAAGTCGGTCTGCAATAAAGTTGGTAAT

TTTCAAAGTTGGTTATCCAAGTGCTGATTTCCGATTGGAACTTTCAACTTGGAAATTTTGCCTTCCCTTTACTG
CGAAAAGAATGGGCCGAATTTCCACACAATCCTAAGTAAGATGGATGGTCAAGACAACATGGTGCGTTTGT
CTTGTTACATGTGAAACATTTCATGACCACTAGCACTGATAGTACAGCTGCCTCAAGGCTGATAACGAATGGG
TTGCAACACGAGCTGCAAAGCAGACTCAACACCCTTATCGTGGACATCCGATACACGATATCCAGCAAGCAA
CTGTCGGATGCTGGCTTGTTCATGTACTTATTGGCTTGCTTCATAAATTGAGACTAGCTCTGGACTAGCTGTTT
GCCACGGATTTCAAAATATTTTTCT

>12 c19046_g2
GAGCCGTAAGTTGCAGCCTTCGCCTTTGGTGCAACCACGTAGCTAGATCCTTTTCCGTAAGAGTCAGCTCCA
TAGGTTGAGCCTTTGCCATACGATGTAGTGCCATAGTTGGTTCCTTTGCCATAAGAATCAGTCTCGTAGGTTG
AATCTTTTGCCTTTGGTGAAGCACCGTAAGTGGATCCGGTACCGTAAGAGTCAGCTCCATAAGAGACACCCT
TTTTAGTGGATGGAGTGCCATAGTCGGATCCTGAGCCGTAAGAATCAGCACCGTAGGTCGAGTCTTTTGCCT
TACCATAGTCGGGAACAGTGCCATAAGAATCATCCCCGTAGATCGAAGCCTTCGCCTTTGGTGCAGCTACGTA
GCTAGATCCTTTACCATAAGAATCACCTCCATATGGGGAACCTTTTCCCTTTGACTGAGTGTCATAACTGGTGC
CTGAGCCGTAGGAGTCAGCGTCGTAAGTTGAGTCTTTTGCCTTTTGAGGAGTGCCATAGTTGGATCCTTTGC
CATAAGAATCAGTTCCGTATGATGAAGAGGAACCTTTTCCATTTGATGGAGTACCATAGTTGGATCCAGTGCC
ATAAGAGTCATCGCCATAAGTCGAAGCTTTTCCATTGGATGA

>13 c16684_g1
TTTTTTTTTTTTTTTTTTTTTGATACTAAAATTGGTGTATTACGTCATGTCCAGAAACAGATTCATAAACAATCA
AAATGAATCTCGCGTGGAACAGCCTTTTGATACTTATCAACCCTCATTGATCGAAGGCAGTTTAAAGTACACC
TAGGCTGATTTTCAAAGCGAGATCGAGGCACAAGTTAATGAGCGATCCATCGCTAGAAACACAATTTTTCTT
GGGGATTTGTCCGCAGGTGGCACCTGATTTTCCGGATACGGTAGTCAACCCGTCCTTACTCGAACCGGGTGC
ACTGTGCAGCGTGCAAGTGCCTCCGTTCTCGAGAAGTTTGTGCGAGAAGTGGGTGCAGTTGGAGTCGCGA
GCGCAAATTCTGCTGCAGGCTACCGGATCCAGGATGTTGACAATGATTTTGATTTCGAGGCCGATGAAAATG
CAAGCGTTGAGGGAAATTGCAGACGTGTCGATGGGCAACGTGAGAGACGAAGTTCCCTGGCGGACGTTAG
CGGTAGGACTTTTAACAGTTTCCAATTCCTTTAGAAGAGATGTACCGTCGATAATTGGCACATCGGAGGAAG
CTGCTGTATCCTCGTTGACCTCGGTAGACGGGACCTGCCTGCGCATACGAGGATCGATCCGGTAATTGATGG
GAATCCATTCGATCTCATCCACGACTTGTGTCGGATCCAAATTCGCTGACGTAACCGCAGCCACGAGCAACAT
CAAACTAACGAGATTTTTCTATTTCCAAAAGGTAAGAATTATTCGTTAATAAAGGTAATTAGATAGAGTACCTG
CATGTTGATAACGGGGGAGTGACTTGTGTCTGCTGCAAAAGTCGACGATGAATGTGCGTCTCGTTGGTCGG
GTGTTCACCTTTATACCATCGGTAGTTGAACGGAATGAAATTATTCCACGGCAATCGTTACCAATGTCTAAATG
TCTCATGCAAGATGTCCCAACTCTTGCGATTGCCAATTTCTTGCAGCGTCCAATGTCATATGGCAACCACGTTT
CTTGTTTTGGTTTTAGAATGCCATTCTATATTGTTAATTATTGTACCAATCCGGACTCTAATTAGCAAAGTGCTG
TAACTGAAACAAATTTCCGAAATCTAGTGATCCAGCGCTTGTTTAAATCTTACGCAACAGACCACGCATGGAA
AACCTGTAAAAACTGATTTTCCTCGTAATTTGATTAGTTCAGTTCATCTATTTTCCATTTCCAAAATGATAACAT
AAAGTCTACTGGAACCCAATTGATAATATAAAATACCTTTTCCCAAATTCTGTTTCCATACCTGTTATCTAAGTT
TTCGGCTCCATCAAAATTAAAATGATATTGCATTTGACCTGCAATAGATATTGAAAATGTAGTAGTTCATTGCC
ACTTCCATCACCCTACCGATTGACGCCACATCAAACGCTCTTGGTTTTAAAGGCAAGTTTTTCAGACGAGTTC
GCCATCATCTTCGTCACTTGGATTCGTCGATGGTTTCTTGAGCTGAAGTAACGCCTTGACAAGCTCTCGTTGA
TCCGCTTCGCGATTCTTCTCTGTTACTCGTCGTTGGTGAAAACTGTCCATTTGCAGTCGAACAGCAGGTCTAA
AGTTATTTCCAGCACGTTGTCCAGGTGTCCTTAATTTCCATAGAGTCTTGCCTTGGTCTACTAGATTTGATTTC
CCTGTTGCGCTCCTGATCCTCTGGCGCGCGTAATCAGTAATTCTGATTGGGTCACGTGATAAAGCAGCAGTAA
TGACGTAGAGGATGTCAGAAATTCCACCCATGAGAAGCACATCTTTGGCGACACGGTAGGGCAAGATTTTG

CTAAAAAGAGTTAAGAAAGCTCCAGCACTGATCGAGGCAACTCCACCGATGAGAATGAGCGGTGTTAACAG
GGTTGACAGGACCATCTGTTTGCTAGACATTTGGCGATTCTTCATGTAAATGACATGATCGAATCCCATCAAA
CGAAAAAGGCCCAATTCGCTTTCGAAGGTGTTGGCTTTTACCTTGCGGATGTCGCCGTCGATAAAGTCGAG
CGCTGCCTCTTTACTTGTCAACCAAGTGGCATCGTAAATCGCTTGCCGGCCTTTCAAGAAATCCAAAATACAC
TCGCGCTCATGCTCTTTTTCCGTGATTTCATTCACTTTTGTTTTGAGGACATCTTCGTCCATTTCCTTCACGCG
GACACCGCCACTGATGACGTAGGCGTCAATCAAGTCATCCAATATATCTCGATACGGTGTCAAAGGCAACAG
GTTGATTGCTTCGAGGCGATTCGAATTGTTTTTGCTTTCCAGCCACTGTCGTCTAACTATTTCGGCACGAAAC
TTGCTGCCCACTAAACGCATGACAGGGTCTTCGTAAGCAGGGCAGTTTGGGTATTTAAATCTTTGGCCATTG
TAGTTGGATGAGAGATTTCCGTCGTCGTCGATGATGTCTTGCTTTATCAACTCTTCCATAATTCCTCGGCATTC
GGCCGGGGTGATGTCAGCCTCACGTAAATCGTCGTCCGTCAACTGGCCAAACAATTTCAGGTCGATTGACTT
GAGTTTTACGACATCAACTTTGGCGATCTTGTTGAACTGTAATTGCTCTCGATTTGGGTTTTTCTTACGATTAC
CATAAAGCGTCTTTTCAACGTACTTTTGCGTGAAAAAGAGTTTCTTTTCTTGTCCGTTCTTGCCGTTAACATCA
ATGGGATTGTAGATGACGTAATCTTTGTCATCAAAGGGAAATGTTTTGGCCTGCTCGCGGTTTAAGTTGAAT
GTGTCAACGTTGCACTCATCCACGGCGACGAAATTCCGGTTGTTGTACAAAGCTCCGGTTTCAACTAATTTC
GCCCAGAACGACTCCCTCGTACAAGGAATTATATTGTCTTCTTCAAATTTCTTTTTCATTTCTTGTTCAATTGCT
TCTTCGATAGCCTCTTTGCTTAGGGCTTTTTCCACCGCTTGGCTGAGTGATTTTTCCAAGGTCGATGCACAGA
CACCAAAGGCATTTGCTACTCGTTCAATCGCCAGTTCTCGATTTGATTTGGCATTTTGTTCGAAGGAAGTGG
AAATGAAGCTCGGTATGGTGCTGGCGTCGTTCATCTGATGGCTAATACATTTGGACTCGCCCTCCAACAACAA
CTTGAAGTATTTCTCCACCATTTCGGGTGGAATTGTCATTGACTCTTTGGTGTTTTTGTCTTTTTCCTGGAAAA
CTTTAAAGTTTTTGTGTTCGCCTTCAATCCATTTTACAAAGTTGGTTAGATTTTTTTCGAAAAATTGCTTGACT
TTTGGCAACTGTCCTGCTGCTTCAAAGTCTTCGATGGAGCAATCGGTGCCCAGCAATGAACGGACCGAGCC
AAGGAAATACTGAAACAAATTAATCACGTTCTCCTTTTGCTGCGCATAGCCGTCGACAACACAAAACAAAGG
CGGCTGATCAGTTTTTCTGCCCATCTTTTGGACGATGGAAAAATGCAAGTTGATGTGCTCGCTCAACACGTT
TTCACACGTACGCAATACTTTGATGAATTCATTTTTGTTTGTTTCGTAGTTTTCCTTTAGAAGAACGAATGCGC
GATAGTAGTAGGCGGCCGGATAAAAATGATGGTCCTTCGATTGGATGAGTTGTTCCAAAATTTCGACTGCCT
TTGGGAGGTCCGGCTTTTTATGACGCGTAGCCAAATGTCTGGCAACAACAACTAAGCGGGCTGGTGTCATC
CACTCGACTTGGCTGATATCCAAATTGTCTTGCTCGACGGCTTTCAATTTTGGGATGAATTTTTCTGAATATGA
ATATTTTGTCACATCTAACGGCAAATTGTTCATTTCGTCCAGCCACAAAGCCCACTGATCCAACACGCTGCCA
CAGATGGCACTCACTATCTGATCACCGTCATTTTTCTTGCGCCGTTCTTCTTTGAGGTTGGTATAATATTTGGA
AAACGGATCAATTAATTCCATCTGATCTTTCATAAACTTAAAATCTTCCTTCATCCGAGCGATGCGTTGTTTCTC
TCGAATTTCGCGCTCATTTTTCATTATTTCTATCGGTTCCCTTTCGTCAGGCGGCCAACGTTCGGACGAACTTG
GTTTGCATACAATGAGGATTCCACTTCCAGGTGCTCCACTGCGAGCTGCACGACCCATCGCCTGTTCCTCTAT
TCGTTCGTTTTCGGGGAAATAGCTGAGACACACATGCAGTCCTCCATTTTCGCGTAGTTTTTGGCTGATTTTG
ATGTCCGTTCCTCGTCCAGCCAAGTTGGTTGCAACGATAACATGTTCAATGTCCAGTTCTGTTCTTTCGAATG
CAAATTTCACATAATCGCGCGTGTAGCGATGAAGCCGGTTTTTCTTAACCAGATCAGGAATCGAGTTCACTAA
GCGTTGATGGACGAGATTGACATTTTTGATGGACATACAAAAAATGATGATTGAACGTTCTTCTTTCATAATA
GTTTTGGTTTCAATGATGATGGCATCTAGCCATTCATTTTCGGTCGACAGGACTTGAGTTGGTTTTTGTTCAA
AAAGTTTAGGTATGTGCGTATGATTGTAGTGGAAAGTGGACTCGAAAGTATCCTGTAAAAAATGTTTTTCTTG
TTCGGATCCCAGCGTTCCCGAAAGACCATAAATGTTAGGATATTGATTGATGTACGTGGCATTTGAAATGAAA
ACAGCTTTGAGACTTTGCAGCGTCAATTTACATCCTTCCTTCAGCTGGACGAACTGGTGCAAGGCTTCGTCC
CATTGCGACAAAGTTTGATCCGTTCCAGTATCGGGATCGATAATAATCACTTGCGGATTGAGATCCGGACTGG
TGTCTGTTCGATCTTGATCAATGACGTAGTCCTCGTCCGGTCGTAGATCCAGTGCCAGAAAAGCGCTATCCAG
CCAAGTGTCCAAGTGTCGGTCAACAAAGTCGAGTAGATGGGCAGGTATTCGAATTTGACGTTGGCGATTAG
CCACTTGGCGTAAATAGAAAACCAATTTGGCATTGAGATTTGTTTCTTTTTCCGCATTCTTAGGTGTATAGTTG

ATTTTGTCTTCAGTTATTCGGTTGGTATCGGCAATTAGTAGACGTCCTTTATCGTCAATTACTTTTTTCTTAATC
AGATGATTCCACAGCTCATCTTTTTCCAAATCGTTTAATGGCGCGTGAACTAATTTCAAATCGTCTTCAGTTAT
GACACCGTATAGGTCGTACAATACGCGAGATTTAATTGATTCCTTCACTTGTTTTCGATAGTCCGGGATGTCCT
TATTAATGCGAGACTTGCATATTTCATCCCAAATGAAAACGTACAAAGATTCCAAAGTCTCCATGCCGGGAAT
GCCGTGTGATAAGTAGAGGGTGTTGTTGCCACGATCCAGCAACATGCAATCCACTTCGTCAATAATGACATG
CTGGAATCTTCGATTGCCACGACTGTCGCTGCCGTAGAATTTGTCCAACAAATAATCACGTTGAAAATTGGCC
AATTCTCCATACACCACAGCTGAATCGTAGGCTTTTGTGCGCTCCTCTATTGAATGGCTGCAATTGTTGGCCA
CGCTAACGTTGAAGAATTCATAGATGTCTTTCAGCCCTCCCTTGTCCGACGACAGGGTGGAATCCCGTAGGG
CTAGCACGCTGTTGCTTGTAATGACGTCGATTTGAGAGAAGGACTTAGACTTTTGCTTCTCTGAAAGTGCAA
ATGCGATGGCCACTCCAGCAACAATTAATGACTTGCCCTCGCCAGTTGACACTTGAACCAAAGCATTGTGAG
AATTCGGGTGTTTTCGCAATAATGTTAGGATCGCTACTCTTTGGGTATCACGCAAAGGAAATCCTAGTTGCTC
TTGTACAGCGAAATTGTAGACGTGGAGGAAGTTTCCTAGCTGACCATCACTTGTTAGATCTAATCCATTTGTT
GACCACATTTCTTTGATCAGTGTAGCTATTCTATCGAATCTCGCCGCAGCTCGCCGCCCTTGAATTAAATCTCG
TTGCGACAGTATGGCATCGTTAAGATCCATTTTTTTGGAACTCTGAACAATTTCTTTTATGCGGCTCAGAAAG
ATATGTTCCGAAGCTCCTTTCGAAATTTGAAGCTCTCGCATCAAATCGCTGATTATATAGTCGACGTTTCTCTCT
TCAGGCGTTTGGCTTGGGTCAATTGGAGAGTTTAATGTCGAGGCCACGTCTTCGCGTCCAGTTACAACTTGC
TGTTGGTAAGCCAGTAACGGGGAAGAGTCTAGATCATCAGGTTTGAATTGCACTTGATAGGCTGGCTTCACC
ATTTTTGCTAGTTTATTGTCCATTTGAAATTGTTGGCTTCTAAAACTCTGCTGCCGGAATCGGATTTCAATGCT
TTCAACTTGTTTCGCCAAAACTTCGTCCCTGTTAATCGGATGTGATTGTAATGAGCTCGAAAGTATCGCTTGG
AGAGTTTGGAACATTTCTTTGCGTTGGTTTATCTGCGAAAGATGATGGTAGAGATTTTGGCGCAGGATAGCC
TTCTTCTTAACAGCTTCGACCAGCCTTTTAGTTTCAGGAACACCCTTTTTGATCTCTATCGATGTATACCTCTTC
AAAGGGTTTCCTCCGTTTTGGACTTGTTTGGCACACGAATTTTCGCCTTCTGTTGGTTTCTGGCTAAAGTTCA
ACTCCAATCTAGCATCGTTTTCAAAGCCGAAATAATTTCCTTCCACATTCGTGCGGCAGTTGTCGCTGTTGAC
GCTGTGAATGAAACCAATGTCACCGGCAGGTTTACCAGTTTTACCATCAGAACCGGAGGCTTGATAGACATC
AGACATTCGCGTGGCGTATGGTATGCGCTTCTGAAACCCGATAAATGGAAGGCTAGGCTGGACAAATGGGG
ATCTGTTCAAAATCTCCAAGGTCACAGTACCACCGTATCCACCTTGTCCGACTCCTAGCCCTTGGAAAAATATT
AGCGTATGCCTTTTCTTTTCTGTTTTGTAAAATGATACCAGGAGTTGCTGGACATCTTCCATAAATTCTTCGAT
TGTGAAGTTAGGTTGTTCGAGAAGTCCCGATATACGCTCGCCCGGCAAAAGATTTTGCAATTTGGTAGCGAT
GATTTCCTTATTTTGGTCGCCAGTGTCGCCTAATAATGACATGGACGGAAAAGCTTTTCTAAACTCGTCTCTA
GTCCATTTGTGAGCAGCACTTCCGTCGCCTCCGTCAGATATGATGGTCCATTGATTTGCGTTGATCACCTCGTT
GCAAACAAGGTGTACATTGCCACCACTTTCACCTGGTTGGCGTGGTTCCTCTGCTTTTTGATGAGTCATGTGT
TGACCAGACACGTTCCACGATACTTCACAGTCTACAATGATCTTGTCAGTGATGACGCCTACGTTGATACCGT
GCCAGATTTCATTTTCCAAGTCGCAATCAACGTGAAGAGACACTAATCCAACAATTTGAATTTCTTGTACGTT
GTTGCTTTTCAATTCAATCATTTTCGCCATAATTTTGCTGAGAAATACAACATTTCCTTGGATAGAAATGATTTC
CCTTCCGTCTTCGTTCTTGACTAGTACCTCTATTTTAGGCAGCAGGTATTTCTTGAAAATCTCTATCACCAGTG
ATGTGAATTCCCGCGTCCAATCATCATCAGCTATTTTAGCGGCTGCTAGTTTAAAGTATTGGATCACACTCTCT
TCGTTGCAGTCGGTCGGGAAATGTTCGCAAATCATCTTGATGCATGCCGGCGGATCTTGAAGCACATTTTCG
GAAACGCTCTCCTCCATAAAGTCCTGGAAATCCTTGCACTCCATGACATTCGCCAGGAAGTTGTTTAATGCG
GAAAGGAAGTTTCTGTATTTCTCGCCATTGGTTTTGTTATTCACGACGTAGTTCCAGCACCGAGAGAAAAGC
TCGTTGATGCAAAAGATCTTGAAGTGATTGAAAAAGCAGGGAACGAGAATGGTAAAACGATGTGTGTCTTT
AAACAGCTCTTCCTGTTTGACGATCAACTGAAGATACTTGCACCCTCCATCACACCAAATTCGAAGTCGTGAT
CCATGTACTTCTAGTTTTGTATCAATTTCTTGAAACGAATTGACAGAATTCAATTCACTTAGGAGAGTAGATAG
TTCCATGGCAACGTCAGTCGCTGGGTTCTCCAGATTGTCAAACATTTTTCTCAATGTCTCTTTATGCCACATCT
CCAACACGATTTTCACATACTGCCATTCGAGACACGTCCTTTCGAGAGACGAAAGCTGACGTCCCAATTCCG

ATTCGATATGACTTATAGCATTGGGGAAAAGCTTCAGATCGATGTCTTCATTATTTAACTGAATCCAACCTTGA
ATTTCTTTGGCAACATCTTCTTGTGTCCTATATTTCTGCTCCGGTTTTTTCGGCCGGGTGTGACTTTGTCCAAC
TGCGATCGCTCCATCCTCATCCATGTTGACAGGTTTTCCTTATAACACAGTGAATAGATGCACAGTAGTTTGTA
ACTTCTAACTGATTTCAACGATCTTGACGTCTATGACAAATTTCCTCTAGGTTTCCCCTTGCACACACGTAGTA
CGCACCGAAATCGGTGAGCGCCAACAAGACGGTGACATCTACAGGACAGGATGTCATAAATCCGCTTGCGT
CATTGGGTTTTCCACTTCTAGAATTCGGCGTAAATAAGATGTCGCAACGCTACACCACACATTATCATTGAGA
CTGTGTCATCGTAAAACAGTTTAGGCAACTCAAGACGTTGATGTTCAAGTCTTGAATGCAGAATTTGTTCTGT
AAGAAAATGTGCTGTTCCAGCTGGGCATGCAACAGCCTCCCTTTGCTCTGCTGGTCTGCTCTCTAGCTGAGA
TAACACCGAACTGAAAATGAAAGGCTACGTCATCTAGCGCGGTTTGTTATAGCGGCCCCGAGGCTTCAACAC
AAACACAACTTTTACGACTTTGACGCAGCTGGCCTGCAATAAAAAAGAGTCCGTGTTGGCAAACTGGTATTC
CCTAGCAACAGCTGCGAATTTCTGTACTGGATGTTGAATTAGATAATAGGCTGAAGATGCTTGACGGCCTTAC
CGAAAGACGAGCGAAGATCACGTCCACAACAGCCATCTATCGTACAACTGAGCAATGCTATAGCATTAAAGG
GTTGGATGAGATGTCACACCTTGGCACATCAAAGGCCTAGCAGAGGATAAGTTGATTCTATTGGTATTCGGG
GGCTGTAGCTCACGTTCTGCTAAAAAGCCGGAACGATTATTTAAAAAAGAAAAATTAGCTTCAAAGTTAAGG
TCTTGCGACAGAAGAAGAGTATACGTCTGTTCCGTAAGATTTTCATCCAGTAAAAGAACATGGCTCCTGTTG
AGAAAGTGTTCAAATTCAATGTCCAGTCTCACTCTAGTGTAAAAATCCTGCATAGGACGACAGTTGGACATTT
TTCAACTCACCAGGTTTTAGGCGGAAAAAAGATCAATCCGTTCACCTTCGCCTGCGAGGTGATTGACAATGA
AACAATTTATGACTAAAAGGATGATGAATCAATGAAATGAAATTCGATAATTCTTTTTACCACATGTGATTCGC
CTACCAATGTCACGAATGTACCAAACTGAGTTGATGTTGCTAGTCCTTAAGGTGCTTCATTATTACCCAAAACC
ATGGAAATCCTCATCACACCAAACATTTGCGGTCAGTGAATAGTGGGTGTTCGGCAATGCGCCGTGCCTAAA
TGATGGTACAATCGGCCATACCTTTCTTATCTTGCTCAGCTGGTTCATCGATCACAGGTGGGTAGGCAATACTC
CATCTTCCATCATATCTTTCAGCATGGAGATGACAATAATCCAAGAGGGCACTAATCTGCCTTAACATCCTTCA
ACTAGCTTTGCTTGAACGTGTAAGCAATTAACTCGTTCAAATACAGTTGATCAACATACTCAATTCTGATATTA
CACCGATACACCAGGTATTCACGTTCGTACAAGTTCTATAAAATGCGATGGATTTTCCCTGGAAGAACACTGT
CTGATGAGACTCTCGCGCAAGTAAGAAGTCAATGTCAGCTAACAAATGGCTCGTTTCTCTGCAATTCAATATG
GTGTTTTGGTACTGATATTTTCGACATATGTAGTAGCCAGTCTAACTGTAGATATGCAACTGGAGCAACTGAG
AAATAATTACATGAAACTGGAGCAAGTTTTGGCTGCAAAGGAGAGCCGGTTAGAATTTCTGGAACAAAAGG
TTCAACAGCATGGAAATTTAGAAGCTCAAATTACACGACTTGTGAATGAAATGAATCAAATGAAGACTCAGG
TAAAGCGGACAGCCGAGCTGGAGCTCAAAGTCCAACGACAGGAATCCCTTCTAAACGCCGTGCAAAGCGA
AAGTCGAAGCCATAACAACGTTAGCCTTAGACAGGGAACAGCACAAATGCCCAAATCCTGCGCAGACCTAA
AGAACAATGGCCACACCTCAAACGGAATGTTTTTAATTATGGGAGTGAAAATGGTGGAAACTGTTTTCTGTG
ATTTTTCGAAACCTGTAACCGATCCAAGCTTCCAGAAATGGATCGGTTTTGCTGATGTTAAATCCTCTCCAGT
CTACTTCCATGCCCAGAGAATAGGAAACTATGGGGCTGCCAATACTGCCATCCCATTCAATCAAATAAGGTTG
AATGTTGGGGGTGCCTTGAATCCTGCCACTGGAGTGTTCCTTGCTCCAAAATCGGGGAGATATATTTTCCTCT
TCTCAGGCCTGAGTTGGGATAGTAATGCTAAAGTTCAGTTGCAAATGAAAAGTGCAACTGGTGATTGGTCCA
AAATTGGAGAAGCTCACTGCACACCATACAATACTTTTGCACTACAATCAAGTCTAGAACTAAAGAAAGGAG
ATGAAATCAGAATAATCCACATTCACGGAGTCATCCACGACAGAGATGGCAACAACTTTAGCAACTTTGTTG
GCTTACTCCTAGAAGAGGATGTTTTTGGATGTTAACAAGCTTGCAAATGATTATTGGAGGACTGTACGCTACC
CAGTGGCAAGATAGGAGTATATATTGCCTATACTGCCTCTACCATGCCAGTGGTTCCATGCGATCATGTTAGCA
ATGTGGAATTAATTTTTTTTTTTTTAAGTTTTTCATCTATGCATTTAATCTACTGTCTATTCTTTGAAGTTAATGG
AACACGGGAGGGGGGAATAAACGAATGCGGGGACCTTAAAGCTAATTTGTCATTCTTTGAAGAATTCAGGC
TAATCTTTAACAATATGGCCAACAACCAACCTGTTCTATTGAAAATGCAATTGACACAATGGCGGGAAAACTC
TCCAGAAACTCAGGAAACTTTTGCGTTTGATCAGCGAATGCCAGTTAGAAACCAGGACCTCCCAATTATCAT
CCTAATAACAACTGAACTTCTCTAGTTC

>14 c21753_g2
GGTGCATGGAGGATCAGTTGTTCAACTCGTCATCCCCAAGACAACATCATGAAACTGTTCGTCGTCGCCGCT
CTTTTGGCCGTTGCTGCCGCCGCTCCTTCCAGCTACGAGCCGGCCTACAAAGCCCCTGCTCCCTATGGCGCT
CCTAGCTACAAGGATAACAAGTACGCCGACATCACCGTCACCAGCCAATCGGACGAACGCAACATCGATGG
CAGCGGTGCATGGAGCTACGCCCAGTCTGACTACACCACCCGCGATGAGTCCTACGCCCAGAAGAAATTCC
AAGGCAAAACCTACGACTCTTACGGCAAAGAATCCTACGGCGAAGTCCAGGGACACACCAACAAGGGATC
CTCTTACTGGGTTTCCCCTGAAGGCCAGAAGTTCACTTTGACATGGGTTGCTGATGATGCTGGATTCCAGCC
CAAGGGTGACCACTTGCCCGTCCCTCCCGTCCACGTCTACGAGCTCCCAGTCGCTCCCGTCCATGAATACGA
ACTCCCCGTTGCCCCCGTCCATGAATACGAACTCCCCGTTGCCCCCGTCCACATCCCCTTCAACGGCAAAGGC
TTCAAGATCTACTAGATCCTTCCTCTTAAACTGATTAAATATATAATATATAAATGATTTCCTATACGTTATCTTTC
CCGGCATAACATTGAATGTGTTGTTGTGAATATATACACAGTAGATGCAAAAGCAAAAAAAAAAAAAAAAA
AAAAA

>15 c22095_g1
TTCGTGCCATTTGTATTCAATAAATTTGGACTTGGAAAAGATTTTTCAGAATAAATTTTCTTTTGACATGTCCA
TATCATATTTAACTGTTTGATGATTCTGTTTGTAAAACTCCAATCGAGGTTTGAACCCAAGACCATTGAATCCG
TACTCCAATACTCTACCACAGAGCTATGATACTTGACTAGTTCAGGAAATTTGTTATATGTATATTTTTTCCCCC
ATTAAAAATTTAAAACCTACACCCTAATTATTTGAAAAAAAAAATTAGAGAACGTTAATGTCGGGAATGAGTT
CAAACAAACACAAATCGATTTGCGCATTACTGAGACCTGCGGAGAGCAATGTCGCAAGCTGTCAAACCATCT
TTCCAGGCTTTTCTGGCTTCAGTAGAAAACGACTGTCCAAGTTCTTCTTCCATGACACTGAACATGACTTCAC
CAAATAATTTAAGCTCGTCGGGTTTCACGTCTTTGAAGGCGGGAGAATTGAGGTAAGGGCAGCTTTTCGGG
TTTTTGCCTAGGTTGTCAATGTAAGCATTGAGGCCGGCCAAGCAGGTATAGACTTGATTCAAGAAGTGACTG
TTGGTGGGCAAATCAGCCAGATCGACGTCGGCAAAGGCTGCAAACTTCTTTTGGGTTTCGGGTTTCAGTTT
GAAGTAACGGATGAATGCTTTCGGGGCAATGTTTCCATTTTTCTTGGCGACTTTCCAGGTGGCCTTGATCAA
AGCACGATCTCCTTCCTTCATCGAGTATTCACGTGTTTCAGGCTTGGCGGTGTGATGGCCGTAGGGACATTT
GACGCCCTGCTGACTGTATGGACATTTAGCTCCTTTTCTCATATACGGGCATTTTGATCCAACCTTGGAGTGAT
AGTGACCGGAAGATGGTTTTTGGATTCCGAGAGGACAGCCGCTGGCGGATTTCTTGAAGCCATGAGGGCA
ACCACTAGCTGAAGCATCCTTACGCGTGTATCCGTAAGGACATTTGCTTCCGGATACCATTTGTTTCTTAATTC
CAAATGGGCAACCATGTCCAGAACCTGAAGACACGAAACCTAATGGGCATTTGCCATCGGTTGATTGAGCG
ACCGAGAATCCGAAGGGGCATTTCGAGCCTTCGACCAATCCTCGCTTGAGCCCGAATGGACATCCACCAGC
ATTTTTCTTGAATCCGTAAGGACAACCGGAAGCAGCAGCAGCTTCTCTAGTGTATCCATAGGGGCATGGGGT
TCCTTCCACTGAGCGTTTCTTGAGTCCAAGAGGGCATCCTTTTCCGGAAGCAGTTCCTTGGAAGCCTAGCG
GGCATTTCCCATCGGTGGCTGCCTCTCTCGTGTAACCAAAGGGACACTTTGAACCTTCCACTAGTCCTTTCTG
TAGTCCAGTTGATGTGGGTTAATTGATTAAATATTTTGAGGTAACACAATCAAAATGAAAACGAGAGAATGG
TGAAACAGAATGCTTTACCTTTACACCAAGTGGGCATCCAGAATCAGATTTCTTGAAATTGAGGGGACATCC
AGGTTTGATTGCCGATGCAGAATAACCAAACGGACACCGTGTGCCTTCCGCTGAAGATTGCTTGAAGCCAA
ATGGGCATGGTTCCCCAGATCCGGTCTTGTTGAATCCGTAAGGGCAGTTTCCGTCATTGAATCCAGATTTCG
GGTATCCGTAGGGACATCTGCTTTTGCTCTTGTAGCTGATTGATTTCGAGTGGCTGTATGGGCATTTATGGGC
AGCAGCAGTTGGTTTGTTGAAGCCGTAAGGACAACCAGAATCAGATTTCTTGAAGCCATGCGGACATCCGT
TAGTTGCAACATCACTAGAGCCATATCCGTAGGGGCATTTGCTTCCGGCAACCATTTGTTTCTTCAGACCGAA
GGGGCAGCCGTTACCGGAACCAGTTGGCTTGTATCCAAAAGGGCATTTGCCGTCATTAGCCGCTTCAAAAG
TTCCGCCGTAAGGGCATTTCGATCCAGCAACGAGTCCTTTCTTGATTCCCATTGGGCATCCGGAAACGCCTTT
CTTAAAGTTATGGGGACAACCTGGCGCAGCTGCATCTTCTCTGGTGTATCCAAACGGGCACTGAGATCCTTC
AACCAATTGACGCTTAAAGCCTAAGGGACAACCCTTTCCAGTTGCGACTCCAGAAAATCCGGCAGGGCATT

TTCCATCACCGATGGATTCTTTCTTGTAGCCATAGGGGCATTTAGATCCTTCGACGAGTCCTTTCTTAACACCT
AGGGGGCATCCTTTGTCAGATTTCTTAAAACCATAAGGGCAGCCTGGTTTTGAAGCCAATGCATTGTCATATC
CGAATGGGCATTTTGTTCCCGCAACGGTAGATTGCTTAAAACCCCATGGACATCCAGCACCAGAGGCCGTTT
TCTTAAATCCGTGGGGACACACTCCGTCGTTGAAACCAGATTTCGGATATCCATATGGGCATTTGCTGATTTT
CGATCGAATACCGGAAGATTTCATGTAGCCGTAAGGGCATTTGGTGACTGGAACAGCAGCTTTGTTGAATCC
ATAAGGACATCCAGCACCATTAGCAGATTTCTTGAAACCGTGCGGGCAACCTTCAGCGATAGCCTTGCTGAA
TCCATATGGGCATTTAGTGCCAGCAATTTGTTGTTTTTTAAATCCAAATGGACATCCTTTTCCGGAACCGCTAG
CCTTAAAACCTAATGGACATACGCCGTCTTCAGCAGCCTCTCTAGTTCCGCCGTAAGGACACTTGGATCCTGC
AACCAGTCCTTTCTTGATACCAAGCGGGCATCCTGTGGCATTTTTCTTGAAACCGAATGGGCAGCCAGTTGT
AATTGCATCAAGAGCAGTGTATCCGAAGGGGCAGTTAGATCCGTCCACTTGATTGCGCTTGAAGCCCATTGG
GCATCCTTTTCCAGTAGAAACTCCGGAGAAACCAGCTGGGCATTTTCCATCTCCAATCGCCTCTTTCTTGTAT
CCGTAAGGGCATTTGGATCCTTCAACTTCTTGTTTCTTGATGCCGAGAGGGCAACCTTGGCTTGATTTCTCG
AAGTTGTAAGGGCATCCGGATTTCGATGCAAAGTCCTTGGTGTATCCAAACGGACATTTAGTTCCGCTTACC
GCTAACTTATTGAATCCCCATGGGCATCCACTTCCAGGATCGTTATTCTTGAAGCCGTGAGGACATTGTCCAT
CATTGAATCCCGATTTCGGATATCCGTAAGGGCACTTGCTCAGACTTCTGGAACCAACGGATTTCGAATATCC
GTAAGGGCATCCTGCACCCTTTTCAACTCCTCCAATGGCACCGTGGCCATGATGAGGATTAGCCCCGAAACG
ATGACTGTAGGGGCAATTATGGGCGTTGACAATCGCTGCCAGGCAGACAGCAATCAAAATGAGAATTTTCG
TAATGCTCGTCATGGCGCTCATTCAAAATTAACTTTTCAAAAAATTGGACTTCCAATTCGTGCTTGGATGGTC
GCTTGTAGCCGGACCAATATCTGAAGCTCCACATCTTCGTCTTCGCTTTATATACCCTTGCACAGTCGAAACTG
CATTTCCACATCATCTTCGGCGGCCAATCTAAAAAATCGATCGGGTCAATGTTTTTTTTCCCCTCAAGTTCTCC
AGTATCCTGTTGAGTCGTTATTCGTCTAGAGGCTTCGGATGGATGTTTGTCTAAAGCAACTGATAAAAACTCA
TGGTACGCCTTCTTTTTCTTGTGTGATTTCTTCCCAAATGTTTTGAGAGGATGTACCAAGGCAAGGATCGTTT
TCTTACGACGCACATTTCTTTTTCCATTAATTGATGAGACAATTTTCGGATCGCGATGGATTCTCCGTTGGTTG
TTCTCGAAAGGCGTAGATCAACGTTATCGCGCAAAAGTTTATCTCGTCCTTGCCCAGTTAAGTTTCGTCAGAA
CAGCTGCTGTCCTCTTTAACCTAAATGGAACTTGAGCTATTTCTTAACTTCAAAAACAAACAAAATATAGTTTT
TATTTTGTAGAGGAAGACGAAAAAAAGTGGCGTAACAAGCACGACGTCACATGCAGTAAATGCGTCTTAGA
AATATAGATCAATGAGTGCTCTAAACTAATAAGTTAGCTCAGAAGTTTCCAAGGCATATTTATAATTTAAAAGA
ATTATTTCAAGGAAAATTTAAGCTGTTTCCCATGGAATAAACAGCTATGAAATGATTCAGCAAAATTGAACTC
CCATGACTTCGTTCATGATTGCTTCAGCGATACGTACATTTGTCTATCTTATGTTTTAGGCAAAGGATAAAGGT
CACGGGAAGATAAATAACGAAATTAAATCTAACAGAGAACTGTCTCGTTTGATTTTTGATGAGAAGGAGGCA
GCATTTCAATGACCATATTAACGAGCTTATATAACACCAAATGGAAAATTTATGGTCCTTGATTTAGCCCTGAC
ATTCAGATATTTGCTGATGTTTCCAGGGTAGGATAGGACATTTGATTTTACTCAATTTAAGTCTAAGATTTGAC
CCAGGAGAAGCCAATCCCTTTCCATTGAGTGATTCGAACCTATGACGATATGAAAGCCCAACTTTTTGTTTGA
CAGCCTCATCGGCTAACAAATATGCCTTAGATTAGGACGCCCACATAAAAACAAAGCGAAGGGCTAAGAACC
GAATTTAGCCCAACGTTCTCATTAACTTAAGGTGAATGCTAAATTCTTAACACTAGAGCACTAAATACATTAAA
CGTAGGTTGCCAGAGGCCCATAAACGGACAACCAGCACCAAAATGTGAGATCAATTACATTTCCTCTTTCATA
TATTGAATGAATTCGGTTGTAATGCAGTTGATTTAAGATGATATTTCTCATATTAAGGAGGATGTACACATCAC
AAGTACACCCTTTGTACTATGAGAAGCCATTTTACCGTAAGAAAGATTTGGTTTTCTATGGATTTGAACCTAC
GACTTTCGCCGCGCCACGCCAATGTGATAACCACAATACCGTGACTCTTCATATATACGCCGTGCAATGCTTG
AAGTCATCAATAAGAATGAATGCCACCTTCCCAATTACTATGACTTGGTTTTTATTCTAGCTCTAAAGAAGCTT
CTCCTCCTTGTTCTTTGGCGTTGGCATGGCAACAGACATGACCAAACAGGTATCCATCTGGCGCGTCAGCTT
GAACGCTTCTCCAGATGGAATGAGAAGGCTGTCGTCGCTTTTGAGAATGACTTCTTTGTTATTTACAGTAACC
GAAGATTCTCCTTCCAATTGCCACAACCATGTTTCATAGCTAGAGGCTTGGAAGTCATCGCTTCCTTTGCCAT
AAACAACAACGTCCGATTGATAATTGATGTTGCCAAACAGCTTCTTTTTGCCTTCTTTGTGGATTTCATCTCG

GTGTAATGCGAGCCAGTCTTTCAAATCGAAAGGGTTTTCCACCTCTCGGTTGGAATCTGGTTTGTACGGTGG
ATTCTCCGGAATAGTTCCCGGAATAGGACGACCTGTTCGGCATTGCTCGGATGCGAAAAATTCTTTGATGAC
GGGCACTAATTGCACACCTAGATCATCGCAATAGAACCACTTTTCAAATAGCCCTTCGGTTGAGTCGTTCACA
AAATATCTCAGGCAGTCTAGTTCATGTGTAGCTCTTTCTCTTTCAATGACTAGTCCAATGGTATCCTCTTTCCTT
TGAGGGGAATGTGGAATTTTTCCAGGCAATAGAAATACCTCACCTTCTCGAATGGGGACTGTTTTAAACTTC
CCTTTTTCCAGTATAATCAACTCCATTGCTCCCTTTTTCATGTAGAATAATTCCTCACCTTCTTCCAGGTGGAAA
TCCTTGCGTTGGTTGGGTCCCCCAACAAAGAAAACTTTCAATTGCTGTTCATGCATGAGTTTGTTGCAAACA
GGTGGCAAAAACGAGGCTTTGTTTTCTTCGATCCACTGATTAATGTTATAAAACTGGGCCGCCATTTTCGTCT
GTAATATTCGTGCACTTCACTCGACGTACAGCTCTCGAGACGAGACTGACACAAAAACATATTTGCTTTGCCG
AAATAGCGACCCTGAAATGTTTTCACCGTCACAAACAGTCCCACCGCTCAAGGTTAAAAAGGAGAAAATCTC
AGAGAACAATCGCCTTAGGTCTTGATCGCCCATCCTCTCAAAGTATGCTTTCAAATTTGGCGTTGCGAGGCC
ACGTGTACACAAAAGGCGAACAGAAATGGAATGCAATTTCAATAGCTCAGCAGCAACGTTTTGGCTCTTGA
GAAAGGAAACTTAATTAC

>16 c18106_g2
TTTTTTTTTTTTTTTTTTTTCTATAATTTGTTGCTTTTATTTGAAACTCCTCTACTACCATATATTGCAATATTCCTC
ATCAAGAAAACAAGAAAGTTGTGTCGCTGGTTTCGATTGTCGATTGCCAAAGTCCATGTCGTAATTGCCAAG
ATTTAATTTTTCTTGACGGTGATTTCCTTGGCAAGTTTGCTTTTCTTGTTCCTTAAGAATTGCTGCAAAGATGC
GAAAGCGGAACTGAAATCAAAGTTGGAGTTTTCGTTGTTGTTGGAGTTGAAATTAAAATTGCTGGAGCGGC
GTGGTAGAAAGCTTTTGGCATTACCCATAGCTTTTTCGAGGATTGGCTTGACAGTCTCGTCAGCAGCAGTTT
TCTTAACGGCTTTTGTTGATTTGGCACGAACATTAGCTTTAATGACTTTCCAGAAGCAAGAATCATCATCGTC
GTTCTCACACAGATCTTGGTCTGAGTCCTGGATGTTGGCAAGCAGTTCAGAAATAAGTGAGCTGAAGAGAA
AATTGCTGTTTTGGTTGTCGTTCTCGTTGATGTTGATGTTAGTCTGTGATGACGGACGACCCATCATCGATCGT
TGAGACATCATTTTAGCAATGGGGAGCGTAGTAGCATCGATGGCTGATGCCATGCAAACTAGAACGATGATA
GCTGCTGATGCCGTGA

>17 c22854_g1 Peroxidasin [Zootermopsis nevadensis] 0.0 58% EFX83452.1 +3
GCCTTGCAGTGTGAAATAAGACGCCAAACGACGTTGAATGGTGTTCCGTGACGCACTCGACTTGTCTCAACT
TCTTCAAACAGTCTCATTTGAATTGCAATTCTCAAATTCAAAAGTCCAAAATAGCAAAACGGTGCTCCGACTG
ATTACGACAGCGAACTAAACAAGAGTGCTCAAGTGACAACGATCCGTGAATCGTCATGGCAACCAGCAGCA
CATTCCAGCAGCGCTGGGTCTTTGGGTTCTTGCTGATGGGAGCGATGGCCGTCGTGGCCCAACCGCCCTTC
TCGCACAAACACAATTTCGGTAGATTACCGCAGCAACAGTTGCAGCAAGTAGGACCCAAATGCGCTCTCGTC
GTGCCCAAAGGCCACGGAGAAAGTTGCATCACCATCGAATCAATCAACCGGGCATTCAACAAAGCTTCAAA
AGAATTCCGACTAGTCGAATCAAGAGATGAAACGTACAACGAACAATTGGCCGAAACTCTTCTCGAAACAA
GCCGTTTGCTGGCCTTGGAGCATAAATTAGAGACGGACGAGATTCAAGCGGCGTTACCATTGATCGATGTTT
ACAAGACGGGCATCGCTCGCTATTGCCCCAAACCGGCAAAGTGCGAACCGAGGCGGTTCCGCAACTACGA
CGGGACGTGCAACAATTTGGATAATCCCAACTGGGGCGCCATTTCAGCCCCATTCCGCCATCTTCTCCCGCCC
GACTACGCCGACGATATCAGCATGCCGCGCATTTCAGTCACCGGCGAACAGCTGCCCACCGCCCGTTACATT
TCGTCCACCATCCATCGCGATCTCGGCTATCACGATCACGCCGTCACCGTCTTCCTTCCGGCTTTCGGCCAGC
TCATCGACCACGATATGGCCGCCGGAGCTGAAACTAAAGATCCGAGGACGAATGCCGAACCAAAGTGCTGT
GACGTGTCCCCCGATCGGCGTCACCCCGCTTGTTGGCCCATCGACATCCCAGCCAACGACCCGTTCTACTCG
CTCTTTGGCCGCCGCTGCTTGGAATTCGTGCGCTCAGCCTCCGGTTTGAAAGACAAATGCAAATTGGGACC
GCGCGCCACCTACAACACGGTGACCAGCTTCCTGGACGCCAGTTTCGTCTACGGCACAAACAAGGAGACG
GCCCACAAGTTGCGCACCTTCCGCGGCGGCTCGCTCAACGCCAACCCGGCACTGCGCAATCTGGGCCTGAA

GGATCTCTTGCCATCGAGAAAGGAGAACCCCGATCAAAATTGCAAACGGCCCAGTGGTGACATCTATTGCTT
CGAAGCGGGCGATGCGCGAGTCAATCAGCAAGTCATGTTGGTCACGCTGCACACCGTTTTCTTGCGCGAGC
ACAACCGAATCGCCGCCCAATTGGCCAAAGTGAATCCTCACTGGGACGATGAAAGAATTTTCCAGGAAACG
AGACACATCATCGCTGCTTACGTCCAACACATCACCTACAACGAATTCTTGCCCATGGTGCTGGGCAAAGAC
ATCATGGCAGACTACGGACTGCTCCTCGATAGAGACGGTCTGTCGTCGGCTTACAACCCGAAAATCAACCCC
AACCTTCCCTCGTCGTTCCACGCAGCCGCTTTCCGCTTCGGACACTCCGTCCTACCCTCCCAGATTGAACAGT
GGAGCGTCACCCACAAACACGTCGGTTCACGAAGATTGAGCGAGTTGTTCAACCGGCCGTTCGACATGTAC
CAGGGCGGAGTGACCGATCGTTACATTTCCGGATTCATGAATCAAGTGGCACAGGCTGTCGACGACGCCAT
GACCGAAGAGCTGACCAACCACCTGTTCGCAGAGCCCCTCAAGAGCTTCGGTGCCGATTTAGCATCGCTCA
ATATGCAAAGAGGCAGGGATCACGGCGTGCCGTCCTACAACGCCTTCCGCGCCTACTGCGGACTCCGTCGG
GCGCGCCATTGGGACGACCTGGCTGGATCGTTCACCAACGAGACGCTGCAAAAATACGCGAGAACGTACG
CCAGTCCGGACGACATCGATCTGTGGTCAGCCGGAATTTCCGAACGACCTTTGCCCGGCAGTATGGTCGGC
CCCGTTTTCGGATGCATCATGGGAGAAACGTTTAAAAGCTTGCGCTATGGTGACAGATTTTGGTACGAAAAT
GGAGGATGGCCGAATTCATTCACGTTAGAGCAAGTGAACGAAATTCGACGAATTAAACTGTCCAGACTCTTA
TGCGACAACGGCGACCGCATCGAAACTGCCCAAGTCTACGCAATGGTTCTACCTGATCCACAAATTAATCCG
AGAGTGCCCTGCAAAAGTTCCGTCCTGCCCCGCTTGAACTTGGAATTGTGGCGGGAATCGAAAGGAAAGC
CCAATGTCAAGAATTCGCAATTTGCGCCACCGACCGCTCCCGGACCATTCGCCGGAGGCTTCAATAGCGTGC
CAACACTGTTCAACAACCCACGGCCTTTTTTTCCATAATTGATCCGAGCACAGAGGAACGGAGAAGAGCAT
ATCAAATCAACGAAACGTGACACGATAAAATTGAAAAACATCCGAGTGAAATGAAGCAGCTCCTCCGCAGT
TAGAGCCATCCAGTATACGAGTAAAAAAATCGGATCAAGAAAAAAAAATAAAAGAGAAAAAATTGAGCGTT
GATGGGAAAGAAATTTTAAAATAAAATAATTAAAAGATAAAAAAAACTTACGGAAAAAGAAAGAAACGAAC
CGCAAAAAAAACCAATTGGGTTTCGTATAGATTATATCGTGTATGATTTTTAAATAGCTATATTCTTTTGGATGA
ACTTTTAAACAATTTTCTTATTCTTTTCGGTCGGTTTTTTTTTGTTTTTTTGTCAATGGGGTTTCCCTCAATTTG
TTTTTGCCCCGTTTCAAAACCAATGCCACAAGTTCTATCATTCATTTTCTTTTTCTTTTCGTTAACGTTTACTATT
CAATTTTTGTTGTTTAACTTTCTCCCCCCCCAAGAGCTTCTGCAAAGCCAACGTGAAGCGATCAAACCATAAC
AGACCAATGCCGTACATCTTCATTTGAATTCCATCTCTTTCGCGCTTTCTGTCTGGAATTATTCGATATCACTTC
ACGATGGCCACTCTCTTTTCCTTTAGACAAGGGGCTGGGCTAAACACTATAGCCACTTGACTATAGGAAAAC
GGGATTTTGCATGAAAACTTTATCCAGCAAGCAAAAGAGACTCCATAACACAAAGGCCGCCTGTTCTTTAAC
TTCCTTTTTTGGTTTTGAAACGGAGGATTTTTTAAATTAAATTTTTTTTATTGTTTCTTTTTTCTGTCCAAATATT
TAAAAAGAAAAAAAAAAGTAGAAAAAAAAAAGATGTTTTGATATGACGGATTGGTTTCATCGTTTTCTGCTT
TAAAACAAACACACAAAAAAAAAATGTTATTCAATTTTTCAACCCGACTTTGTTCGCATGTGTAATCTCGCTG
TAATCTCTTTTCGTGAGGCCACAACAGCCGGACGAAAAGAAAACAAGAAAGAAAATATATATGCATATTATAT
GACGGCGTCGTTCTTACTACGGCAAACAGTATGTCATGGTTACAATTGCTGGCTTGCTTACGCAACATATAAA
TATGCATCTCGGTTGTTG

>18 c20987_g1
GGGGAAACCATTTGACAAATTTATTTCTGGCCGATAAGGTTGTTGTTGAAACTACAATGTCCTTGTTGCAAA
AAACTACGATGCTCCTGTCCCATCGGGCCCTTACGCCTCTTTGTGGCACCTCTAGCGCCCTCCAAAATGCTAA
GCAGCTTAGTACCTACGCTGAAGTAGCCCGGCAGAGAAGATCCCTCCAGGGTCGAGTGGCCGTCCTGACCG
CGTCAACTGAAGGAATCGGATTTGCCATGGCCCAACGGATGGCGTTAGACGGAGCTCACGTTGTCATCAGC
AGTCGGAATCAGGAAAATGTCGACAAGGCGCTCGAGAAACTGAAAAGCCAAGGGTTCTCCGCTTCCGGAA
TGGTTTGCAACGCTGCTGTCAAGGAAGCTCGTGAAAAGCTGATTGCAAAAGCAGTCGCAGACTTTGGAGG
ATTCGATATTTTGGTATCGAATGTTGCCGTTAATCCAGAGGGAGGTGGACGCCTTATGAAATGCTCGGAAGA
TACATGGGATAAGATATTCTCCGTCAACGTCAAGTCTGGTTTCTTCCTGGCCAAAGAAGCTCTTCCCTACATG

GAAAAGAGAGGTAAATCATCGATCATTTTCACCTCATCGATTGCGGCATATATTCCAAACTACGGGGTCGACT
TCTTGGGACCGTACGCGATCAGCAAAACAGCTCTCCTGGCCTTGACCAAATTAATGGCTTTGGAATTAGGCC
CAAAAGGAATTCGTGTTAATTGCATCGCCCCTGGTTTAATCGATACCAAATTTGGTGAAGCGGCTGTCAAAG
ATGAACGAGCTCAACAACTTCTTAAACTAATTTGTCCGCTAAAAAGAACTGGCACACCGGAAGAAATGGCC
GGTTTGGCATCGTTTTTAGCATCCGATGACTCGTCGTACATAACTGGAAGCAATATCGTTGCTGCTGGTGGAC
TTCACTCTTCGCTTTAACCACCGATATCACCATAGCATAAGGTTCACCATTAATTTAATCTGCTGTTGTCTTCCA
GATACAGTATCCAAAAAATTGGTTTCATCAAATCAAGGTCCACTTTGTTTGGTATCGTATTAATATTGGTTTTCA
ACAGATGAAAGTTGTGTAGATCAAATCAGTTGATCTCCCTTTGCACCTCATGGTTGTTGATATTTTCTATTTTT
GGCAACTACATGAGTATAACTTAACATTCTGTTTGACTTTTTTCCAAATAACTCATTTAAAAATTTCAATTTTCA
GATTTTGTCTAAGATGGATGTCGACATACCTCGTAATATTTGGTTTTTTAATTACATCTTGGTTTCTTATTGTTCT
CTTTCATCGATCGTTATGTTAGGATTCAATTTCTGGAGAGTTTTTTAACATTGGGGGTAGTTTGAATTTGAAAT
TGGTTTGTGTCCTGCAATTGTTGGTCGATAGATGGCCAAGGTCGAAAGCAGTGCAACTGCAGCGCCATTGC
AGCGCGAACCGAAAACGTGGGTGGCATCTGCCATGCCATTCAAACGCTTCTTATGAGTTTAGTGAACGTTTG
TTTTCCCCTACTTCGTAATCTTTGCCAGTATAGTGTTATCTTTCTTAGTTTATTTTTAAAAGTTTTCCACTGCACT
CACAGCGCATAAAATTCCCTGTTGGAGCAGGATACTAATCATGATTTAAAAGGAAAACGACAACAGCTATTG
ATTCATCTAATGAATTTTCTAGTGAATTTAATTGCCAGAAATAACCATTGATTTTCTTATGAACAAAAATCTATG
GGGCATGCCAATGAAAATCAGTAAGAGATTGTGGATTGCATCATATAACTAGTCAATAAAGTGTGATCCATCT
ACAGAATTACGGTATGGAGCCTTTGGATAGAATTCTTTATCTATTGAATGAAGAATCATAGACAAAGGTATTG
AATGCATAATCAAGGAAAACTGCGCATGTATTTACCGTCGTTCAAGATAACAACGTCGATAACATCCACTGAA
GTATGTAGCATATATACACAGAAGATTGAGCCATGTAAATGAAACTGACCTTATGGAAGGGAAATTCACGAAC
GCTCGCCGAATCTGGTGAGTTGAATTCACTGTTTGTTGAAGTTTTTTTAACTACTAGTTCGGGCAGTAATAGG
GCTGAAAAGATTCATCTTAACGAAATGTGTCCTGGTCGTACGCCAGCCTCATCTTTTAAGTTACTCGACTCAT
GTTCTCACGTAGCTCCTCATCTAAGCCACTTTAAAAAATCATAGTGGAAAATGAATAGAGGTTGATAATTTGAT
GCGAAGGGAGGAGCTAACGAGTAATGTAGGCGGGTATTCGAGAAAAAAGGATTGGTGAATGGTTGGAAC
AGACTTTGTTATTGAAACTATTTTTCATGTTATTCAAATGTATTGAGTTCAGTATTTTGTACAGGACAGCAAAG
GTGAAAAGCTAATCTACTTTTCCCCAGGCACCATTACAAGTACCTTGAGATGTAAACATCCGTAAGATGTGGA
AAATGGGTGGATTCAATCACGTGACCAAATCTTTGGCTTCGAAGTCAAGTAGTATATATACTAGTTAGTACATT
TCAATAGTCTCAAGTCCTTGAAACTTAATCTTCTTTTCGTATAAACACAGGGGCAGGTTTTCCCAACTTGAATT
GGTCATGACGACGTCAGCCACTCCTCAATCTACGATTCGTCCAACCAAGGTAGGCAAACCACATTTGGTAAT
AACATTGACAAGCCCGTAGCCTTCACGTCACAACTGCGTCAAAGCAGCAGGTTTATTCAGTATGGATGCTCC
ATATACATTTCAACCTAATGCCTTGCAATACAGATATGTGGTTTTGTTGGCACACCACTTACAAAGTACAGTAT
ACAGTTGATTTTGCTTTTTAAAAATTAGCTGCAAAAATTTGGCAAGTCGGCCTTTTCTATTAGATAAGGTAAA
AGCCCATGTGACTACTAATTGATACTGTCTCGTCCCTATCACCATAGAGTCTAGCCTGTTAAGTCACTTATTAAT
TTATTTCGTCATCAGTTATGTCAGTATCTAGGGTTTTTCACGGGCAATGAGAACGACGCGTGCCCATCTTGTG
GGAAAATCCCAATAACCCTGAAGCAGAACGATTTGCTCAACTTTGGTACAAGTCCAACTGATGGAACCAATG
GAAACACAAGAGGTCTTTGCTGATAAGGTTCCTTATCATTCCATCATTTTCTCAGCCTCTCTTGGTATATAAGC
AGCAGCTAAAAAAGTCAGAAACAGCCAAGCTTTTCCAATAATCAACAGTTGACTGTCATTTGTCGTACAACT
CTAGAAAAAGCCATTCAAGATGCTGAAAAACGCAACTCTGTTGTCTCGTCGCATTCTCCTGTCCCCAATTGCT
GGACAAGATGTCAGACAAATGACCACAGCCGTCGAACTGGCTCGTCAGAGGAGATGCCTGAAGGGTAGAG
TAGCTGTTCTCACAGCATCAACTGACGGAATTGGATTTGCCATGGCCCAGCGTTTGGCAGCAGATGGAGCTC
ATGTTGTGATTAGCAGTCGCCATCAGAAAAATGTCGATGCTGCACTCGAGAAACTGAAAAGTGAAGGTCTG
TCCGCCAGCGGAATGGTTTGTCACGCCGGTGTCAAGGAAGACCGTAACAGGTTGATTGAATCAACAGCCGC
TGAATTTGGTGGATTTGATATCCTAATTTCTAATGCTGCCGTTAATCCGGATTCCGGACGTCTTCTAAAGTGCA
CTGAACAAGTTTGGGACAAGATCTTTGACGTCAACGTCAAGGCTTCGTTTTTCCTTGCTAAAGAAGCCATTC

CTCACATGGAGAAAAGAGGAAAAGGATCAATCATTTTCATTTCTTCCATTGGCGCCTACCTCCCGAATTTCGC
CATTGACTTTACCGGAGCTTACGCTTTGAGCAAAACTGCTCTGCTTGGTGTAACCAAATTGTTGTCACAGGA
ATTGGGTACCAGAGGAATTCGCGTTAATTCCATCTGCCCTGGTCTCATTGAAACTCGATTCGGCGATGTGATT
ACCAACGACAAGAGGACACCAACACTCATGTATCATAACTGCCCAATGCGAAGAAACGGAAAACCAGAAGA
AATGGCGGGTTTAGCGGCATTTTTGGCATCTGATGACTCGTCGTACATTACTGGAAGCAACATTGTTGCTGCT
GGTGGAATGCAATCCAATTTCTAATCGCCTCAGTTTTTCACTGTGTCATTCTACATGTTCCATTTCCCATTTATT
GTTATTTACCAGACTTTATACCTTTCAGTGAAGTATTGTAAGCAAAATCAGACTCAAAAGAATAAAGTATCTTT
CTTCGATTTTCAAGCAAAA

>19 c23239_g2
ACGGGCCAAACCTTTTACATCCTTCGAAATATTTTCCATAGAATATGGCGTGATTAAAAGTACACGATCAAGTT
AATTTTTAGCCCATCTTTCAATACAAAAATTGATTGAATTAGATTTTGTGTATCTATGGAATTAACTTTGCAGTT
CATTTGAAGTGCCAAACAGTGTAAGCCAATCAGCGCCGTCACATCCGTTGATGCTTTTCTTATTAAAATGTCA
TTATTTACAAGAGTGACAAGTAAACTAATAAGAGGGGCAAAAGAGGCTAATGTGCTTTCTCATTGGACATGT
CAATGACTCAGGAAGAGAGCAAGGATAATGAATACAAAATCATGACTTTCAAGATGGGGTGTTGTCTTATTG
GTTTACAATACACTGAGTGCTATCAGACAGTTCCGATAAGAGAATGACTTGTCATCCCTAGTCATTTACGGTG
GGTCATGAAAAGGGAACGTCCCTCTGTTTTCATTGCTCATTTAACGGGTAGCAACTAACAACATCACATTGTC
TTCTTTAAAAGAGTCTTAAAATATCAACATTGGCCCAAAATATCGATATCTTTTGAATTGAAAAAGGAAAATG
GACTCTGAACAATTTCGCGCAGCTGCCCATCAAATGGTTGACTACGTCATCGACTATCTGGATAACATTCGCA
ACAGGTAAGTTCCATACGCTATGCATTCAACGTGTACATTACGTTACACTGACACACGTATTTGCTTTAATGAC
TTATCGCACTCTGGAGATATGACTGCCTAACTAACCAATCTGTGTCGGGGTTGCCCAGTTTTCTTAACGATTTA
AAATGTGAACATTGTTTTTTAAAAGGCGTGTTTTACCGACGGTAGAGCCGGGATATCTACGTAAACTCATTCC
CGAAGAAGCTCCAGAACAAGGCGAAACGTGGCAATCCATTTTTCAGGATATCGAACGCGTCATTATGCCCG
GCGTAATTTACATACCAACAATTTTGAAATGAGACTTCCTTTCCAATGTTTTTCAAAACAATTTCCAGGTGACA
CATTGGCATTCACCGAATTTCCACGCTTATTACCCAACGGGCAATTCATGGCCGGCCATGCTAGCGGACATAC
TTAGCGATGCCATCGGATGTGTCGGATTCTCATGGGTAAGAACACGATAATTAAATAAATAACCTTGTGTTATA
ACCCAAATATAACTCTTAAACCTTGGAATACATACTCAGATCGCTAGTCCAGCTTGCACGGAACTTGAAGTGG
TCATGATGGACTGGCTGGGCAAATTGATCGGCTTACCTCCTGTATTTCTGGCCAGCACAGGCGGTAAAGGTG
GTGGCGTCATCCAGGTATACAAATTGACTTACATCCACTGGGGAATGCAATTTTAAAAAATCCGCTATTAAAA
CAGGGGACGGCCAGTGAGGCCATGCTGGTCGGACTTTTGGCGGCCAGATCGAAGACACTCAAACGACTCA
AGGCCGAGCATCCCGAAAAAGATGAGAAAATATTAGCCAGTCGCTTAATCGCGTATTCTTCAGACCAATCGC
ATTCGGCTGCGGAACGGGCGGGACTCTTAGCCGGCGTTCACGTCCGTGTCCTCCCTACCGACGATCAACTTC
ATTTGCGTGCCGACGCACTCCAGTCGGCCATCAAAGAGGATACAGCCAATGGCAAAATTCCATTTTTCGTGG
TTGCTACTCTTGGGACGACACCTGCGTGTTCTTTCGATGACATTGACGAACTAGGACCTGTTTGTAACGATCA
TCTGTTGTGGCTGCACGTCGACGCAGCTTACGCAGGTTCGGCTTTTGTGTGTGAAGAGTATCGTCATTACAT
GAAAGGCGTTGAGAGGGCCGATTCGTTCAATTTCAATCCTCACAAATGGCTTTTAGTTAACTTTGATTGCTCT
GCCATGTGGTTTAAAGATGCGGATGACATCGTCAGCGCTTTCAATGTGGATCCGATCTTCTTGAAACACGATC
ATCAAAACAACGCACCAGACTTCCGGCATTGGCAAATACCATTGGGTCGACGATTCCGGTCGTTGAAACTCT
GGTTCGTGATGCGATCGTACGGCGCCCAAGGGCTTCGCGATTACATCCGCAAGCAAATTCATTTAGCCGAGC
AATTTCACCAGATGTTGATAGCAGACGATCGTTTCGAATTTCCAGTTCCTCCCTCCATGGGACTCGTTTGCTT
CCGTCTCAAGGGAGAAAACAGTTTAAGTGAGAGGCTTTTGAAACGCATCAACGAGGCCGGCCAAGTGTAC
ATGATTCCGGCTAAATTACACGAGACGTACATCATCCGCTTCGCCGTCTGTTCACGTTACACGGAACTGAGCG
ACGTGCAAGCGTCTTGCGAAGAGATCCGACGTCACGCCAACGACATTGTCCGCGCGTAAATCAAAACTTAG
AAAACATGACACTTAAGATATTCAAACCTCCCCCTGGAAACTTGTTTCTTTTCCTTGCTTAGCGAAAACGGAC

ACAACTGACTCTGCAATTTATTATCAGCGCTTTTGAAAGCTGATATACTAGTAATGTACAAAGTTCCAATACAC
AAAAACTGCCACGTCATTAAACCCACCAAAAGCTTTGGAATGCAAATGGCGGCATTTTTTTCACCATTTTCTA
ATTTTAAAAAGCCACAATGTCGAAATCGGGATATCTTTTAGCGAAGAAAGGAGTTAAACTGCCCGGTGGTAC
AACTCTGATGTTCAGCTGGCCTTTAGACGGGACGACAATCAACTGGACAGTGTCCGGAACGATGCCCAGAC
GTTTGAGTTCATCGTAGACGTACTCGCCATCGTTACTCCGAACATCACAGATAGGAGCACCGCCAGCTTCTCT
GACGGGCCGACTGCAGAATTCATCGACGAGCCGGCCATCGATGATATTCGGCATTTGACCTTGCACAAACG
GAAAGAGGGCAACGTGAAATTCATTGAAACTGAAGGCGTGATCAGCTGATAGTTTGGAGCGAGAGGGTAA
CGTCCCGTTAGTGTGAACAGCAACGATCCAGCGGATTGGTGGAGGATAGAGGGTGGATAACACGTTGGCC
ACTTGATCGGCAAGTTGTTCAGCAGTCGTCACATTCTGCCACGTTTCGTTTAATTGGCGTTTGAAGCAATCAT
TGACCAGCTGGATATTCCGGGCTGCTATTTTCACGTTACGTTCTCTTACGGCTGTTGTCCTGCGATTAAACTCC
ACGCGAGACATTCGTCCCAAACGAGAGAAGTGCTCGACGGCCATCGTTCCATTCGTAATTAAATCGTTGTAC
CGCTCGAATTTATCCCACATCTCTGTGACGTTGCACCGATAAATCAGATCCGGCCACAGCTTGACCCAGCAAT
CCTTCCATCAGTAACACAATATCGCCATTGAGGAGACGGGCACGTTCCAGCCACAACAGTTTCTGCAACTCT
GAAATGTCACTTTGCCGATCGTTTTGCATGACGCCCATGTCGATCAAAGCTCTCGAAACATCATCTTCTGCTTT
CTTGTATTGGCTTGCCTCATCGGGACGGATGAGATCTTCCAATCGCCTGGCACCGTCCAGCAAGTCTTCCTCC
GTCGTCGGTTGAGGTAAGATCCGTTCTTCGATCGCATCGATGATGGTGAATACTGCATCAAACATTTCTAACG
CTATCTCGATGATATGACCAATTTCAGACCAGTTTGCGCCTTCGGAGTCATTTGCAACTATATCTTCCATCTGTT
TGTCCAGGTTTTGACAACCAGTCCAGCACAAGACTAGTAACAATAGCGTCGTTAATGTTTTGGCCATTTTCGT
TCTTTTTTCTACACGATGGTCCTCATTGAAGTATCCGAAAGTAGCACATGTCAGCACTGTATCTATTCCGGATG
ATGCACTGTGAGGATCACATGAACAACTGACGTCAGAAAGCAAGGTATCCTCTACATCTGTTCCCATATTGGA
CAAGGCAATAACCCTTGACCTCAAGATGACACACACACCACAGTTAAGCTTGACGTTTCAGGGTTCATCGAC
TCGTTCCATTTGGGGAGTACAAAAAAAACTTCTTTGTTGTTGGTCCGATTCCTGGAAATACACACTCTCGGG
AGATGATTTCAACGTAGAGTTACGACGTAAACGCAACGACTTTTTAAACGCAGATGATTGGAGTCTGGATTC
TTGTATGTCTCGTCTTCTCCGCCTGGGGCGGACCGATGGAATTGGAGAGTCTCAAGGCCCAGCATCAAAGC
CCGTTGGATTCCGCACCGTCAGAGATCCATTCAAAGCATCAGCTGGAACAGAGGAGTCAAACGTCTGGCGA
GATTGAAGAATCGGGTTCGTTCGAACTGGAAAATGTTGGATCGCCCGAAACGTCTGAATCCGACGAATCGA
ATGAAACTCTGTCTGCATCTCATCCGTCATCGAGGAGTAAAAGACAGGCTGCCTCTTCCTACGTCCGATCTTA
TCCGAATCCGAGATCGTACTTGAGAAAACAGCAACGTGCCCCACGTGACTATTGCGACTATTTCGATTACAGT
GACGGTGGCTACGCGTATTGCTGTTATTATTGAATAAACAAATTAGAGTCCTGTTTTGCTGTCCATTGTGTCCG
CAGTAGTTCTGGTAAAAGGATGAGTCTCAAAACAAATCAAATGAAAAATTTAAGAAGGTAAAGTCTTAAACT
ATTACCTGTGAATAGAACTGGGGTGCAAAAAAAAAAATAATAAATTTCACAAATTTAGTAGATCCTTTGTTTT
CATGACTGAAATTTCGAATGAAAGTAGCGACCCCATTTCCATATTTCTCTAGAGTACAATTCTGTCCATTGGAA
AGTTATAGATGTCAACGTTTAAGTGGGTTTTTCTATCTCTAGTAGGCTATTTTGTCCAGTCAGTTGCATCCAAA
CGGTTAGTCTCTCTCTCGTTAACAAATTCAGTTAGTCCTAATCTTATTGAGAAAAATCTTAATTACAGATGATTC
GTGTTTACTTGATCGCCGTTCTGGCCGTTGTCGTGCTGGGCCGGCCATCCGAAGTGGAGCACATTTCGAAA
GCGTCGCATAACCAACGCGATGCTCCGGCTAATGTTCCTCCGGGCAAGACGGAACGCGGTCCTGAGATCCT
TCCGCCACCTGGTGGCGACAGACGTTACGGACCACCGGCGATATCTCAAAAATGCGAAACAAAAGACGAC
GGATCGCAAATAAAACAGATTTCCCATCCCCCCCAAATCGAAAAAATTCAATCACAAAAACAAACGGGAGG
AGTGAAATTGCCGGAAGCGGGACCAGTTCCGGTGTCCACCGAATCAACCCGGAAGCGAAGATTATTTTTCC
CCATCGCCACTTCTTACGCAAAACCTTACCGGTACTACCGCAGACCTTACGGGAGAACAGGCTATCTAGGAT
ATTACCCGTACTATCCATTTTTCTTCGGCGCATTCGGCTATCCGTACGGATACAGTTACGGTGATTATTACGGC
GGTTATGGAGACTATTACGGCGGATATGGGGATTATTATGGGGGTTACGGTGATTACGGAGGCTACGGAGAT
TACGGGGGTTATGGCGATTATGGAGGCTACGGAGATTACGGGGGGTTTGGTGATTACGGTGGTGGATTTGG
CGATTACGGAGGCGGATTCGGGGATTTTGGAGGTGGATTTGATGTTGGGGTCGATGTCGAATAATAAAACG

TGTGCAAATCGTTTGCTTTCTTCCTTTTTGTTACTGTTTTAAAGAAAATGAATTGATTAACCGTCGCAATTAGA
AAATAAAAACGATATCCTAACCTCATGGCGAACTGCTTCCTAATTCGTATCCCCGCAACGTTTCCATATTTACTT
CCTACGGTTTGGCTCGGACGAAATAAATTTCTGTATTACTTAAAAGAACAAAAACGATCGCTTCCTCTTGAAT
GTACAAAATGAACTGAAAAAAAAATTTCCTCTTGTCGCAAAGTCGTAAGTGTAAAAAGATAACAAATCCGGA
AAGGTACTCACGATTTTACGGTCCCGGTTCAGCGATTCGCGCCAATTTCAACAACGGCTGGAACAGATGAAT
CCACAACGAGACGGGCAGACTACGGTTCAATCTTTCTTGGACGGCTGCTTCATCCAGGTGAGACACAAACT
CTGGAAATGGCTCGGGAAATCCGAGCGAACGGGCGTACGTTGCGTGATTGGTCAGGGCGGCCACGAAGTC
CGTCTGATCCTCATCCGTGTCTTCGGATTTATTGCCGTGCTGACGTAATCTCCAATTCGAATGGAATTCATCGT
CCATCTTCTTCCTCTTTTTCGCAGTTTTCTGGGTTAATTGAACCACTGAGTAGTGTTCCAGATAGGATGGAGG
TTTGATTGTCTTTGTTTTGCTTCGTGTGTCCCGCTGGAACAGCATCCGGTCGTGTTTTTCCTGCCAATTAGCTG
GCCGAGGTTGAGCAATGGCCACAATATCTTCGAGGATCCAAGGATATTTAATGAAGAACGGTTCGCGACTG
AGCGGAAGGCTGGAAATGGACGGATAATCATTGTTAATCACCTGTTCCAATTGCGGTGTGAAATTCTGTTCC
GGAATATTCGTCCATACGAGACGATGTTTGACGCAGCCTTCACCATCTTTCATGTCGAATATGATAGGCTCCAT
CTGAAGAAACCTGGAAATTGTGATGTGATAACTGCTGACATTTTCGACTGCCCATAGCAAAGGAGTATATTG
ATTACGTTCGATACAATTTTTGATGTGGAAGAAATCGAAAAAAGCCTCTCCATATTCTCCTTCGGCGAATTGTC
TTCTGCCAACTTCTCGGCCGGCAGGTTGATGACGATGTGGATCGAATTTTTTAGCCGGAAGGCAAGACACG
AGTAGATGGATAGGACAGAGTTTGGCCCATTCTTCATCTTTCATTTGATTGGCATTTTTCAGAGCAACGAAAT
CGACGCCGTTGAGTTCGTACACCTCTTCCCACCTGTCCGATGCCATGTACTGGTCGAGGCAAAGACGTAGTG
AGCCCAGGGCGTAGTTGACAGCGCCCAGGTTGTCGTGGAGTACCACGACCCGGAAAGCGGGTGGTCCATT
TTTAGATAAATGCCGATTTAATTGCAGAGGGAATTCCTGATGTAGAAAATTCCGCAATTTAAGGGCCCAGTCG
GAACGAGGTTGAAGTAGGCCGTGAGATTCAGTAGAGAAATGGGCACAGAGGAAACAGAACCATGGCGTT
TTCTGCATAATGGCGGACATGGATCCTTCGCCCACCAATAGTTCCACGCAATACGAGCAGTAAACCCTGGAA
CAGGAGCCATTTTCGCATACTAAACAATCTCCAGTCGAACCACAAATGGCACATCCCACGTGAATATCGTCTT
CACCAACGCTGTAGCTTGTTCCAAGAAGTTCACTCTTGCATTCTTGATTGCAAAGACTTCCGAGAAATAGCG
GATGTTCCGTGCACGGTCCCATGATCTCCACATGACAGCCAAGGCAAAAATAATCAATGTCCAGCGTTCCGG
CTTTGATTCTGTCCACAACAGCAATGTCGCAATCTCGTGGACCATAAGTGACGTCATCATCTTCGGCATTGTTT
TTCTTCTTCTCCTTTTTGTGGCCACCCTGGAACGCAGACAAACGACTCTCTTTCTTCTCGTTGGAAATCCTCTT
TAATTTGTCGTTAGTGTCTTTGATTCGGCTGATGCACCTTTCCAGCCATTCAGGAAAGTCGTCCAGAACAACA
TCTCGATTAGTGCTGCCCCTTACGGACGAGAACAGTTTAATCGCCCACGATTTCAATTTGCCAGCTTTGGCAA
GTCTAGGCTGGTTTTTACTGTGGGCCAACACCTTCAGGGCTTCCAAAACACCTTTTTCATAGAGGTTGTTATA
GGCTTGATTAAGCAACCGAACAAAACGGTCGTCCAGGAAACCGCTAAGCTTTTCAGCCGGCATCTCAGAAA
CGGTTTGATGGCCGCCAAACCAGTACACCCAATAGTTGGTCGGTTTCTTGGGTGGAGGCAACCCACAATCAT
TGTGCGTAACTACAACACCAGGCCACCAAGGCCAGCCGTCCAGCTTAGCCCACACCATAGTCCCAAGACGA
ATAAACTTACGAGAAGCTAACGTTTTCTTTGGTTCGGCGCCATTTTCTGTCGCCTGTGATTCGCTTCTGCCGG
TTTTATTCGCCATTTTGACTACGAGCAACGACTTGGAAGAAAGTCGAATCGCCAACTGAAAGGCGATTCGTG
TGGCACATGTGCATACTTTGAACTAAATGCGTATTTAGTTTGAAAGAAAACTGCGTAGTTGGGATTTTGTCAC
CCCCTCCCTCTCTTCTGAAGCGTTCGAGAAAAGGCCGAAAACAGACGAGTCGAGAACATGTGGGTGAGTG
GGTGAATCATGGCGGGTGTGGGAAAGATAGCTGCGTACCTTCGTCGACTTAAACGACGCATTTCCAGATATC
CTGCAGCATTCTTTTTATTTTTAGTTGTCCTGTGTGGCAATTCAAAAACTACGTGACCGTCCAACGTTGATGCC
GGCCATGTTGCAGGCTTCGTCGTGGCACGTAGAAGACGATGGTTGTGAAACATGAGGTTCGACGTGACCTC
GTTAGAGCTGGCCAAATCTTGCCTTAGCAACGGGCCAATCACGAGCGATCTAGCAGTAGTGGTATGCACTAA
CGTCGTCTGCACACACACGTGTTTTGGAACAAACGTACAGTTTTGACGGAATAAAATGTTTCTTTCCCTATTC
AAACAACTAGATTCACACAACAAACGAGAATTCTTTCATCGGCTGGGGGACCTTTCACTCTTTTGGGCTTGC
AGAGATCACATCACATTTCTTTTTCTGGATCAGCTCAACTCCTTGGCGCTTTGTGACCTCGTCCATGGGGGAG

GTTTAGCCTAACTGTTTCTCGTGCTTGTAACGGTTGAAGTAACAAATAGCAGAGGA

>20 c8533_g1
GTCGATTTGGTGCATATATAAAACCAGCACGTCTGCAGTTAATCAGTTAAACGTGTCGAGTCTGATCCACCGT
TTGAACGTCGTCTGCTCAGCTCCTAAGCAATAGATAAAATGGAGTACAAGGGACTATCACTGTTAGTTGTGAT
GATTGTTGCCACTTTAATGGCAGATGCTTTACCGGCCGTATATCCAACAGCCGACGACATTGCAGCTTCCGAA
CTGGTTGACGAACTGGAGATAGCAGCTAGTAATGTCGAATACAATTACCGGGCTGCAGACCCCTACTGCAAT
GCTTGCTCCAGTCGTTGCCCATCACACTGTAGGCCTACAACTAAAACACCATACTATTATGGTCAGCCAACTA
AAGGGTATTACCCGACTACCTCGATGAAACCAACTAGACCGACGAAACCAACGAGACCGACGAAACCGCCA
ACGTGCACCGACTGCCCTCCGTGTAACCTTCCCCATCCGAATTTTATCGGAATTTGCCTGGACGTAATCCTCG
ACCTTGACCTTGATATTGGTAATAAATAAATCAACGAACACATCATCTTTAAAGGGTAATGAGAAAGAAGGG
CACATTATTTTAGGTTGTATTTTGGGGGAGGGGAGGGCAATTATTTTTGGTGGTGTTTAATTTAAAAGATACT
ATTTTCTTGTTGACCATTTTGCATTTTTGGGTGTGATTTTCCTTCTGCCTGAAACTGGAATAAATCAATTTGAT
TT

>21 c17941_g1
TTTTTTTTTTTTTTTTTTTTTTCAACGCGGTAACAGTTTATTGAATGAAAGAATAGAGTCATAATACCAAACAG
TACGTAACAGTCCCTGGGAAAGCTCATGAAAAACAAAGGTGAAACGAAACACTAATTTTAAATGGGAAACA
GAATTTGCAGTAACAACAACAGGTAAAATAAGCCAACAAACGATTATTTAACCATGATAACCACCATATCCAC
CAAAACGTCTATATCCTCCATAACCTCCGTATCCACCATAACCTCCATGTCCCCATCCGCCGTATCCTCCACGGC
CTCCATATCCCCATCCTCCGCGGCGATGTTCAGCGGTGTCCAAGTCGGCGGCCAGCACGTCGTCTTCAACCG
AACGACGTTTGCGACCCCATCCGTATCCACCATATCCTCCTCGGTATCCTCCACCATATCTTCCATATCCGCCGT
ATCCTCCGTACCATCCAGGACGTCCACCGTAGTATCCACCACGCCTATGGTGTTCGGCAGCGTCCAGGTCAGC
TACTTCGACTGCTACGTCCGACTCATCAACGGCGGCGGCCAGGGCCATAAGGCAAGCTAATACGATCACAAT
TTTCATGGTTGCAAAGTTTTC

>22 c17991_g3
ATTTTTTTTTTTTTTTTTTTTTTGCGTGCCTATGTAGTTTATTTGTGCTTATTATACTTGATGACAGTGTACGAATG
CGAACAAAGCATTAAGGATGATGTTGACATAGGCTGGCGCATTTAAAATGCTTTAGAAGGATGGGGATGGG
AAAAATTTGCGTACGGGACATCGATGACGTTTAACCTCGGTTGGCGGCGATCCAGTCCAAGAAGAAGGACA
CCTGGGTGTAGACACCAGGGTATCCAGCCAAGGCGCATCCCTGACCCCAGGAGACGATACCGTGTTGGACG
GCAGAGGCACCAGTGCCAGTGAAGAGGGGGCCGCCAGAGTCACCCTGGCAAGAGTCGATACCACCGTTG
CTGACGTCACCAGCGCACAACATGGATGGGTAAACTTCTGGGTTGGCAGCAGTTCCACCATAGAGACGGTT
GCAGGTGTCGTCATCGACGACTGGGACATCTACGCTGCGGAGTGTGTCGGAGATGACACCGCCGGAGCTA
GTGGTTCCCCATCCGGAAACGGTGACGATGGTTCCTGCAGGTGGGTCCAACTCGGATGTTGGGGGTGGCA
GAGCAATTGGCTGGGCAGAGGGAACGCTCAAGTCCAATGGGGCATCCAAGAAGAGCAGGGAGATGTCGT
TTTCGAAAGTCAATGGTTTGTAGTTCTCGTGGATGATGAAGCTGGCAACACCAC

>23 c20854_g1
CCTGGATTCGGAAAGCTTATAAAGCTAGTATTGTGATTGCACCGGATTGCACAAGGATTTGCCAAGAAAATC
TGCATTTCCACTGTATTTTGCACGAAACGGACGAAGATGACGATCCTCACGCTTTGAAAGACTGGATGCGCT
TTCAATCCTTCACAACATTGGACAATGGCGTTGCAAAAGGATAGGGAAAGTGCGTTGGAATCGTTGCAGTC
CATACGGTCGGCGACGGAAGAAGAAAATGCGACGTAGTGGCATCGTGAAAATTGAACAAGGAGAGTGCAA
ACCATAGGGCATGCCTCAAGAAAAACCATTAAGGTATCGAATCAGCACGGCATTGTTTGAAATGACATTCCAT

TTGCGTCTCCATCCATCACGTTCGACAAGAGGCTATGCCAATCGAACCGAAAATGGTAAACTTGCAGATTTTT
AATGAAACGCAGAACGCCAGCATCCCATTTTCCAAGAAACTGAATGAAAGCAAAGGTGGGTATCGTGTTTT
TAGATTCCAACAGCGGCAGTGATGTGAAAATAGAATGCAAATTGGCAGGCTCTATCCTGAATGAAACAGCAC
TCTGAACCAGTGAGCCACAATGGCAATGACTTTCATGTCGTCAACACGGAACAGCCCAATAGCAGCAGTAC
GGCTTTAGAAAACGCGCTTGGCAACGATCCCAGTGACCTGGAGTGGTTCAATACTGTGCCCCAGGAAAAAG
AAAAGAGGGGCGTTTTTCTCTTTGATGTATCGCTCACCACCTTCGAGAAAATATCTATAATCTTGTCAAGGTT
AGTGGATTCTCGAAACCCTACCACGACATGCTGGGGAAAAGGAATCAATTTAAACATCCCTTTGTGCTGCAA
AGCCACTGAACAAGTGGCGCTGTTTAACGAGACGAAAGGATTTAACTCGCTGCATTTCTGAATTTACGAAAT
ATCATGTGCAATGGTTTTGTTTTGCCCCTTGTGTGACGAAAAGATTGGCAACAATTGTTGCTTAAAAGCTATG
GACTCATCGGTGTTGCTGAAAGTTTAGCACATCGTCAAGCAGTGGTTTTGAGTGAGCTATAAAAGCTTTGAA
ATTGCGGAGTTGTTCTACACATTTCCTCAAACTTCGAGTGGCGAGTGTTCTTCAGTAAACTCTACAATGAAAA
GTCTAAGCTTCGCCTCGCTCTTTGTTGCCATTGCCTTGTGTCTGTTGAGTAACGAACCAGGCGTTAGTGGAA
CTGAATATGGGAACTGCAATATTGTGTGCCCGCTTGTGTTTGATCCTGTATGTGGAACAGACGGAATAACTTA
CGGCAACGCTTGCCTGTTAGCTGCTAAAAATGCGTGCGATGGGACCAACGTTAAGATAGCACACAAAGGAG
AATGCAAACCTTATTATTGAATAATGACATTTCCTTCTCTACAAATGAACGGTGCAGCTCAAATTTTTCAACTT
TCATGCCTTTGCTTTTCTGGTGTTTTCAAAGGAAAATACAGTTTAATATTTTAGGGGAAATAACTTTCATTAAT
GCAGCGATAGCGAGGAGAAAGCCTGCAGTACGAGAAATGGTTGCGTTGTTCACGGTATATACAGCTTCGCT
GGAACCATTTTGACTATTTTCGGTTTTCCAATCTTAGCTAGAAAAAATGTAGGTAAGCAATTTCGACACCGGAA
TAAAAATTGTTGCCTCAGTTTTTTGGTCAGAAATTATTTATAGCTTGTAAAGTGCTACCCAATTTACATTTGTAT
TTAGAGCATTTTGAAATGTCCAAGCTTCATGTGTGGTTTTCTTTAACGAAACTTGCAAGAATTGTATAATCAAT
AGTAGAAACCTCAATTAGAATAAGCAAATTATATGTTTGCTCGGCAGAATTTTGAACACAGCTTCCAAATAAC
AGGATTTACTGGAAAACAGTTGCTCATGTCAGAATCTCCCCTTATTAACGATGACCAGTTGGCCCTGAAGCT
GAACTATAAAAACCACCGGCCAACCAGTTAGTGCCACACATTCCATCAACGGCAAACGTTCTGCGTAAACGG
TTCCGAATTTGCCGAGAATTTTCCCACGTCGTTTCAATGGTACGTCTTTACATTTCATTTAGATCTTATGGCATC
AGTTACGATAAAAACTTTAAGCTTTCTTCTCGTGAGCTAGAAATCCTTAGGTATCGCTGTGATCGTTGGGCTA
GCATTGAGCTTTTCGGAAAGCCCGCGCGTCGTTCATGGAAGTATGTTTCTTGATTTTCCATTGTTTGCTATCC
ATCAAGTGAATCTTCAACTGTTATCGCAGAAAACTTGCACAGACATGGTGGGTGCGAGATCGAGTGCTTATT
GGACGACTATTTCCCATTATGTGGAACGGATGGCAAAACATACGCCAACGAATGCTTCTTGTTCCTGGAGAA
TAAGTGTGATAATGCCTCCGCTTCGAGAATCCGCAAAGCTTACGACGGAGTTTGTAAACTGGGTCAGCAAG
GCTGTGAAATTCCCTGCAATGGTCTCGGATACACCTTAAATCCATTATGCGGCACTGACGGAAAGACTTACG
ATAACGAATGCGAATTGGATGGCAGGAACAAGTAGGGCAAACAGAACAATCATTTTGAATCTAAATTTATCC
GGTAACTGTTGTTCACCAATTCGTGTAATTATTTGTTAGGTGTGATGGCACGGCGATTAAGAAAGCTTACGAT
GGAGAATGTAAACTTACTGCGCAAAGTCCAACCATTTCCCTGTAGGGGTGACATTATGTAATCCGTTATGTGG
GACAAATGGAAACTTGCATCAACACTTTGCCAGCCGATCGTTTAAATTGCGTGAATGACACCGTGGTACTCG
CCCTAGACTTACAACGGTGAATGCAAGACTCGCCTGGCTCCTCGCGTCAACATTATGGACTCAAAAAGGTTT
TAACCTGTTTTTCCTCTACGTCTGGACACACCAATTGAAAATTTTAAATTATGCAACTTTGTTTTGCTTTGTAA
AAACATGCGACAGTGTAAATAAATAAAGAGGGATTTCTTAGCTTGCAAAAAAAAAATGTTAATTGCTGCCCA
AAAGGAACTAAGAATTATGCAAAATTCGAATTAAGGTGGTTCAAGTTAGGGTACAAGAGATTTTAACTTCTA
GCTCGGTGAGTTTCGTGTTACCTTTGCAACGG

>24 c19171_g1
CTTCTTTACAAAGGCAGAATGTTCAGATCAGTCTTTCTTTTTACGTCGAGAGCCCTAGCCTGCGCATTAACCA
AACGCAGCTGCGTAGACAGAACAACTTACAGGGCAACGTCGTTACAGCAAGACTCTTTATCTCACGTCATCC
GAGTCTATTCTCCATCAATTTCAACTTTTCAACGTTTATTTTGTAGCAGAGAAGAAACCACGTCCGAAATGTC

TTTCAGAGATCGTAAAAGCGGGTCAGATTTCCGTGGAGGAGCTCGTGGAGGGGGTTCCAGTTTCGGAGGG
GGCTCTGGTTTTGGGGGCAATCGTGACTTCAAGAAGGCACAGCCCGGAGATAAGTTACGTAAGCCAAGAT
GGGAGATGGATCGTCTTCAACCATTCGAAAAGAATTTTTACAAGCCACACCCTAACTTGGCTGTGAAGTCGA
TTCACGAGGTTGAACAGTACAGGGCTAGTAAAGATATCACAGTTCGTGGTAGAGATGTCCCCTTCCCCATAA
CTTCCTTTGATGAAGCAAACTTCCCTGAATACGTCATGACTGAGATCAGACGTCAAGGTTTCAAAGAACCAA
CTTCTATTCAAGCTCAAGGCTGGCCCATTGCTCTTAGTGGATCAAACATGGTTGGAATTGCGCAAACAGGGT
CTGGAAAGACTTTAGCATACACTCTCCCTGCCATCGTGCACATTAACCATCAAGCTTACCTTGAACCTGGGGA
TGGTCCAATTGCCCTTATTTTAGCTCCTACCCGTGAACTGGCTCAACAAATCTCGTCCACTGCTAAGGACTTC
GGCTCGTCTTCGCGCATTCGCAACACTTGTGTTTTCGGTGGAGCTCCAAAGGGTCCCCAGTTACGCGATATC
GAACGTGGGGTGGAAATCATGATAGCAACTCCTGGTCGCCTTATTGATTTTCTCGAAGCTGGCAAGACTAAC
TTGCGTCGTTGTACGTATTTGGTTTTGGACGAAGCTGATCGGATGCTTGACATGGGTTTTGAGCCACAAATT
CGAAAAATTATTGAGCAAATTCGGCCTGATCGTCAAACACTCATGTGGTCTGCCACATGGCCGAAGGAGGT
GCGTCAGCTAGCTGAAGAATTTCTTACCGACTATATTCAAATCAACGTTGGATCCTTGACCCTCTCTGCCAAC
CACAATATTTTGCAAATTATTGACGTCTGCCAAGAGCATGAAAAGGAAACCAAACTGATGACTTTGCTGCAA
GAAATTGGTGCCGAAGATGAGAATAAGACAATTATTTTTGCTGAAACAAAAAGAAAAGTTGACAGCATCAC
AAGAGCCATGCGCCGTGATGGATGGCCGGCGATGTGCATTCATGGTGATAAGGCGCAACCTGAGCGTGATT
GGGTTTTGAACGAATTTCGCTCAGGCAAAGCACCCATTTTAGTAGCTACTGACGTGGCTGCTCGTGGTCTAG
ATGTGGACGACGTTAAATTCGTCATCAATTTCGACTATCCCAACTGTTCCGAAGATTACGTCCACCGGATTGG
TCGTACTGGCCGATCGCAACGCACAGGAACTGCCTACACGTTTTTCACACCAAACAACTCCAAGCAGGCCC
AGGATTTGGTGAACGTCCTGACAGAAGCAAACCAAGTTGTGAATCCCAAACTTTATGAGCTTGCATCTTCAA
ATCGTGGTGGTGGAGGCCGTTCCAGATGGGGTGGCGGCGGTTATGGTGGACGTGGAGGTTATGGGGGAG
GTAACAGAGGCTATGGAGGCGGACAATCTTACAACTCCAGCCGTTCCTGGGTTGAACGCTAAGAACTCGAC
GAGTTTAGCACAGATATTCAAAGCACCATCAACTAGTTCTTTTTTTTGCAACCAACATTTAATTATTCCAATTT
AATGAGCTGAATGAGGGTCTCAAGGCGTTTTCATGAAAATATCGAGCATCTAACGCTTCGTTCCCCACCACG
CTTCATCTTTTCGTTTTTGTAGGCCAATTTTTTCGACGTCTTTTCAACTTTTGGCATACTACACCAGCCATTTCA
CTGACACGTTACAATGTACTCTTATGTTTGTTCGAGTTAACCGGAATACAAAGTAATCGTAATAAAAAAAAAA
AAAAAAAAAATTA

>25 c20224_g1
CGGGAGTGTTTGATACAGCCACTGTTGAAAAGCAATACGTTTATTTCAAGCATGAAGTTAATACTAATTATCG
CTCTCTTTGTAGTTAACGTTACGGCTTCTGATTACACTAAGAAAAATCAGTATGATTCAAGATACGCCGTACAA
CCATCTTATCCCTCACAATCGGTAGATCTTTTGACAGCTATCAAAAAGAATGGCTTCACAACGTTCGTCGATCT
GATCGTAAAGGCTGGTTTAGAAGAAACCTTTAATGCCAACAGACCTTTCGTCGTTTTGGCCCCCACCAACGA
AGCCTTCGCAGCCATCGATCCTGCAATTCTAGCAGCCCTTTTAAAAGATATTTACCTGTTGAGAGATATTCTTC
GTTATCATCTGGTGCTTTTCAGGCAGCCATGCAGCCTCACGTCTTCCATCTATGTTGAATTACCTATAAACACT
GCGCTAGGTGAAGTAGTCCGTTTCAACGTGTACCGGAACAAAGGGCCTTCTTTTGCTACCGAAGATATTGTA
ACAGCTAATGGGGTACCACTCTTAAGAGGTATCCCAACCGGTAAAAGCATTATTTACCCCATTGAAAAGGTTC
TCAATCCGCAAGACGTATCGGCCAACAACACCGTCATCAATTTCTTGAGAAGCAACAAAGACTTCTCCATCTT
CCTAAGCGTATTCGAAGTCCTTGGCTTAGCTGAAAATAATTTCAAAGCCCGTCCGAAAACGACGTTTGTTCC
GACCAACGCCGCGTTTAAAGCGCTTCCGCCTGGTGTTCTTGATTCCATTTTTGCCGACCCAGACAAATTGATT
GTGCTTTTGAATACGCACATCGCGTCCGGAACTTTCTACACAGCCGGTCTCATTGACGGCCCCCTCACTGTTT
TAGCTGGCATCCAGGTGGATATCAACGTAACTCCCTATGACATTATCGTGGGCAATGCTGAAATCATTGAGGC
GGATATAACCGTGTTTGAAGGAGTTGTACACGTTATCAGCTCGGTTATCCAAGCCGAAGAAACTTGCTTCAA
CCGGCTGTATTGATGGCGCATACTACAAATTATTGGCAATGGAACTGAAACATGGGATTTTTTTGAAGAAATA

TATAAAATACGAATATTTAGCAAAAAAAAAAAAAAA

>26 c22933_g1

AAAATGGACGGATTCACGTTCTCGTCGTAATCCCGGCTGAAACCGTGCTGAAGTGGCAATAATCCGAGTTGC
TGCATTTTCGCTCGACCAATAACAATTGGCAAAAACTCGTTGTAAGTGATGTGCTGCATTTGGGCAATTAAAA
TGCGTCTGGCCTCTTGATAGAGACGCTCATCATCCCAGTGTGGATTCAACGTGGCCAGCAAATCCGCAACTC
CGTTGTGCTCCCTCAAAAATACAGTTTGCGAGGCAACCATGTACGGAGTGACGTTAATTCGGTTGTCACCTG
CTCTGAAGCATTTGACTTCTTCTGGTGGATCAATTCCGGTCACGGCTCTGGACAAGGCACAATCGACATCGG
TGTCATTGTCGGGTGGATGCAAATCCATGCCATGATGATAGCCATGTCGGGGACGTCGCAACACTTTCAACG
TCCCTTTTTCAAATGTTCGCAATTCAGCAGCGACTTTGTCATCGGATCCATACAATCCGGAAAGATCCAGGAA
ATGCGTGTTTTGGTTCATCTGTTCTACGTGACCGAGCCGGCCGTCAGTTCTGCATGACGGGGCAGACCTGGC
AAATTGCATGCAACGTTGGCGAAATTTGGAATAAAATGGATCGTCCTTGGGAATTTCAATCGGAATGCATTT
GCCATGACTGAGATCTTCTTCCGAAAGAAAATTGCCTTCGTCCGTGCAACAGGGAACTGGTAATCCATCAGC
TGATTTTGATTCCATGGTTTCCAACATATCGTGGTCAATAAACTGTCCATACTGCATCACAAAAGTGCTGTCTG
AATCGCTGGGCACGTCGATATCGCGAATAACGGACAGAGACAGCAAACGCGGAGGAGGGAGTTGGCTGCC
ATCTTTAGCCAATCGCGGCATCCATACACCGTCGGCGTAAGCTGGAGCAAGTGCTCGTTGATATTGGGTTTTT
GGCAGACCCCACGGAGTATGTCCGGGAGGATGGGCATTATTGCACGAACCGTTCATCGTCCGAAAGGGAG
AAGCCAACGTCCTTTCATCGCAATTAGGATGAATCGGGCACGTATCAAATAGAATAGTGTCTCTCAGACGGA
ACCGGTTTGCCACACACTCGATTTGCTCTGGTTTAATTCGGTACCTTCTGGATAGTTGCATAACAGCGAAAAC
ACCGTACAGCGAACTATTGCTGATTTTCACCGAATCGGCTGTAGAGTGAAAGAAGACCCAGCTGTGCGTGA
AACTGCCCGATCCTTTTCTCACGACGATATTCTTCTGCGTTAAACGCTTTTCGGTTGCAATCATCGATTCCATT
TGCGATTGGCCGGCCAGGGCAGCTTCGTTGACAGCCACCAAATCGATATGTTCCACGTGAGGATCCAACGC
GATGCCTTCGTCGCATGGGTTCGGCTTCTTAAACGGCGCTTGATAACGACCGTTGATGGGAATATCTGGACA
ACAGAGTCCATGGCCTTTTCCGTTTCCTAAAATGCAGGCAGTTCCAGGTGTGGCCCGAACAGCATCGTACCA
TACGGCGCAATCTTTAGCCGGCCGGCATCGTGACTCTAAACCACCGATGTTTGGACAATACTTTTTCTGCGG
GGTAGGATATTCCACTGGCACAGGTTTGTAACTTGAAGCGTAGGGGTTGTGATAGTTGGGTTTAACACCTGT
GGCAGGTGGATAATAGTCAGGTGTAATGGGCCGTTCAGGATACTGTTTAAGCGGTGGATGGTCCGGATAAT
GACTGGGACGTTTTTGGCTGCTGGCCAGAGCAAACAATAGTTCCATTACGAGCACTGCAATCAACCACCGC
AAAACCATTTTCCCGAAATGATTTTTGTTGCACACGACGAACTTCACTTTCACTGATTTAAATAAAGATTTAAC
CGGAACAATTGACCTGAAACATTAAAAAGAAAAAAATGTTTCAGTGAGGAAAATCTGGTAGAATAGTCTCA
ATTGTTCCAGCGTCGTATAAAGCACAGTGAGCACTGCGGTGCACAGCTTTCTTCTCTCCTCGTATTTATAGTG
CGCGATTCCAGCTCCACGTACTTCCCCAAAGTGGGCAATGATTTTCGGTTTTTTTTTTCTGCTTCTATCTGGAC
GTTCACAAAGTCCGGAATCGTGTTAATCAAGGTTATGCAACTATGAAGTAGGTTTTCTTTTTACTGTTCCAATA
ATAGAATGCCTTCTTGCTCTAGTGATTTTCTAAGGGGTGAAAGCGATTAAGAGCAAGCCGTCACAAATGGTT
ATTCATGCCAAACCGTCGGAATTGGATGGTGAATTGAAATCGAATATAATAACAGGTGGTTGGTTGCCAAAC
AGGGCGTGAACGTCTGCACTCGTGGTCGACAAAGAGAAACTGTAACAACTGAAAATAAGGAAAAACCCCG
GGGGGAAGAATCTTGCCGGACCGTTTTGTCGTTTAGCTAAATTGGAGTCGTAAAGGAGAAAGACATTAGTT
TTAAAATTGATAAAAAAATTATTCAATCAGGTGAAAGGAGGACTGCACATTGGAGGAAATGAAAACAGGTC
GTCTGTGGTAGTAGCATATGCTGTCACGTGACACTTCCATATACCACACACGTCGAACCGTTGTTTTCAATTTG
CAAGTGTCAATTTCAATGCACAGTTCTGGTTGAGCTGTTTTGGGCCACAGAGTCTACCGGAAAGAGGAAAC
TGTCCAGCAATTAAGACCACCAGCCTCAATGCACAGTTAACATTTGGCCATCAAATAAGATTTCAAACACAAA
ATTACAATGGCGAATTGATGTGTCAACAATAAAAACATGTTGTGTATGCAATTTTCAGGAAATCAAAACCCAC
AATCGGATAACGATTTCAAGAATGCAGTTATATAATTTGGGCTCGTAACGGTCCAGCTTTAGAAATTGCGTTC
AGTCGAGCTCGCCTGCAAACTTATTAGCAAAATTAAGAATCTGGGGAATTAGATAGCAAGAAAAGAAAATG

AATTCTAAAAAAATTTCCCCGGAAGGAGACCACATAGTCCGCCATTGTGCCGTGTACGTTGTAGCCATTTGG
GTATTGCCTTTAGGGTACACCAATTGCCATTGGCCCAAAAGGCATTACATCCTACAGTATCGATGCACTTCCTG
GCACAGTTTTCGGCTGGACCGAGGTTTTCAATATACACAATATCGAAACCGACGTAATCACAATCCGAAAAC
CATTTGACCTCGTATTCGCCAATATTCCAGGACGTCGGATCGGCCTCTTTCCACGGAAGCAGACTCATACGAG
GAATGGAGGCCAGACAATTGCAATCCACAATGGGATTCGTATCAGAAACTGATTTAAAGATGAGTGGTTGA
GTTGACAGGACGTGACTGTTGTCACAGACCAAACGAGCATAGCTTGTCTTGCGGATTTCATTCAGTTGATCT
TCTGTGAATGAGCCGGCCTGCCCAGCCAGATCGTAAAAGTAACGATCACCGCGTTTCAACCTGGCAAACTG
GTCGGCAATGATGCACTGGAACGTTGGGCCCACCAAGGCTCCTGGAGCTGGACGCTCCGAAACGCCTGCA
ATGTACAAATCGATGTCGTCCACACTGTCGTAGAGCAACTCGAATTTCTCAACAACCTTTGGAGGGAGAACA
TCGAGAAGGTCACTGAAATGTTTAGCTCGAGGTAGGCCACAAAGAACACGGTATTCATTATAACCGGGCAA
ACCTTGATCACGACCACGTTGAATATTGAGGGACACCAGATCCATGCCGAAACCTTTCCCTTCCTCTTCAAAC
AAGTGATTGGTGACCTCTTCAGTAAAGACGTTATCCACTGTTTGGCCGGGGATAGTTGCCATGGCTATGAGT
AGCTTATCGATGTTTCCAGGTGGATATGCCTCTTGTGTTTTGAAAAAATGTTGACGAAGAAGTAAATCTTTTT
CGTTCACTCTTTCTCGGTTGATGAGACTTTGTTTTCCTGGAACGAGCGAATGGCCGTAACGAAAAGCGGCC
GCAGCAAATTCGTTCAAAATGGACGGATTCACTCGCGCATCGTAATCCGTGCTGAAACCGTATTGGAGTGGA
AGTAACCCAAGTTGTTCCATTTTCTCTCGACCGACGAGAACGGGCACGTATTCGTTGTAGATTATATGATGCC
ATTGAGCAATTAAGATGCGTCTCGTCTCCTGATAAACTCTCTCGTCATCCCAGTGAGCGTTCATGGCAGCAAG
CTGTTCTGCAACTTGGTTGTGTTGGCGAGAAATGACAGTTTGTGAAGCAACCATATATGGGGTGACGTTAAT
TCGATTATCTCCTGCTTTAAAGCACCTGACTTCTGGCGGTGGATCTATTCCGGTAACGGCTTTGGACAGCGCA
CACGTAGAAATCTCTGGACCAACGTCGTCGGGAGGTGGCAAATCCATGATGCAATGCTTACCCTTTCGTTTA
ACTCGAGTTACATTTAAAGTTCCATCTTTAAATGTCCTTAAGTCACGAGCTAATCCTTCGTCTGAACCATACAC
AGCCGAAAGATCCAAGAAATGTGTGTTTTGATTCATCTGATCTACATATCCTAATCGACCATTTGATCTGCACG
CGGGAGCCGAACGTGCAAATTCCATGCAACGTTGACCGAAATTGGAATAAAATGGGTCATCTTCAGGAATT
ACTACCGGAATACATTTGCCGTGACTGAGATTTTCTTCCGAAAGAAAGTCTCCTCCATCCGTGCAACACGGG
ACTGCCGATCCATCCACCAACTTTGATTCAACAGTTTCCAACATATCGTGATCAACAAACTGTCCAAATTGCAT
AACCCAGGTAGTATCGCTTTCGCTGGGCACGTAGACATCGCGAATCAAGGAAAGAGATAACACGCGCGGTG
GAGGTAATTGGCCCCCATTTTGAGCCCGACGAGGTTCCCACACACCATCTGCATACTCCGGTGCAAGAGCAC
GTTGATACCTGGTATTCGGCACACCCCATGGAACACTTCCAGGAGGATGAACATTATTACATACTCCAGTTAT
CGTCCGATAAGGTGACGTCACCATTTCTTGATTACATTCGGGATAAACAGGACACTCGTTGTAAATGCCTGCG
TCCTTGACA

>27 c23017_g1
TCCTCTCAGGAGGCATACTATTATAAATCGATTAAGCTGCATTCTGTTTTCAACGTGAATTAACCCGTTATTTAA
CGAAAAACCAACGTTTAATGTCACCAATCGGGGTTATCGGGATGTGGTTGTCGTACAGCCAAGTAAATATAA
AGGAAGCGTTTACATATTTTCTTTAGACTGTTTTGTTCTGTGTTCGCGTGGAATGCTTTACCAAACTCAACTGT
GATATCCATTGTTTGCCAAGTGAAGTTTCGATTTTCTGTCTAACTGGCAACTACAATAGGAGATGCTGAGTGA
TGTCCCAAGACGAAGAGGTCAACAAAAAAGTTTTCAACGTCAACGTTCACACGAAATAGCACGAGTTCACG
AGGAAAAACAGGAACTCTGCTATTAAATTCAGATGTGAACAGTAACCAATTGGTGCGTGAAGAGAGACAGT
TGCTCTACGGTCGTCAGTTACGAGCTTCTGCCTTTAACGTATTTTCACTTAGTCTAACGTTCGTTTTAATTCCAT
AATTGCATTGATGAAATTTTATCATTTGCAATCTAGTTTTCACCATGCCTATCAGCTACAAAAACCAAATCAGG
GAACTATTCGTATTCTGGAGTCTGCAACGATGTTGTTCGTTGGGTGTCGGAGCATTTCCAAATGGAGTGAGT
TTCTTTGTCTTTTCTGCTAGTAACGAGGATCGAGTCAAATTCGGATTAGTTCCATCACTCATCAGACAAATACA
GAAACAAGTCGTTTTCCTTTGTCATCACACCAGCACTTGCAATGCTCATTGATTTCTCATTTCATCTTGGAGTC
GAGGATTATCACCTTTTACAACCGTGGCCGGAAATGGAAAGCAACCTTATGGCTTGCATTAGACCTTTCTCTG

CGACAGTATGTCCCATTTCGCTGATTAAATAAATCATATTAATTACCTGTTCATTTGATTAGGTTTGGTTACTATT
CGTCGTTTCGACAGTCGCACTGATCATTTGCATGGCTTTTTTGACGCATTTCAATCATCAATACATCTTCAAAT
TGACTAGTTTCATGCAAACAATGAGCGATCACGCGATTTACGCAATCACCGTCATCACTGGACAAGGTAAATT
CGTCTTTATATGCCTCAGCATCAGGAGAAATGATTTATCTTCTTCTTTGAAAGGCAATTCTGTTTCCGCGAAAG
AAAGACTTTCATTCCGTCTCTTGTTAGGAATGTGGTGTTTGACCATGGTGGTCCTGGTAAACGCTTACACAAC
AACTTTGACGTCTTACTTGACTGTGCCAAAACTGAAGCCGATCGTGAACACGCTGGCCGAATTGGCAGCCA
GTCACGATACACAGATGACTGTGGATTTTGAACACTTTAATTTGGAAATCGTGATAATTCAGGAGGCCACATC
TGGACCAAAGAAAATCATTGGCGATTCGCTTCGTGACCATCCAGAGTTCCTTGTAAAAGGCAGCAATCTGGA
GGGCTTAAAAAACGTACTGGAAGCAGGAGCTACGTATTTTACGGTGAGTGAAAGTTTATTATCGTAATAGGT
TTTGATTGGTTTACTTTCCTTTTTTAAGAATCGTTTTATCGTCAAATACTTTATGGCCATCGATATAAAAAGCCA
CACCAATTGCCGAATGACCATGTCGGATCCAATTCCCTTTATCGAATACTATTCTTTGGGTTTACCTAAAGGTG
GTCGACACAACTGGATCATCAATCACGAGTATGGATCGAGAATCAAAAGTTTGTTATTTGTTGTTATTTCTAAC
AATAATTAACTGTATTTGATGTTTGATAGATTGCAGTATTTGTGGGAAAATGGACTACTTGCCTTCTGGTTTAA
GCAGTACACACCAAACGTTGACAAGTGTATGGTAGCTAAAGCTGAATTGCGCAAGGAAAGATCTGCTTTGA
CGCTGTTAGATCTCTCGAGCGCATTTGCGCTACTGGGCATCGGAATTGGCTCGTCGTTGTTAGTGTTTCTTTT
TGAAATCATGACACATTTGAAACGAATTCGAAAATTCCGCCGAGCCACGAATGCAATTATTGTTCAAAATCGA
TGGGATGGTGTTTGAATGTTTTGAAATTAATCTTCAACCCAATAGTCGCAATCAATGTGTTGCTAGTTCACGA
ACAAGTGTTGAGCCAATTTTAAATTGAGTCTCTTGTAGACGCACAACAGCCTCGTCCGATATTATGAAACAAA
ATGATACAGTTACGAACATTGCTCTGTCAATTTCATGTCACCTATGTTAAATGTATAATTAAGTATTTATCCAAAC
CAATAATATAAAAATACTTGTAGCACGTGATCTACACTGCACTTTGGCTTTTAAATTTAAAAAATTTAGTATGA
GATCTTCGTGTATGGGGTGTCGTAAGGCCGTTTGAAATATTTGTCGTTACCTTCTTGCCACGCAAGAAGATTT
ACACGGGGAATAGACGTCGATTCGCAGCCCACAACTGGATTTTGGACAGACTCCAATTTGAAAACCAATGG
TTGAGTGTGAAGGACGTGGCTGTTGTCGCAAATTAGACGGGCAAAACTTGTCTGACGGATTTCGTACAATT
GCTCTTCAGTGAATGAGCTAGGCTGTCCAGCAAGATCGTAGAAATAACGATCACCTCGTTTTAGTCTTAAGA
ACTGATCGGCTATCGTGCACTGGAACGTAGGGCCAACAGTAGCGCCTTCAACTTTGGTCTCGGAAATGCCTC
CGATAAACAGATCAATATCATCCACCGAGTCGTAGAGGAGTTCGAATCGTTCGACAATCGCTGGGGAAATCA
CATCGAGGAGATCCTTAAAATCTTTTGCACGACGGAGTCCGCACAACACACGGAAAGTATTGTATCCGGGA
ATGCCGCGATCACGACCGCGTTGGATATTAATAGATACCAAATCCAAACCGAAGCCTTTGCCTTCCTCTTCGA
ACAAATGATTAGTAACCTCTTCAGTGAAATAGTTGTCGAAATCTTGCCTGGGTTGTGTGGCCAATCCTGTAAG
AAATTTGTCGAGATTGCCAGGTATGTAAAGGGTTTGCGTTTTCAAGAAGTGCTGACGTAACAAAATGCGAG
ATTCCCTCTTTCTCCTGAAATTGATTAAATCTTGTTTTCCTTGAATAAGCGAATGACCAAAACGGAAGCTTGC
AGCCGAAAATTCATTTAGAATGCCTGGATTCACGTTGGCGTCGTAATCGTTGCTGAAACCGTGTTGCAATGG
CAACAGACCAAGTTCTTGCATTTTCTCTCTTCCAACAACACCTGGCAGCCATTCATTGTAAGTGATGTGCTGC
ATTTGAGCAATCAAAATGCGTCTAGCTTCTTGATACAGTCGCTCATCATCCCAATGCGGATTCAAATAGGAAA
GCTCAGTCGCAAGTCGGTTGTGTTCACGCAAGAAAATCGTATGCGTTGCTCCCAAATTGGGACTCTGATTCG
GTCGGATATCGCCTGCTTTGAAACATCGGACTTCAGGTGGTGGAGGAATGCCGGAAACAGCTTTGGACAAG
GCGCAATTTAGATCGGTAGTATTATCAGCTGGAAGTAAATCTAACTCGTGATGTCCCTTTCGTCGAGTTACTTT
CAATTGGCCTCTTTCGAACGTCCGTAATGTTGTGGCTGTGTTTACGTCAGAGCCGTACACGTTTGATTGATCC
AGAAAGTGAGTGTTGCTATTCATCTGTTCTGCGGGGCCTAGGCTGCAATCGGATCTGGGTGCGGGAAGGGA
TCGCACAAAGTTCATGCAACGCTGACCGAATTTCGCGTAAAACGGATCGTCGATTGGAATCTCAATGGGCAG
ACATTGCGGATGAATAAGCTCCGTGTTGGTCAAGAACTTGCCTTCTTCCGTGCAGCACTGAATATCCGTGCCA
GCGTTCGTCCGGAAGACAGAAGTAAGGGCCAAATCATGGGTGACAAATTGTCCGAATTGCATAACCCAATG
GGTATCCACTTGGCTTGGGGCATCTTTGTCGAGGACGACGGATATAGAGACTAATCGGGGACTAGGAAGGG
GACCACCTTTCTGCGATACCCTAGGCTGCCAAATACCATCAGCATAAGCTGGGACGAGGGCGCGTTCAAATT

GGGTCCTCGCCTTTCCCCAGGAAGGATACTTAATGTTATTACAGGAGCCATCAATCGTCCGGAAAGGTGAAC
GAAGAGTTTTGCGATCGCAGATGGGATCTGCAGGACACGTTTTGGACAAAATTGTATCTCGCAGTTTGAATT
GGCTCAATCCATACGCTGTCTGCTCCGGAGTCAATTGAAATCTTTTGCCGAGTTCTCTGGTAGTTTCAATAGC
AATTAAAGCTCCACGACTGAGTCGTTGTGCTTCGTTTGTTGTTTGAAAGAATTTCAAATGATTGTATGCGGAT
GAATTGGGTCTCACTGTAACATTGTGGATGCGACGAATTCGCTCGGTTTCATCCATTAACTTCAATTGGAACT
CTCCTGCATGGGCAGCTGCGTTTACAGAGTAAATATCGATACACTGACGTTCCACTTTTACGATTTTCTTGGTC
TCTTGCTTAACTGATCCTCTATTGTGACCATTGTGGGGAATTTCCGGACAGCAGATCCCTGGATGTCCATCTG
ATAATTTGCAGGATGTTCCAGGATAAGTCAGCACAAGATCGTACCAGATGGCACAGTCTTTGGCATGTCGAC
ACTTGGATTCGTAACCTCCAACTTTCGGGCAATAAAGCGAATGCTTGTCGCCGAATTTATCTGGAACATTAGG
ATAAGGAGGTTGTCTTTTAGGATACGTTTCTGGAACCGGTGCAGGTTTCCAATCTGGCTGTGAGTTGACGG
GATAATTGTTTGGATAAATTGGTTTGCGGATGGGACTGTAAGGGCTGGATGAACTCGTTGGCCGATGCGGTT
GAGCGTAAGGACTGTGATAGCTTTCTTTTAACTCAGGATATGACTTTGTGGAGACATAAGGATCTGGTTTGA
ACGCTCCAGATTGCTCGTCGATCACGTCGAAAAAGAGCGGAGGCTTCTTGATCTCAAATCCGAGGCCTGAA
GGATCGTACTCAACGAAGTCGTCTTTGAATGGTTCTTGTTCGAATAGATGGATGGGTTTTTCGTCTGATGATG
GGCGTCCCCATGATTCAAATTGACGTCGAACACGTTCGCTGCTGTTGGTAACTTCTTTTGGTGCCTCTTTGTT
GTCGCTAGCCATCACAGCAGCGATTAGACACGTTAAGGCGGATATGATGATCCATCGCGACATGATCTTCGCT
CCCATAAAATTGTCTTTGGTGGTAGCGGATTTAAACTTGTCCGTTGCAATCTCCTTTCTTTAGAATTGGCGGTA
ACACTAGTTACCTACAAAACGATTAGCAAGATGTGTGCGTGCAGTTAACACGACGCGTTTCCACTATTAAATG
AGCTCTGCTGTGAACAGGGATCTACCTTTTGTATTTATACCTGCCTCCAATACGCAGAGAGACTTTTGCACAG
CACCGGAGGTCTAGAAGGGGGTGGGCAGTCACGAAACGAATGGAATAAGAAGAGAGAATAAAAGGGGA
AAAAAAAGACGAGATTCTATTTCCCTTTCTCGGAATAGAATAGATACTGACGAGTGTTTTCGTGATTTTACTT
TATATGACATAGTCAAGAAAACCGCCATGTGGACAGATGTCGGCCCTCTGAAATCCGGAACGGAAACCGTAT
CCGATAGGCCGTGAAAAAGGAAACCTAGTTATCGTTATAGGCCATACCTTCTTTCTCCTCATCAGTTCCGAATT
TTTCACCTGTAGTACATCGTGCGGTGAGTGACGTATGCCAAAATAAGTCACAGCTGACGGACGAAAAGTGC
ACTTTGGAAATGTACACATAATCAAATAGGAAGTTAACTAAACTTTATTTTTGTAAAAGAATGTCTTTTACTTT
CAATTGATGGTGACACAGTTAAATTAATCGAATGCATTCACTACGTCTCGGGACGAACTGAGCGTTGTCATTT
GCCA

>28 c19046_g1
GAGCCGTAAGTTGCAGCCTTCGCCTTTGGTGCAACCACGTAGCTAGATCCTTTTCCGTAAGAGTCAGCTCCA
TAGGTTGAGCCTTTGCCATACGATGTAGTGCCATAGTTGGTTCCTTTGCCATAAGAATCAGTCTCGTAGGTTG
AATCTTTTGCCTTTGGGGGAACGCCATAAGTGCCATAGTCGGGTCCAGAGCCGTAAGAGTCAGCTCCGTAG
GTCGAAGCTTTCGCCTTTGAAGGAACTCCGTAGTTGGATTTTGGGCTGTAAGAGTCATCCCCATACGAGGAT
CCTTTTCCATTTGATGGAGGAGCGTAGCTGGATCCAGGGCCATAAGAGTCAGACCCGTAAGTCATATCTTTC
GCCTTTTGTGAAGTGCCATAGTTGATTCTAGTGCCGTAAGAGCCATCCCCATAGATTGAATCTTTGCCATTTG
ATGGAATGTCATAAGTTGGTCCAGCGTTGTAAGGTTGGTACCCATCACCGCTGTATGGCTGTTTATTTCCCTT
GTTGTTGTCGTAAGGCGGATACCCATCGCTACTATATCCATCAGCCAAAGTAGCGGCCACGAAACAGGCGAA
AACGAATACCTTAGTGAGATTGAGCATGTTGCGCACTGCTGATTGGTGCTAGAGCAACTGATGAAAATGTTT
GGAACTGGACTCGTTTTATTTAGGAAGGAGCATAGGGAACGATTTGGCCGGCCTGTGAGGCACTCAACGAC
GTCGTTTTGGTGTGACTAGCACGTAAACCTTGGCCTCGGTTGAACCACCTTTTTTGTTCCATCGAGTTGCAA
CAAATCTTCGTTCCACAGGCCAAGGCTGCATGAATTAACATGATTGCGACACCAGTTTCACGAGCACCACTC
CCTATATTCTTCCTATAAATACCTGCTGATTTCCATCGTACTTTCGTCATTCACCTTTTGGAGCACACTGAAATG
GCATGAGTAGCTGTATAAGTTCGTTTTTGCTGAATTCACTTTCATTTCTTGATCGTATTAAAAATTTCTCTTACG
GCTTTGTTGCACACTTTCTAACGCAGTCGTCATGCCCAGCGGTGGATTGAGAAGATGCGGATAACCAAAGA

GGGGACACACTGATGATGGATCCCCTAAACGATGGATGCAATAGAACAGTGGATAACCTATGGGCCTAGTG
GGGTGGATACAAAGGTTACCAAAAGCTACGCTTGCGTTACGGGGACGACTGGACCAGACCTCTTAAGAGA
CCTCTTCGAGCCACCACTGTAAAACAAATGAACGATAGCTGACCCCTCCTTCGAAATTGCATTAAAAGTCTCC
TCCGGCTATGCGTCGTCTCAAAACAAATGAACGATAGCTGACCCCTCCTTCGAAATTGCATTAAAAGTCTCCT
CCGGCTATGCGTCGTCTC

>29 c16960_g1
GCGATGACTGCGAATGCGGAGAAGACTCCTCGTCCTCATCCACTAAACTCCACATCCACGGTGAACAACATG
AATTTCTCGACATTCGCAGTCATCGCTGTCCTGGTTTGCATGGCATTGTCTATTGATGCCCAGAGACCTGCAA
AACCAAGGCCCGCTAAGCCTACTATTTCTCTTCAGCCATCTCTCGCTGCAGCACTTCGTTCATTGCCATCACGT
TCATCACCTAGTAGCAATGTCAACATCAATACCAACGAGAACAATAACGAAAACTTCATCTTCGACTCACTTTT
TTCTGAGCTGATCAGCAATCAAAACAACCAGGATTCAGATCCATGTGACGAAGACGACGATCTCTGCTTTTG
GAGAACGATAGGAGCTGGCCGTCTTGCGAAATTACCAAAATCCATTGTAGAAAAGCAGGCAGGTGTTGCTG
CTTCTGACGATGACGCTGAGAGTGTGAAGGTAGAAAAGAGCTCGGATGATATGGGACGCAGTCGCCATCCA
GTTCGGGGTTCTAGCAATTTGAACTTCAATTCCAACAGCAACGCAAACTCCAACGATGATTTCATCTCCCTAT
TCGCATCTCTCCAGCAGTTTCTTAAAAACCAAAGGAAACTCACCAAGAAAACCTCTGTTGCAGCTTAACTAA
TAGCTTATTACCTATTGGCTACCATCTCCTCTGGCACCGACTATAAGAACAAACACTTTCCTGCAAAGTTTTCT
TTTTATGAAATTGTCATAGATTTCCTGGTTCCATATGTTTGGTATGGGTATGATTCTGTTATGGTTGTGGAATCT
AAAGACAAATATATCCAGATTATATGAAAAAAAAAAAAAAAAAAAA

>30 c3162_g1
TTTTTTTTTTTTTTTAATGCTTGAAACATGTAATCCCGGATAACAATTACTCAAGGGCACATTAATTGCCCTCTA
AAATAGATGAACAGTCGTGTGACTTTACTGACAAATGCTGGTGGAACAATCGCGTTTTCCAGCGACGGAGG
CGATTATTCCATCACTGTGCTGGTAAAACATTGGCAGCAAATTGACCTTCGTAATTTCCGGGTGGGTAATAGT
TGTAGACGACATAAGTTGTGCCACTGCTGCTCTTAGCCTTTCCGACACCCACCATCTTTGTGGCCTTCCAAAC
GACTTGGGTAAAGTGACCTGTCGCCATAGAGAAACCAGGTGAATTAAAATCATAATCTTTGATTTCGTTGTAA
AATGCAGTCACAGGATCTCTTCCGTTCGGTTCCCTTCCCCAGGAGGCATAAAGATTCTCTCCAAAGCCATTAT
TGCTGTGAACCAACGTGTCTTTCTGTGCGATTGTGTCGGCATAAGCCTGCGCCACTTTGGTGAGTCTAGGGC
AGATGAATAGCGCTGGAGCACCGTGTTTCTGCCGGTATTGGTTATGGGCGTTTAATGATTCACGAGCGAAAG
TACTGGACTGACGATCCGTCTCTTCAAAGGCAATTTTATTTAATTCCTCCTCGGTCTTGCCAGCAAGTTCCCTA
GCGTAATCATCAATTGGATTGTCGGCTTTCACCCAGCAAACGGTACAGAAGGCCAATGCCAAAAATAGCAGA
GTTTCGAAACGCATTTTGCCCTTGGATATCAATGCTGGATCTAATGATGCGAGTCAAGC

SF vs PF down-regulated genes

>1 c22798_g1
TTTTTTTTTCAGGAAAGTAAATATTGTATTTCCCAAATAATACATAAGACCATCCATAAGATTAAGAGCTGATTA
GAGAAGAATGTAAGACTTTAGGAGTCTATACAACAACAGAAACAGGAGATTCACCACCTTTGTAAGACCATA
TGTTTACTTTAATGATGCAATACCCACTACTTATATATTTTCTTCATAATCCAAGTTATACAAATTAACCATGTGC
GTGAGAGCTGTTTGACAAGGAGTAAAACTTGACCGTTTCGAACGAATTTGAGCGAGAACAGCCAAGAAAT
AATCGTATAAAACACAAAATCTCGCATCGTATATCTACACTAAGCAGTTATAACAACTGTTTAGGCAGCGATAC
AAGAGCTTGGCACAGTGAGTGAGCTGACAAAGTCCTGCAAATCTCCTTGACACTTGAAGGTGAAGGGTTT
GCTGATCATCATAAAGGGCTTGCAGCCAGGTTGGCAGAAGGCACTCAATTCAACAGACTCGCACTGGCCCT
TGGTTGAGACACCCTCGTTGGTGAGTTTCTCGAAGCGGGTGCACTTGGTAGGGGCATTGGTGTCGTACTCG

GGGAGGTGGGTGTGATATTGTTGGTCAATCGGTTGGCAAGTCTCGGTCTCCTTCTCAAGACGGCGAGAAAT
CTCATGGTCCATGTTTGAGGCAGAGCAGTCCTTGAAAGAGTTCAACTTGAAGCTTGCGGCCATAAGTTCGC
CATCAGAAACGGCACAACGTTGGGGAGTCTTGAATTCACCTGCAACTTCTTGGTTGAAATCACCACACATAC
CGCAGGTTCTGCCACGCAGATGTTGAGGAGCGGTCAACTCGACGTGAGATCCTTGGACTTTGATGAAGAA
GTGCATGTTCTTGATTTCCATGATGAGGCTATCGGGATGGGGGTAGACAACAGCCTTGATTTCCTTGGCTCCT
TCCTCGCGGATTTCGAAGCGGCTCTCCTTGTCCAAGCTCTTGACTTCGGTCTTGTCTCCGTTGATGGTGACGT
AGCCGGTAGGATCGAGCTCGACGGTGTCCTTACCATAGATAACGGTGACAATCTTTTGTCCCTGATGACCCT
GGTGGGCAGTAACGGCGATTTCGGATTGCTTATGGCAATCAGTCATCAAGACATGGGGGCATTCGCTGACA
GTGTAGTTGTAATGAACACCATCATAAGTGTAAACGGCATCGGGTCCAACGTAGCATCTGGCTTCGGTAACA
CCTCCAGAAGTCAGGCTGGCAGCTTCACGGATGAAGTTGCTTCCGGCGCTCAGAGGGAAGAAGACCTTCG
AGTAAGCAAAGGTCAGCGGTGAGAAACGAGCCCATTGGTTAACAGTTTCATCTCCACGGCGGAAATTGACG
GCAACGATAGTTTCGGTGGGACGGACGAAGGTCATGTTGCTGGCTCCGGTGTAGGGATCACGTTTGATGGT
CCAGGTGGCACGGTTGGCAGCGTTGGACTGTCCTTGGATGTGTTTCACGGTGAAAGGATACAGTCTGTAGT
TGATCCATTGGCGGGCAGCGTTCAACCAGTAGCTAGCAGTCTCCGACAAGTTATCAGATTCGGCGCTAAGTT
CGTAGTTGTTGTAAGTTTGATCCATGCGGCGGGCCTCAGTGCAAGCAGGGCTTCCGTACATGAATCCGGTG
GCCAAATCCTTCTCGCACTGTTGGCCAGCAGGGCTGTACATGGCAGCCTTGGCGGCATAGTCGTCGCGGTA
GACCTTAGCCTTGAAGCGGATCTTGGACTGATCGCACTGCTCTCCGAAGGCAATCTGGTCCTCTTCAGTTAG
ATAAAGAACATCCTTGCTGAATCCGTAAGTGGGAGGCTTGTTCCAGTTGCGGGTGGCAGTGTGGCAGACGG
CGAAATCGACCTTCTTGTTGGCACGAGAGCCGGAAGTGGTGTATTTCTCGATTTGGATATCGGTGAAGTCCT
TGGTGAACCAAGCATTCTTGGACATACCAACGGTGGCGTTGAAGAAAATGAAAGATCCATCCTTCTGCTCGG
CAACGAGTCCGGTCTTCATTAGGTGAGCGTTACCGCTTTCCAGGTTCTTGAAGAGACGTCCGAGAACGGGG
CGGAATTCAGGGGCAATAGTGCTGGGGGAGATCACCTCGGGCTCTGATGGAACCCTGGAGTGGGTGGATC
CTGACTCGTAAACGACGGTGTGCTGGCAAGTTTTGGTGGCGTATTGGTACTGGAAGTAGGTAGAGACAGAG
CGGGCGCGAGTGCCGGATGGCTCATAACGCAAGACGTACTTGCTGTTGCGGATTTCAAGGGGAACGAATCC
AAGGTTGGAGAAACCGTTGATGTCCCATTTCTGGAAAGCCTGGAAGATAGACATCTTGTCGGAAAACTCGT
ATTCGCTGTGTTCCATAAACTTGACGTTGACACCGAAGCGGTCACCCAGGGGGAATTCACGCTTGATTGGTT
TGTTAGTAACGCTGATGATGGCAGTGGCTTGATCCTCGAGGGTAGGTTTAACGCTATCAGCGACATTGCGAG
TGACGGTGTAAGGCTTGACGTGGTAGTAGAGGAGATCGGTGACTTTATTGCCAGGAGTCCAGGTAACCTTC
ATCTGGCCGTTGTTAGGATTGTAGTTGAAGTTCAGCTCCTTGGGGGCGCGAGAATCGACGCGGACGTCCAC
ACCGGAAGCGATGTAGTTACCGTTGAAGGGGATTTCGACGCGGATTTCGGAACTCAACTTCCAGCTCAATT
CAGCGTTGACATCAGACTTGATGCCACCCTTACCGTCACCCTTCATGTTTCCTTGCATGGAAACGAGAATGG
GGAGGGTAGCGAGGAAGCGCATGGGCAGACCCAATTCAGTGGGGAAGCGGGCATTGTATTCAGCCAACAT
GCGGTACTTGGTGACGCTCACTTCGTACGGTTTGTTTTGGTACTTCATGGCCTCGTTGATCAAAGCGGGAAT
AGTCTCTTCGAGCTTCTCCATGTTGAAGAAACGGTTGATTGAGCCCATCAAGTTGATGTGAACGAGTCCAGC
GCGCTTGTCAGCAGGGCGAGGAGTGGCGTTCAACTCTTCCCAGATTCCTCTCAGGTTTTCAATGTTTTCACC
AGCGCTGCGGGAAGACATGGTTTCATACTTGTTGGTGAGGCGGCCGAAAAGTTTGCCGAAAATGTTTTGCC
AAACTTCGGACATGTTGTAGTTCCACAGGTAAACACTGCCGGCTTCAGCGGAGAAGGGGCCGAGCTGGTA
GTAATAACGGTTAGCCAAGAAGACGGGCCAGTGTTCATCGGCGACGCGGAAGTTGGGAGAGTACATCATA
ACGGCGACACCCTCTTCCATGTGGGAAGAACGGATATCAGCGAAGGAGTAAGGAAGACCGAAAGGAGCA
GGCTTAACCATGGGCCAGGCAACGTTAGCCTTCTTGATCAGCTCCTCCAGGAAAGGAACGGATGGGGGCAT
GTTGGTCATGGATTCAATCAGGCTGCGGGTGAATGCGGCAACTTGGCGGTTGGGTTCAAACCAAGTGCTAG
AAGCGAATTTCTGCCAAATAGACTGTGGAGCATTGGACATGAACAAGAGACTCATGGCAGCCATGCGGACT
TCAGCACGTTCGGCGGTGTTGCTGGCCAAGTTAACCAAGATGGGATGGATCTTTTCAGGAACGACGAAAGC
AACACGGTGGAGGGAGAGGATGGCGCGAACACGCTCAGCGGTGTCATCGAACTTGCCGGGAGTTCCGCG

GATGATGGGCAGCAAAATGGGGACGATTTCATCGACACCCAAAGAACCGAAAGCGGCCAAAGTAACAATA
CGATCGGTGGTGGTTTTGCTAGATTCGAGTTCTCTAACGAGCCAAGGCAAGAATTCATCGATGACGACGGG
GTCCTTGTGGTCGCAGAAATCACCCATGACGGAGACGGGGTATTTCTTGGCGGCCAAGAAGCGAGAGCCG
CAGACGGAATTCAACAAATCAGCGATAGCAGCCAAAGTAGTCTGCTTCATCTCAGTGCTGGCCTGAACCGG
GCCGGACTTCAACAGATTGATGAATTCATGGAGAAGCTGACGAGTCGGGGTCTTGGCATAGTAGCCGAGG
GCGGCCAAAGCCCATGTTGCCTTAGCACCGGTGATTTGCTCGGTTTCGACCAACTCCTTGATCAACATAACA
GCAGGGTTGCTGCCTGACTGGACAAGGCAGTCGACAAACAGAGTCACGGTGGGGTAATCCAAGGTCTTAA
CTTCTTGCCACAAGGATTTCAGTTCATCCAGAGAGAACATGGGCAGAACGCGACCAATGACGGCCAAACGT
TCCAGGTTGTCCTTCTCGGAATCGGGGGTCTCCTTCAGGTCCTTGGCGACGGTGCGCAGGGCAGCAATGAT
GTTCTTGTGCAGAGCAGAAACGGGAAGAGGGCTGTTGATATAAGCATCGACCAAGTTGGGTTGAGCGGGG
GTTCCATCGTGATTCACTTGGTGGAAGTCACCGAAGACGTAAGACAAGGTCTTGTGTTCCTTAGGTGAGGA
CGGGGTGGCGAAACGTGAGCCGAAGCTCTCCATCTTCTCCAAAGACCAGTCAATGATGACAGAGTTGGTCA
ATTCATGGGTTTCAGTGGCGAAGGGCTGGACCTCCAAATCGCTGAAGCCACTGGCATGGACGAAAGTCAG
CTTGGGGAAAGACTCTCCGCAAGCGATGTATTTGTAGATTGTGGAGCGCTTCATGAAGTTACCGCAATCAGT
CTTGCCAAAATCGCAGCTGTGGGTAGCGGGATTGGCAGTTGACCAGACAGGGTTGTGTTGGCAGCGGTTG
TAGTCCTTGGACTTGACAATCTTGTAAACGGGCTTATCCTTGCAGATGGCGTCGAAAGAGCCCAACTTGAAA
GCATCGGTGATGTAGACTTGGGAGAGGGGCAGACGGGTAACGGAGATGGTAGTGCTGCAGTCACCGCTAA
TGGAATCCTCGAAGACAGTGAAAGTTTTGGTTTCTTCCTCTGCGGGGACGGATTCATAGTTGAAAATGCCAT
CACGACGGACACCAGTAAGGTCGAGTTGCAACTGGCTTGCGATGCTCTTCTTGTAGTTGACAACCCACAGG
GGTTCTTCAGCATCGATTTCGATTCCAGTGAACGATCCCTTGTTATGGGTGACGACGAATGGAGCTCCAAAG
TGTTCATCGTTGACGAACTGGTCCTCCTCACCTTCAATCTCTTCAGGGTGAGCAGGTTGGATAACGTAACCAC
TGTCGCTGATTTCACCCTGGAAGTAGGGGTTCTTCACGAAGTTTCCGTTACGTTTAATGCGTCCATCGGTCAA
CTTGACGACAATGGTGTTCTCATCGATACTCTGGACGATAACGCGGCTTTGCAGTTCAACAACAGCGTACTG
TTGTTTGAGATGATACATGCCAGCTGCTGAACGTCCAGCATATTTGTACTCATACTGAGTGCCCGGTTCCCATC
CCAAAGTCAGGCACAACAGCGCCGACAAAACAAGGAGAGAGGACTGCAGTTTGCCCATCTCGACGTCTTT
GCTCAAGCCTCTTCAAACGACTTGTGCTGCGATACGGCCGCTCCATCGCCTCTTAAATAGCCCAGTAAAATGA
AGATAATACGCATCACTCGTATTTTTTTCTTCCTTCGCTCGAAACAACCACAAATCGCTGATAATCGTCGATGA
CCCTTTTCCAGATAGTAATGTAGCCAATATGGC

>2 c23345_g1
CGGGGGGGTAGTTCATTGAATTCATGCCAAGTACCAACACCGAGTGGTTCGGGCTGATAAAAATGAAACTC
CTTGCACTCTTTCTCGTTATTGCCGTCGGATGTTGCGCTGGAAAATCTGTTGAGGGCCCTCGTCGTGCTTTGG
TTACCATCCAGGGAACTGGTGATGTTAACGGAAAGCTGATGCTGGAACAAGCCTCAATCAATAGCCCCGTCA
AGATCCGTGGTGTCATTTATGGTTTGGAGCCCGGTCTCCATGCCATCCATGCTCACACTGGAACCTCATTGGG
CGTTCAGTGCGAAAATGTCGGTGAACGACTTTTCGATGCTGACAAGCGCGAACACGAGAAATTGGCTGGCC
ATCTCGGCAACGTCAAGACATTCACGGGTGTGCCTTCTCGCACCGACATCAGCCTGATCTCCTCGCTGGTGT
CCCTGTATGAAGACCATTCTCGCAGTGTTTTGAACCAAGTTCTCGTCGTCCATGCTCTTCCCGATGATTTGGC
CCTCGTCAACAAAAAAGTCAGGGATTTGCCTGAAAGCCACATTTTGGCTTGCGGTCTGATCCTCTCCAACAA
AGTTGAGGCTTTCCCGTCCATCGAGAAGGAGTCTATCCTTTTGGGTTTGGAGACACCTTTCAAAATCGGCAA
GATCGAACAACGCATCTTGAGCGGAGAACAATCGTGGGAAAATGTCCGTCATCCCCATTACGGAGCCGTCG
AAGGCCAGAAGGTGCCCTCCAACTTGAACTTGGAAGGTGTCGAACGCCCATCCAACGTCGATAGCTTGACC
CTCGAAACTGCTGCCCCGACCCGCAAAGTCTGGATGGCCGGTTACACTTACGAATACGACTACGCTGGCTG
GACTTCAACTGGTATCATGGGCATCAGCACTAAAGTGTCTGGAGGTTCCATCAAGGGTCGTCTGACCATTGA
GCCCGTCGATGAAAGCACCGCCGTTGTTGCTCTGCTTGCCACCAAGGGTAAGCAATTCAACGAGGACGTGA

TGGAGAAATACTCTGAAGTCGATCCCGGACAGGAAGTCCGCATCATCGACCAAGAACACCTCGAGAAACCC
TTCCAAGTCAAATTTGTTTCCGGCAAGGTTGAGACCGTCGCCATTGGCAAAGAAGAGCCTTTGTGGATCGT
CAACTTCAAGCGTGCTTTGGCTGCCCAGATCCAACTCCAACTTGATGGAGTCTCTGGCGTCTTCCAACAGGC
TGAATACGACAACTACTATGCCGAGAACACCGTCTACCACGCCATGGAGGGATGTGCTACTGGTGAGTGCCA
GACTTGGTACCACATCAGCCGTCTGCCCGTCGAAGTTGTTGAGGCTGAGCCTAAACTTCTGCCCGCACCTGA
GCTTTGCCAGAACTTCCCCGTTTACGAAATCGTGAAGAACCGCGACTTGGACAACTGCCGCATTTTGCCCAT
TTTCAACTACAACAGCAACCAGGGACTCCGCTGCAACTTGGTCAATGGCGCCGGTTGCGAGAACAAAATTT
CGCACTCTGACACTGTTAGAATCATCGGTTGTACTTCCAACGAGGGCCACTTCATCGTCCAACGCATCAAGTC
CATTGACAAGTTGATCGTGAAGCCCTTCAGCTACGAAACTGAAGCCACCGAAGGTTTGACCGTCCAGCATTT
GACCCTGAGATCTGCCACCCCAACTGGCTACTCCAAGCTCGTTTCTCGCATCTCCTCTGATGTTCACATCTACC
AGACCCTGGCCTACTCCTACGATGACGACTACAAGACCCACGGACCCTTGATGGGCAAGCCTACCCTTCGCA
ATGTCAATTCTCCGATGATCATGGAGGTCGAGCCTGAGATCCTCAAGAAAGAGGCTCTCCGCCTGCTCTCTG
AAATCATCAGCGATGTTGAGTCTGAGGCTTACTACGTCGATCCCGCTACCAAATACACTTCTGACAAGATCAA
CATGCTCCGCAGAGCTTTGGCTTCCCTTGACTACAACGAGCTCATGGCTTTCGTTGCCCAGGTTTGGGAGAA
CAAAGAATGGAGCACCTCCAACCAAATTGTTGTCGACGCTTTGATGCTTACTGGCACCAACCCATCGTTGAT
GTTGGTCCGCGAATACATTCTCCAGGGTAAGATCGTTGGTGAGCAAGCCGTTCAGGCTATCTCTGCCCTCGT
CCCCACTGTTGAAACTCCCACCAAGGAGCTTTTGGCCAGCTGGATGGAATTCTTGAAATCCGAAGTCGTTCA
GAGCCACCGCCAACTCAAGATCACCACTGCCCTGTCCCTGTCTCGCTTGGTCTACCAGGCCTGCGTCAACAC
CACCCACGGCTTGAACATGTTCCCCAAGTTGGTTATGGGAGAGTTCTGCAACCCCAGTGACTCAATCGTCGC
CAGCCAGTTGGTTCCATACTTGGCCGAACAGGCTAAGATTGCCAAAGATGCCGGTGAGCGCATGGCTTTCT
TGACCGCTTTGGGTAACATCGGACACGAGATCATCGTTCCCTACGTCAAGCCTTTCATCACCTCCTGCGAGCC
CAGCTCTCACTACGAAAGCGAATGGTACGAGCGCAACCAACGCGACTTGGTCTCCTTGTCAAAGAAGGAAA
TGAGAAAGAAGTGGTTGGAAGCCAAGAAGACCCTCAACTTGAAAAAGTACGAACAAGAGCAGGAAGATA
TCGTCTTCGCCCGCTCCGAATCCGAGGACTTTGAAGATGAGGCTCTCTGCAACCTCGTCCGCTCCAAGGCTA
TCTTCGCCCTCAGCAATTTGGCCGTTGAGAAGAAGGAAATTGTTGGCACTTTGTTGATGCCCATCTTCTTCA
ACAAGGCTGAGGAAACTGAGGTCCGTTTGGCCGCTCTCACTCTGCTCTTCGTCAGCAACCCACCCCAGGCT
TTCTGGTCCCGTGTTGCTTTGAGCACCTGGTACGAGCCCAACGATCAGATCTCCCACTTCATCTACACTACCA
TCGCCAGCCGCGTCGCCAACAAGAACCCGCTTAACCGCGAGGAGGCTGTCCGCGCCGAAGCCGTCATTGC
TTTGATGAAGCCCATGTTCTGGACTTCCCACGCTGCCCTCAACTACCAAAAGGCCGGTTACTCTGAAAAGAC
CCGTCTTGGATACGTTACCGAAACCGTCAACTTCCCCGGATTCGAATCTTTCGTCCCCTCTCACCACTACAGC
AGCTTGTCCGTCGCTATGGGACCCTGGTTCACCAAGCTGATGGAATTCAGCATTGACAGCAAGCACGCTGA
GAAGTTCATCGATCGTTTGGTCGGAAAGCCCGGTCTCCGTTTCAAGTCCAACAAGGACGAATCTTCCATTAT
CAGCCCCGAATTGGAGAAGATCCATGAAGAGTTGAAAATTGAGGCTCGTGCCACTGGTCAACCCGAGTTGT
ACATCTACCTGAACTTCTTGGACAACTACCAACGTTTCTTCACCATCAACCCCAACACCATCTTCAGAATGATT
GAGAAACAGATCCTGCAATCCGGATTCCGTGAGAACGCCGGCAAATTGGAGATCAACTTCCACAAGTACTT
GCCTCTTTTGGACTCGTTCGCCCGCATTCCATCTGCCATGGGTTTGGCTTACACCTGGACTAGCCACCACAGT
GTCCTCGTTTCACTCAAGTCTAAGATCGAGGGTGGCTTTGCCATGTCATCCTGGAGCGCCAAGTTGGAAGGT
GCTCTGAAGCCCGTTGTCGTTTCCAAGATGAGCACTCGTCTGATGGTTGACACCCCGTTCACCCGCGCTTTC
CCCACCACTGGTGTTGATATGGAATGGGCTGCTGCTCTGCCCGGTCGCTTCCTCGTCGAAGGTGACATCAAG
ACTGGCAAGATCCAAACCACCTGGGAAACCTTGGGCGATAAGCTCCGCGTCGTCAAGCACTCCGTTGTTCC
TTTCACTACCATCCGCAAGATCACTGATTTCACCCCCGCTGCTCTTCTCTCCGAGACCAAGCGCATCTCTTACG
TCGAGGAGCCTAAGGAGAACAAGGTCATTTTCGGTGAGAAACACCTCGGCATGAACTTCGTTTTTGTTGAG
CGTGGTGAACGCTTGGCTGTTGTTCAGCCATCAGTTTACAGCAAGGATTGGTTCGGTACCCTGGCTTTCGCT
GCCATGCCCTCCACCCTCCGCCAACGCGAATGGAGCTTGTACCTCGACAATGCCTCGTCCGAGACCAAGGCT

ATCAAGACCATCATCTCTATCTCTACCAAGTCTGACTCCAAGTTCGAAACTCCTTTGACCGGCCACCTCCATGC
CAAGTCTCTCTTCGACACCATTTACGGTGAGCAGATCATGAGCCAAGAGTACGTTAAACAGCAGGACAGCC
AATGGGAATCGCAGAAGTTCCAACACATCTTCCAGACCCTGACCAACCCCACCGGATATTCTTTGGATTTCAC
CGCCGAACTTGTCCCCAAGTCTTCCTCGATCAAGGCTCGCCGCATCGGCTCCTCTGTCGTCTACGGCATGGAT
GGAAAGTCCCACCGTGGAAGCCTTATGGTTGAGAGACGCGATGAAGTCGAAGGCCAGAACAATTTCGTGC
TTTGCGCCGAAGTTGACGCTCAATTCCCCGACAACTTGGTTTTCAAACGCAAAGAACTGATCAAGGATGAA
ACCGAACGTCGTTCTACCATCAAGATTGGCTTCGGAAAGTCTTGCACTGATGATCGCAAGATTACTGTTGCTA
CCACCTGGACTCGCAGCGAGGAAGACATCTCTCCTTCCCTGCGCAACCAGTGGGAAAAGACCCAGTGCCA
GAAACAGGAAACTTTGGGCCGTGGTATGAGCGATGAATGCATTGCTGCCCGCCGTCTGTCCTCAATCCTGAA
CAAGGCCGTCATGACCATCAAGTACAACGAGATGCCCGCTGCTGTTCTCAATGCCACCATCAAGGCCTCCAA
CCTCGTCCGCCACTGGTTGGGTCCCTACATGTCTGACAACCAAGTCGAAGTTATGAACACCCAGAACCAGAT
CACCGTCGAGAGTGTCTACTACCCGTTGGCTGGATCAATGGATGTTAAGGTTTTCAAGCCTTACTCCAATGTT
TTCTTCCGCGGAATCGAAATCCACCCCATCGCTGAAGTAATCTTGCCCAAGCGTATGGCTGTTCCCCGCTCTG
TTTTGGCTGCCCCCGGCGTTTGCTTGATTGGCTCCGAGACTGTCACCACCTTCGATGGCCTTTTCTACAACGC
CTCCTTCTCCGGATGCGATCAGCTCTTGACCAAAGATTGCTCTGGCCGCTACAAGTTTGCCGTGCTCTCCCGC
GTTGAAGGAGACAAGAAGATCGTCACTGTCCTGTTGAACAAGGAGAAAATCGAGATTTTCCCCGCTCAGCA
GAAGGTCAACGTCAACGGTATGGAAATCTCCGTAACTGGCGAGTCTTACACCGTCAAGAACGCTGAAAACG
AAGTTTTGGCCGTCATCAAGAAAACCGCTGATAACTTCATTGAAGTCGACTCTCCCATCTCCCACATGATCCG
TGTTTTGACCGACGCTAAGGAAGTCGTTGTTTTGGCTTCTCCCATCCACCGTGGCCGTCTCTGTGGTCTGTGC
GGCTCCCAGAATGGCGACAAGGTTACCGACCTTACTGGCCCCCGCCAGTGCGCTATCCCCCGTGATCTGATG
GATGTTGCCTACGAGCTGAGACAACCCGCCGGATGCAAAAGCGATATCTCTTCCAGAGATGTTGCTGAGCTT
CGTCGCATCCAGGAAGAATGCCTGAAGGAGAAATCCGAGACTGTTTTCGGCATTTCCGATGTCACCCCCTTG
TTGCCCAAGTTCCAACAGAACATCTTGTCCAGCCAGACCATCCGCCGCCCTTCGCAGTGGACTGTCTACCGC
AACAAGATGGTTGTCCAAGACAACAAGCGTTGCTTCTCCACCGAGTCAGTGCCCAAGTGCGCTGAGGGTGC
TCGTGCACAGGAAACCGTTGAGAAGAAGCTGGGTTTCCACTGTCTTCCCAAGAACATCTTGTCCGAGAAGT
TGAACGAGGAGATGTCTTCCCGTCCTCTTGATGAGCTGATGGGCAAGCAAGTCGACATGGTGCGCGTCTAC
TCGGTGCCTACCACTTGCCTTCCCTTCTAAATCAACAAATCAAAAATCTTTTTTGTACGAGCAAGGAAATTGT
TTTTACATTCTTCCGTGTTCCCCTAACGACCCCGCTACCCCACGTCAAATAATGACCTATGAAAATGCTAAGAT
ATATTAATTTAGAAGTTGGATAGACTCTCATTATATCGAGAGTATTCAAGGTATGGAATTGTTTTGAAGGCGCC
CAGCTTCTGTTGAGACTACGCCAGGGTGATGTAATCTATATGAAAAAATATATTCCAAGACAATAAATAAACA
AGCAGCATCACAAAATCCAAAAAAAAAAAAAAAAAAAAAA

>3 c23476_g2

CGTGGCCGAAATGCAAGTAAACACAATTATTTTGTTCTTCAATGTCGCTATCAGCTCCTGTTTCGCTTCTGGTA
AACTCGGCTGGAGGACATTGGTACCATCTGCCCCACAGTGGGAGTGGGTGGCATAGTTTAGGTGGATGGCA
TTGCTTGAGTTATTGGTGATGCAAAGAATCACACCACCATACGAGAACGGGAAAACCAATCAGCTCGAAGC
AAACACTGTTTCCATGCAAATGCGACATCTTTTTTAAGTACAAGAGAGACAGATCCTGTTGCTTGTTACCAGT
ATATAAAGAGGGATTGCGGACCGGACCAAGTCGCTGTCGCCAGTGATCCCCTAGGACGCTAACACGCACGA
TGGCACGTCTGAGCCTGTTGGCCCTCGCCCTTCTTCTCGTCGCCGGCCAATGCCGTGCGGAAGGTTTGTTTG
AAGATGGCCAAGAGTACCAATACAGTTATCTTACCTTCACCACATCCGGCGTTCGTGAACCTAATCCCTCTGG
CTCCTCCTTCGGCATCCGTGGCAATTTGCTTATCCAGAAACAGGCCGGAGAAGCTATCGTCAAGCTTTCGGA
TGTTACTTTGGGTATGCACAATGGACCCGACTCACTTCTCTCGACTGTCAAGTTCTTGAAGAAGCCCGAGTT
GGCTATGTTGGAAAAACCCTTCAAAATTTCGTTCTTGAACGGAAAGATCACTGGATTCGATGCTGACGCTTC
GGATGCTGAATGGTCAATCAACATCAAGAAGGGTTTGGCTACCAAGTTGCAAATGGATGTCGTTGCCGGAG

AGATTGGCAAAGGAGACACTGCATTCTTACGTACTGTTGAGGATACCGTTGTGGGCACCTGTAACACCACCT
ACTCTTTCGGAGGCTCCGAGAAGGGCCGTCTGATGTTGATTAAGCAGCGTACCCAAAGCGAATGTACCAAC
GTCCCTCGCATGAGTCACAACAGCTTTGGCGCCACCAACTGTGCCGGAAAGTTGCAAGACGAACTCGTTGC
CACCACTCAGCTTTACTACCAGCTTAGCCGCGGATCCAGCAGCAACACTGCCAAGGCTCACGTCATTTCATC
CTTTGGCTACCAGGTTCTGCAATGGCACCCACCTGCGGGATCTCCCTTCTACAACTTGGCCAACACCACTTTG
GTGCTTAAGTCTTCCGGACCCATCGCTAAGCCAATTTCCGCTTCTGGCTTGACCAAGCACTATGGCAGTCTCC
GTTACACCCTAAAGCGCCCAATTCCTACTCCTGAGGATCTTGACTTGAGTCGTGAAGAAGACTTTTTGCACC
CCGAGGAGCCCCAATCCCAACAAGCTCTGCGCGCTAAGGCTGTTCCCGTCTTGACTAAATTGAAGGAAGTC
ATTGGATCAGGTGCTCTTAATGAGGAAACCCTCTTGGATCCCACTGCCATGGCCGCCATTCACCTTTTCCAAG
CCATGAGTTTGGAAAGTTTGAAAGCTGTCTGGAAAAACGTTGAAAGCGACGATGAGCTCAAGAATTTGTTT
ACCGAGATCCTCCCAACTACTGGTACTAACCCTGCTGCTTTGATGGTCAAGGAATTGATCCTGAGTGGCAAA
CTCTCCGATATGGAAGCCCGCCGTATGGTTGCTTTCCTACCCTACTACCTGCGCATGCCCTCTGAGAAATTGTT
GACTTCCTGGGAGGACCTGCTGAAGGAGAGCCCAAGCATCAAGACCAAGGAACTGAGAGGTGCGATCGC
TTTGGCTTTCGGTCATCTCGTTGGCGTCACCTGCAACGCCAACAGACTTCGCCCTTGCAAAACTGACACCAT
CAACAAGTACACTCGCATGTCCTACGAAGCTTTCAAATCGGCCAAGACCCACCCTGAGATGATTGTTGCCCT
GAGCACGCTGCGCAACACCAACTTGATTCCCGCCATCGAGCGTCTCATCCCTCACGTCAAGAGCGGCTCCGT
TCCTCACGCCGTCCGCCCGCACGTCATTTTCGCTCTGCAGCCCATTGCCGCCGTCAACCGCAACAAATTCCTG
TCTGCTGTCCTCCCCCTGATCCTCAATGCCACCGAAACCACCGAAATCCGTATTGCCGCCATCTCCACCCTGTT
CCGCGTCCAACCCACTTTCTTGGAGTTGCAGCAGTTGATCGCTGGTGCCATTTGGGAACGCAATCAGGAAG
TGCTTAACTTCATGATGACCACTTTCCGCAACTACGCTGATTCCAAGAATCCCTGCGTTAAGCCCATCGCCAAT
CAGTTGCAGTTGTTGTTGCGTCGCGTCGCCCACATCAAGACCAACTTCTTCCGTTCAAGCAACCGTGTCTTC
GACTTCCAGGACCAGAAATATGGTTTCGGTGGTGGTCTGCAACTTGCCACAGTCTACGGTGAAGAATCCCG
CGCACCTCTCATCATTTCCGGACGTGCCAGTTACCGCATTTCTGAATTCAGCTATGTTCCTCTTGAGATTATGA
TCCGTTTGGAAGGTGTTGAGGATGCATATGTCCGTCTCTTCCGCAAATTGGACCCTAAGGACTTCAAGCTTG
ATACCCTGAAGGATCTTTTGCAAAAGACAATGAAGATTGTCCCCCGCCAACAGGCTCCCATGAAAATGGAAA
TCTTGTTGCGTTCCCAAGGATACACTTTGATGTACCGTCACATGGGAATGGAAGAAATCGCTGGTCTGATGG
AAGGCAAAGGATTGGTCGAAATGATTTCTCGTGGCTTGAAAATGACTCGCAATATGGTCATGCTCGGTGGGC
AACATATCAGCTGGAGGGCTAACGATGCTGGTCTTCCCGTCGGTGTTGGTCTCAGCACTCCTGGATTCGCCC
GTCACCAGTTGGCTTACGGAAATGTCAATCAACCAGCGAAGCTCGGCCGCTCCATCCTGGCTGATTTGGACA
TCAATCTGCAGGTGGTCACTTACATGGTCGCCTACAACCCGCTCGGAGTTTCCCAGGGAATCATCAAGGCCC
GTGGATCCCGCATCCATTTGCCCGCTAACCTTTTGGTTGGTTTCTCACCTGCCGACAGCCAAGTTGAAATCAA
GATGAATACTCCCACCGAAGAGAAACCTTTGTCTTACTTGTTTAGCAGCAAAACTTTCGCTTTCATGTGGGG
CAAAGATGACTCCAAAGCCCTTTCTTACCTTAAGGATACCTGCTCAGAATGCGAATCTAAAGCTCTGGTAACT
CGTGGCGAACAATTCCGCAAAGGTCAAGTTATTCGCGAAAACATCAACGAACTTTTGGGTATGGAATCTCAC
GTCGAAGTTTACAACTGCGAAACCTACACTGGCAAAGCCTCCATCGCCAAGGTGATCTACGAATCATTCAAG
CCCTCAGAAATCAATTCGCACGGCAGCGTGCCCGGTTTCTTGGTCATGGGATTCATGCAAATGCGCAACTAC
TTCTACCAATACCCACCCACCGGCGCTTGTTCCATGAAGGCCGTTCTACACAGGACTCAAGTAAACCCGGCT
GAAGCTATCGAGATTAAGCTCAAGATGGATTCCGCTGTACCAGCTGGCAAGAAGGCCGCCCCAGGCAGAA
GTTTCAGCAACGTTAAGGGAGCCATCACTTTGTTGGGCACTCCTGAGCGCAAATGGAACGTGGAAGTCAAT
GTCGAGAGTGAGCCTTTCAATGTCAAATCGCAGGTCGCCGTGAAAATTGCCCGTCTCGCAAACCGTGAACT
GAACGTTCCCAACCGCGCTCTCTGCGTGAACGTCAAAACCGCTTGGGCCGCTTTGCCAGAGGACATTTTCG
AAACTCCGTCATCCATCGAGCCTTCCGTTCAGCGTGAAGTTTCTTTCGTTTGGGGAGAAGCTCCCGCTGACC
AGTGCCCTAAAGCTAATGCCAAGGACATCTCCACGATTTTGATCAAGGTTCAGGGTAACATCACCGACGCTC
AAAGAGAAGCCGCCACCAGCCGCAACACCTATCCCTATGATCGTTGCGATTTGGACCGCAACGATGCCGGC

CGTTCTGGAATCGTTGGTCCCATGACACAGGCTTGTTACGAAGCTGTTCTCCACTACGCTACTCCTCGCAGCT
ATGTTTTGGACATCAATTACGCCAACATGTCCCCCCGTGGTCAGATGGCTCTTCTCCGTATCGACACCATGCTA
AAGGCCTCCTTGTTGCCCTACTGGAGCATGCATGCCCCACATGGTCCTACCGCCAGCGCCAAGAAAGTCGCC
GGTGCTGGCCACATTGAATTGAAAATGGACGTTAACGAAGATGATGTTGACTTGCATGTCCACACTGACTTG
ATGCACAGCCATTACGAGAACGTTGATGTTCTCAAGAATTTGGGTATGGCTTTGCGTAACGCCCGTCTGCCC
ATGTCTCAAATGTTCGCCATCAAGGCCGGTTTCGTTGGTATTTGCGACGTTGCTCCCAAAGCTGTTGTCACCT
TCGACAATGTAACCATGAGCACTGACCTGCCCACTTGCTACACGCTCATCTCGGCTGACTGTTCGCCCACCCC
TCGCTACGCCGTTTTTGCCAAGAAAACTACTGGTTCATCTCTTCCGCTGGCTGTCAAGATCTACGCTGGTGGC
CACACCCTCGAACTGAACCCAGTCTCCAGCGGCGTGGAAGTCAAGGCTAACGATAAGGTTGTCAAGGTCGA
CGCTAACAAGCCCTACGTCTTGAGCGACAAGGACAACGTGATCCAGTACATTGTCGTTAGCAAGATCGGCG
CTCGTTACTTTGTCCAGGTTCCAGTTCTCAAGTTGACCTTCCGCTATACTGGCGATGACATCACTAGCATGATC
CCCGCCACCCATCGCTCTCAACATTGCGGTCTTTGCGGTGATTACAACGGTCAATTTTCTCGCGAATTGGTCA
GTCCATCCGGTTGCAATGTTAAGGATGCCACCGACTTGGCCAGGTCCTATGTTTTGAGAGACAACAAATGCA
AGGACAACATCCCAACTCCTCCTTGTGTTGCTGAGCCTTTGAGCATTGGTGAGAGAAAGACCCGATCCGTC
GGAATTGTTGGTCTTATGGATAAATTTATCCGTTCTTAAAATGAACATAATCCGTCACGAAATTCTCGTTGCCT
TATAACTGACGAATCGCAACTCCTGAATAATTTTTTTTTCTCTATTCAATTTTCGACTTTATCGTGGAGCCTTAC
AGAGCAAGGTCGACAATCCTTTTGAGTTTAGTTGACCCTGTGTGTTCTCTAACTGTTCTCAAAATTGACTACC
TGAATACAACTAAACAGGAATTTAAAAAAAAAAAAAAAAAAA

>4 c18434_g2
TACATGTGCTTTCGTATAAAAAGACGATTGAGAATCAAACTCGGCACTTGATTTGCAGCTTCTGTTCTATTCA
AGCAGTCAAAATGAAAGTCTTGATCCTTAGCGTTTTCTTGGCCTGTGCCTCTGCCCAGTATTACCCTACTGGC
TACTTTGGCTATCCCGCTTTCACTAGCCAATACCACGCCCAAGATGTTCTCGGACAGGCCTCTTATGGTTTCG
CCCACCCCGGACAGTCTGCTGCCCATTACCGTGACGCTTACGGAAATCAAGTAGGATCTTACGCTTACATCAA
CCCGGAAGGAAAGCACGTCAGCGTTTCATACACGGCTGATCACCGAGGCTTCCGCGTCTTGTCCAACGACT
TGCCTGTTGCTCCCGTTGCTCCCGTTGTCGAGCTCAAGTCTCCCGAACCCGTTCAGGACACCCCTGAAGTAG
CTGCTGCCAAAGCTGAGCTCTTCAGGCTCCAGAAGGAAGCATCAACTGCCGCAGCCGGAAGCCGTAAGAA
GCGCCAGGTTCCTTTCTACCCAGCTGCTCTGCCCTACACCGCACCTTACTTCCCTTTCTACCCGACTGCTCCCG
CTCCATTGGTAGCTAAGACCACCGTGAAGGCCGTCACTGCTGAAGCTGATTTGACTGCCAAGACTCCAGCTG
ACACCAACAAATTCGATCTGAAGGAGAAGAGCTATGATGTTGTTACTCCTCTGGCCTACCACACCCCATATTT
CTACTCTTATCCTTTCTATTCCGCTGCTGCCCCCGCTGCTGCCGCTCCCGTTGCCACCGAGAGCTCCCGCAAG
AAGCGTCAGGTTATCGCTGTTCCCGCACCCACCCAGCAGTTCCTTGATGACCTCAAGTACAAGAGCGTCGAC
TTGAACCAGAATGGCATGCCCGATAACGCTGTTGTCCCAGTTGCTCCAGCTTTCCCTGCTCTGCCTTACCTGC
CTTATTCTGGCTATGCTTACTCTACCTACTACCCGACCGCCTACCCTTTCGTCAGCAAATTCTATTAGGAAATTG
CAAACGGATTGACATTGCGATTATCGAACACCCACACCCGTTGAGTAATAACTTTATTGTTGCATATGTGAAG
GGATGATGGCGGGAAAAATATGAATTGGAAATGATTATACATTTTTTTTGAACGATATTGCATTTTCATTTGTG
TTTTCAAAGAGATATGGAAGAAAATGGACTGGGTGATATTCGATCCGATTTACAAATGAAAGCGCTGTCCTG
GTGACAGTTGACAAATGGAATGTAATTCAACCTTATGTTGAATGAATTGTTATTCATTTTTTTACGTTTTTGCAT
TTGCATACACCTGTAGCGTCAGATAAAAGATGGGGTCTGCTGGCAG

>5 c16077_g1
CTGGCCATCATAAATGACACCAGATTCTTCCAGAATTTGCAGCGATCACCATATCCCTTCATCCCATCATGCAA
TACATTGCCGTTGTTGTTCTGGCTCTGGCCGTTGTCGATGCTGGTGTCATTTATGATGGCCAGGCCGGCAATG
TTGCTTACAGCATCAATACTGTACCTTACGCTCTTCCGTACGCCACTCCGCTGCTAAACCCAATCCCTACCGAA

ACCAAAACAGCTGATCCCGCGAAAAACAATGAAGCCATTAAATTTGTATATCCCCAATTTGCAACCTTCGTTC
CTGCTCCACTGTACGGCTCGGCCGTTGTAAACAAGGACGCAGCGGCCGATGCGAAGAAGATCGAAACCGC
AGCAATTGCCACTCCTTACCCCTACCCAACCAGCTTCGTTTACGGGGGTTACCCTTACGCTCCCGTACCTTTT
GGTTATCCTTACGGTGTGTTACCCTTGAACGCTAAGTCTGTTACGGAAATCAAACCCGTCAAAGCTGCAGAA
ACAACCGAAGAGAAAGCAGCCGATAAGGACAAGGTTGCTGTTACTTACGTTGCTTAATCCCGATTTCCTTTT
ATTGTTACCGACGATCCACAATCGACCATGAATTCATGCAATTTATTAAGTTTATTTCCTTGTTATATTGGATGT
TTTAGTTATAGTACTCTCATTGAATAAAATTTTTGTTGCACAAAAAAAAAAAAAAAAAAAA

>6 c2265_g1
GGTCAGACTACATTCTCCACATTATCGGACCATGCTTCAAGAAAAGCCATAGGAAACGCATCAAAATTTGCTA
TTTGAATTGGGCCACCAAAAATGCTCAAACGACGTTTGTTATTCTTTTACATAAAACGCGGCTGATGTCGCTT
CAACGCATGTAAGCCAAACTGTAAATTGAAACCATGTATTTTGATAATCCTCTAACTGATTATTACACACTACC
AGCGTAATCGGTTAATCATGTCGATATAAATTAAAAGGGAGCAAACAACATAAAATTACGAGGAGAAATCGC
CTTAGAAAACAAAACACCCGTAGCAAAAGTCGGCAAATTTTTAAAAAATTCCTTTTTAGCAAACGGTAAATC
CGGATGGAAGACATAGCAGCGCATTGTCAGTTGCGATATTGGCAGTGTTTTTCACAGTTGCGCCAGCGAAA
GAGATCACCGTAGTTACAGTGGTCACCGAGATGGTTGTCGTGAGGCTCAAATTCAGCAGGCCCCTGTCTTCT
TTGCTGGGATCTTCTTGTACGACCTCAGGGGACAGGTTCATCAATTGCGAAGATTCAATTTCAGGCTCGGTG
AAACTGGCATCCTTCACATCAGCAACTGGCAGTGGCTCAACGGCAGACACTTCGAGAGCGATAGGTTGAGC
GGGTTCAATGGATGCCTTAGCTGACTCAGCGTCGGCCAACAGATGTTCCAGATCGCGACGACGACGGGCAC
AAGCTGACGTGGCACGGACTTCAAGCACTGGTGGAATCGCACCTAAATTAGCAGCGGTAGCAACCGAAAAT
TGGGTTGAAGGGATGCATTTGACGGTCGTTGGGGTTGACACGACGGAGACAGAAGTTGAGGTAAAAGTG
GTGGTCGTGGTGAACGGTCGGAAATTCAAAACAGGGGAGGGTAATGGTAAAAAGACCTTCTCGTGGTCTT
GCTGAATTTCGGGTTCGTTCTTGGCTTGGTTATCGATTTCCTTGACTTCAGGGAGAATTTCAACGTCAGCTGC
TGGTTGTCCATCAGCGTAGAACAAAGGCATCAAATGAGCCCTTGGTGCGTAGGGCATTGGCCATACGTATCG
CTGTTGGTAGGACAGGCCGACGAAGCAAGTGAGGAAGATCAGCGTCAATTTCATCTTGTGCAGTTGTTGAA
TGCTGGAACGTTACTAAACTACCCCCTTCAACCCTCTTGCTCTTTTATAGGCCTCAAAAGATATTCTTTTTCTTT
TCCGTCCTTAAATGCGTTAATGTATGCTACAACCTTTCCGTGTTTCTCGAGAAATTTCTTACATGTCTTCAGGTT
TTTTTTTTGTTTTGTTGATAGCATCAGACA

>7 c20736_g1
GTTGAAACAAAAGGTTCGCGCTACGTTTTGCCTCAAAAGGAAACGATGAAAAGCCAGCCAATCAGATAGA
GTCTTACTTTTGCAGTCTGACCAATCATATTCGTCCAGCCGGGCAACAGAATTTATTTATTGGTTCATGTTTAA
GGGGTTTTCGTCACTTCACCACGGAACGGCATCCTTGTTAGGCCATTCTGCGTCCGAAGGTCTTCTAATTCCT
CGTTTACCTCATTAATTCTAAACTTAAAACCCAAAATGCGTGAATGTATCTCAATCCACGTCGGTCAAGCTGGT
GTCCAGATCGGCAATGCCTGCTGGGAATTGTACTGTCTGGAGCATGGAATTCAGCCAGATGGTCAGATGCCT
TCAGACAAGACTATCGGAGGGGGAGATGACTCATTCAACACCTTTTTCTCTGAAACTGGTTCTGGTAAACAT
GTTCCCCGCGCTGTCTTCGTCGACTTGGAGCCCACCGTAGTTGATGAGGTTCGTACTGGAACCTACCGCCAA
CTTTTCCACCCTGAACAACTTATCACCGGCAAGGAAGATGCTGCCAACAACTACGCTCGTGGCCACTATACC
ATCGGAAAGGAGATCGTTGACCTCGTTCTCGATCGTATTCGCAAGCTCTCTGACCAATGTACCGGACTTCAA
GGATTCTTGATCTTCCACTCATTCGGTGGTGGCACCGGTTCTGGTTTCACCTCTCTTTTGATGGAACGTCTTT
CCGTCGACTATGGCAAGAAATCTAAACTGGAATTCGCCATTTACCCAGCTCCCCAAGTCTCCACCGCTGTTGT
CGAACCATACAACTCCATTTTGACCACCCATACCACCCTCGAACATTCCGATTGCGCCTTTATGGTTGATAACG
AAGCTATTTATGACATCTGCCGTCGCAACTTGGACATTGAACGCCCAACTTACACCAACCTGAACCGCTTGAT
TGGCCAAATCGTCTCCTCCATTACGGCTTCTCTTCGCTTCGATGGCGCTTTGAATGTCGATTTGACTGAATTCC

AAACCAACTTGGTTCCATATCCTCGTATCCATTTCCCCTTGGTCACCTACGCTCCAGTTATCTCGGCTGAGAAG
GCTTACCACGAACAATTGACCGTGGCCGAGATCACCAATGCTTGCTTTGAGCCGGCCAATCAGATGGTCAAA
TGCGACCCTCGCCATGGAAAGTACATGGCTTGCTGCATGTTGTACCGTGGTGACGTTGTTCCTAAGGATGTT
AACGCTGCTATTGCTACCATCAAGACCAAGCGTACCATCCAGTTCGTCGACTGGTGTCCCACTGGTTTCAAG
GTTGGTATCAACTACCAGCCACCCACTGTCGTTCCAGGCGGTGATTTGGCCAAGGTCTCGCGTGCTGTGTGC
ATGTTGTCCAACACGACCGCCATCGCTGAAGCTTGGGCTCGTCTCGATCACAAGTTCGACTTGATGTACGCC
AAGCGCGCCTTTGTTCACTGGTACGTCGGTGAGGGTATGGAGGAAGGTGAGTTCTCCGAGGCTCGTGAAG
ATCTTGCAGCCCTCGAGAAGGATTACGAGGAGGTTGGCATGGACTCCGTTGAAGGCGAAGGCGAAGGCAA
CGACGAGTATTAAAAGTATTCGATCTAGGTCTTCCTGCATTCCGAAACTTTTACGCTTCTTCAGATCTATCTCG
CGAGCTTTTTACTAGCACAGCAGAGTATGCTAAGCGCGTTCAATACAAGAATTTGCAAACCAATTACATCTGT
TTTCCATTTGAAATAAATAATTTCGTTTAGGTTTTTTGTCATTGTATGCAACACAACGTCACTAACGTTGCAAG
ACGGGTACAACTAAAATGCGTGCTTGCGTTTAAATTGTGTGGATTATCGTATACCACAATTTCATTTCTTATTTC
ACTCAACAATTATAAATACATTGTCTTTATTACATAATAAAATAAGATTTGAAATTATGGTATACGGTAATCCGCG
TAAATTAAAGATAAGTGCGCAAATCATTGTACGTACATTTTGATCACGCACGGCACAAAGTGAATGAACAGTA
AAATGTTTTTTATCAACCTGGGTGTTGGTATTTAAGAAGAGCAGTAACATCTTTTCTACCAATTTAACTTATGT
GTTTTGTCACTCCAATCATTATTCAGAGGTAAATTGGCTATTCTTGTACTTCATTAAACAAACCGCCAGTTGAA
GCAATGTGGTTAGTAAGAAAAGTAACATGTAAACATTAAAAAGCAGTGTTCAAATGTTGCTTACATTGAATTT
CATATGAAAAAGGTAGATGTAAATCAATAACCTGAAAACAATACATCAATGTTGAAAGTTCCAGCATCCAACC
ACCATGCAAAAACCCATTTGTAAAGAACATTCATTCATGTATTACCCCTCCAAAGAAACTTCACAGAACATCG
ACAAAAACTGTTTCATCTCATTCACGCAAAATGAAAATTAACTAAATAAAATCACGGATAAGTTAATCGTGTTA
TCCTATAAACAGACTCGAGAATTGCGAAGCAGGACGCGTCGGAACTTCGCACGTATTGGAAAAACGATTCG
AGCTCTCAGTAGAGTGTTTTGTAGCAATTATTTAATTTTGTGAATTAGTTTATAACTCGTCTTTGCTGGAATCAT
CAGCATCATCATCTTTGGATTGAGAACCGGCACCGGCACCTTGATAAAGTTTGGCGATAATGGGCTGAACGA
TATCTTCAGCGTCCTTCTTCTGTCTCTTGAAATCTTCGGAATCAGCCTCAGCATTGTTCTCTAACCACTTGATTT
TCTCATCAATGACACCCTCGATTTTCTCTTTCTCTGCTTCGGACAATTTTCCACCAAGCTTCTCTTTGTCGCCA
ATTTGGTTCTTTAGAGAGTATACGTAAGATTCCAGTTCGTTACGGGCCTCGACGCGCTCCTTAAGTTTCTGAT
CTTCATCTGCGAACTTCTCGGCATCACGGATCATACGCTCGATATCTTCAGGTGTCAAACGGTTCTGGTCGTT
GGTGATAGTAATCTTTTCTTTGTTGCCAGTTCCCTTATCTTCAGCCGACACTTGAAGGATACCGTTGGCGTCA
ATCTCGAATGTGACTTCGATTTGGGGAACTCCACGTGGAGCAGGGGGAATGTTGTTCAAGTCAAACTTGCC
GAGAAGATGGTTGTCCTTTGTCATTGGGCGTTCTCCCTCGAAAACCTGAATGGTGACTGTGTGTTGGTTGTC
GGCGGCGGTCGAGAAGATTTGCGATTTCTTAGTTGGGATGACAGTGTTACGAGAGATCAGCTTGGTCATGA
CTCCACCGACAGTCTCAATACCCATGGTCAAAGGGTTGACATCGAGAAGAACGATCTCACCAGTATCTTGTT
CTCCGCTCAAAACTCCGGCTTGAACAGCAGCACCATAAGCGACGGCCTCATCAGGGTTAATGCCTCGGCTG
GGTTCTTTGCCGTTGAAGAATTCCTTGACCAGTTGTTGCACCTTGGGAATACGGGTGGATCCTCCAACCAAG
ACGATTTCATCAATGTCGGTTTTTTTCATATCAGCATCTTCAATGACTTTCTGGACAGGCTTCATGGTTGAGCG
GAAGAGATCCATGTTAAGTTCCTCGAACTTGGCACGAGTCAAAGTCTCGGAGAAGTCATCACCCTCAAAGA
AGCTTTCGATTTCGATTCTGACTTGATGAGCCGAGGAAAGAGCACGCTTAGCCTTTTCAACCTCACGACGAA
GCTTCTGGACAGCACGGTTATCTTTACGGATGTCCTTGCCCTTCTTCTTCTTGTACAATTTGATGAAATGTTCC
ATAACTCGCTGATCGAAATCTTCACCTCCCAAATGAGTATCACCGTTTGTTGCGATGACCTCGAAGACACCAT
TATCGATGGTCAATAAGGATACATCGAAGGTTCCGCCTCCCAAATCGAAAACCAAGATGTTCTTTTCACCGTC
CTTCTTGTCAAGACCATAGGCAATTGCGGCAGCAGTTGGTTCGTTAATGATGCGCATCACGTTGAGTCCGGC
GATGACTCCGGCATCTTTGGTGGCTTGACGCTGAGCATCATTGAAATAGGCTGGGACAGTTACGACAGCAT
GGGTGACCTTTTTGCCAAGGTAGGCTTCTGCAACCTCTTTCATCTTGCCCAACACCATAGCTGAGATTTCTTC
AGGGGCAAATACTTTGGTTCCTTGAGAAGTCTCCACCTGGATGTAGGGCTTTGCATTCTTTTCTACCACCTTG

AAGGGGAAGAGCTTGATATCATGCTGGACAGTATGGTCAGTCCAGTCTCGGCCAATGAGACGCTTGGCATC
AAATACAGTATTTTCAGGATTGGTAGTAAGTTGGTTTTTGGCAGCATCACCAATAAGGCGTTCATTATCAGCA
GTAAAAGCAACATAGGATGGAGTAATACGGTTTCCTTGATCATTTGGGATGATTTCAACACGACCATTTTTGA
AGACACCCACACAAGAGTAGGTGGTTCCCAAATCAATACCGATTACAGTTCCAACATCCTTGTTCTTCTCTTT
TTCCTCCTTGGCCACAGCCAGAGCTACAAGAGCTAAGGTAAGCAACAAAAACTGCCCTCTGAATCTCATTTT
CTGTTTTTGAAACTGCAGTATTGAAGTAGAAAAACCTCAAACAATAAGACGAATATGGATGAGAATGACTAT
CGAAATTTGCAAGAACTATGTGTAATTCGTTTTGCCGGTCTTGAAGTTGACGTAGTAATGACGTGAATCCTTT
CTCGGTCGGAAT

>8 c16114_g1
TCCCTCTAACGCGCACAACAAAGATGAAACTCTTCTTGTCCATTCTCCTCTTTTGCTTCGTGAGCGTATCGCAT
CAAGAAAACTACGTGAGGCCTCCCGTGCCTTACGCTCGCCAATCCCGTGCTCGTTACGTTCCCCTTTTTTATT
CTAACGGTGTTCCAGCCGGCTACGATTACATCGAAGACAATAAGGCTGAAGAAATTCTACCTAGAAATAACA
TCAACGAGACGCCTCTAGGACGTTTATTCAGCAACATTCTGACACCGATCTCTAATTTAATTACGAGAACATC
TACGATCACTAGCACTGTTTTGTCCACGGTAACCACGGCAACGTACACCCAATGCCTACCTACTGGGCAGTTC
AAAATAGCTGTATTGGCCACCCCAGACGCTGATCCGCCCGTCCTTGAAGTACGTTCCACCGCAGTTTGCGCC
CGTCGTCGCCGAGATGTTGCTGAATTTTTAGTAAAAGCCGATTCTGAAATCGACATCCACCCAGCTGAACCC
CTACCGATTGAGACCAGTGCCGTGGTTGATACCCCAGCAGAACTCAGAGAGATTCAGCCAGCCCCGGCGGA
AGTGTTGTCATCCAAGTCGGATGAGCTGGTAGAAAATCCCGAGGTCCGGCAAGGAAGCAACGCGTCCATAA
AACTGGTGACTGTGTTCACCACAAGTTACACATTCGTTCCTAAAACTCTTACATCGACTGCAACGTTGGCTGC
AGATGGGGGGCTGCTGTGTCGTCCAGCCGGATACGCCATTTGTTGAAACTGATTTTTCATTTGAGTTCAGGA
TGTTCTTTGATTTTATTTAAAACATTTTCTTACAAGGAAATTTTGCCCAGTTTTCTATGCCCTTATCTCCAAATG
CATTTCGACTTGATTTTCCCTCCTTTTTTATTCCGTGATGATGAATAATACACAAGCATAAGTTATACTCCATGC
AAAAA

>9 c21178_g1
TTTTGCGCGGGAATAATAAGGGAAGTATATTATTCACGACTGGTTTACATATCGCGAAAACACTGTACAGCCG
ACATCATCACACAGTCGAAATAAATTATTTCACTAGGCAGCTAATACCTAGAACAGAATTCTAAATTGGGGAG
AGAAAGTTGATCTGTTGACATAAGCATAGGTTTCCAATATTTGGGGGGTCCGTTAGAGAGTTTGTCGGTAGT
TAGATTATGCTGCGAAAGTTGGGTGTGAGCTTTCGACGTATTTGACTCCATTGTAGGTAATGGTGTGCGTATC
CCAAGTGAACTGGACTCCGTTGGATCCCTGGACGAAGCATGGAACGTCGGGACGGGCAACGAAGCACAA
GGTGCCGATGGCGAGTTCCAGTTCGGGGCTAGCTCCCATGTTGAATTCGGAGGAAGCCTTGGTGCTTCCGT
ACAAGTCGACTGGCATGGAGAGGACGGTCGTCTTGCCAGTTTCGACGGCGTTGATGTAGCCCAAGTAGTTC
AAGTCACCATCTTCCTCTTCGTTGAACAAGTAGATCCAGCTGTGGAATCCGACGACGACGTTGTTTTCCAATC
GCTCGAACACGTTGGTGTGTTCCCATCCGCTGCTGGCAACAACTCCGGGGACGTATTCAGACCAGCGGCCG
AACCAGATGGTGCTCAAGTATTGCTTGAACTGGGCAACATCGGCGCTAGCCTCGCCTTTGGAAACGAGGAA
AGCGTGAAGCTGTTGCATGACACCAGTCTCCATGACGGCATCAATGAAAGCAACTTCCTCGGCCAACTGTTC
TGGAGTGAACGTGTCCTGGATGGTGACGTTCTTCTGGTAGTTGTTGAGAAGGGCCTGGGCGGCAGCGAAA
GTTGGCTTGGCAAAGTCGGGGATCGGGTCGCTGACAATCAATCGGCCGGGTGCGTTGTCCACCAAGCTGC
CCGCTGTGGTCTGGCCTTGGAGGTCCAATGTGAGAGTGGCTCCATTGACATCGTTGGTGTGCAGGTCTCTG
GACAGCGTCAGCAATTCATCGTCGGTGATGGGAGTGGCACGGAGACTCTTGGCGTTGGATGCACCAATGGC
CAAAACAAGGACTGAGACGATGGCGAAGAGTTTCATCTTTGTGAGAAGTTGGGTCGTGACTGGCTTTGAG
AAGCTGGTACTTTCCACTGTCCCCCAGCCAGTCACGACCCAACT

>10 c15033_g1
CGGAGCCTCAGCTGGAGCGCTAGTATACACAGGGTCCTGTGATTCGATCTAGACGCTACGGCAATGCAAAA
GGGACGCGCGCTTTGCCAGCGAAAAACCCGAGGCGAAGGACAACCCCCGCTAGAGGCCGATACAAGGCG
ATGCAAGTCACCAACGATCAGCGGCCCTACCAGTGTGCTCTACACCGTCCCGTATATAACACCTCGAATCAGA
ACTCTTGCGAACGCGCCAAATTTCCTTTTTTAAGGCACATCTCTCTACCTTCTCCCGTTCTCTTTTTTAACGGT
CTCTTGTAAATCAAACAAATCGAGATACCTCGATTCGTCTCTTTTGCACGCTCAACAACCAAAAACAACACAC
CTCAGACAAAATGAGTTCCAAGGAAGCAACGAAAGAAGTTGAAGCCGACGAAGTCATCGCCGCCGACGCC
AAGAAAGACGCAGTCAAAGGCACAAAAAGGCCAGCAGAAGACAAAAATGATGACATCAAGAAATTAAAG
AAAGCTGAAGAGAACGGTGATGACGAAGAAGCCGAAGATGAAGAAGAGGCAGAAGGCGAGGAAGATGA
GGAGGAGGAAGACCTGCCCGAAGGAGAGGAAGACTTTGATGAAGAGGGCGAAGGAGAAGGTGAAGGT
GACGATGATGATGAAGGAGAGGGAGATGATGACGATGATGATGCGTAAAAGTGTGTGACAGGAGAAATTA
CTATTTTTTTTTCCTTTTTTTTGGCTTCGGTCAGGGTCCACACTTTTTGCGCATGCGTTACGTCGCTCAGCGG
GAAAGCAGCCGGCACCGGTCGGAATTTTTTTTTGTGAATTTTTGATTTCATCTCTCTCTGTCTGTCTCTTATAC
ACTCCCCTGAATCATGATACCTTTTATTCCTACCTTTTTTTTTCGCCTCCCAATTTTTTTAATACCGAGACACAC
ACACGAAAAAAACACCTCATAATGTACGAAAAAGACTTATCAGAGCAGCCGCCCCCTCCATCTTTAATATGAT
TTATGTCATGTGTGTGTGTGTGTGAACGAGAGCGCGCGAGCCCAACGCCATCTTTTTTCTCCCTCTTAAAAAA
TTTCGGTCGCCCTGTCATTTGTATGAACGTTTTTCAGTTTGACTATGGAAAAAAAATCACTCACAAGACACAA
CCCCACCACATACAAACATTACCACCACCATCCCTGATCTGTCCAGCCCAATTTTTTTTTCCTCCTTTATTTACT
GGCGGGAGATTTTTGAATTAAAGAGGAAGAAAAATTTTTTTGAGCGGTGCCGTGTCTTTTTTTTTAAGATTT
TCTTTAGGATGTCTTTGTAAAAGGTAGGGGAGACAACCTATAGAAGAATAACACGAATCAGATACAAATAAC
AATCCTGCATAAAAGACTAGAAACCGTCTCCTCCCCCGTACATGAACGTAATGTGTGTTTAAGTTTACCGTAC
AACTTTTTTGATGGCATCGTAGAGAGGAAGAAAACAAAACAGCGATGTAAAACTTTTTGATTTCGGTAGATT
TGTTGTACTGCCGACATTTTCTTTTGACAGATTAAAAAACCAAGCTGAGCGCTCAGGATCATTATCATCAAAC
TCTCCTCGCCTACCATCTCCTCATTTACCTTTAATCACCACCAGTATCGTCGTTTATCGTCCGACTATCTCTTTAT
TTTTTGAAATTTCATACCTCGCCACCAATTTCTGGCCCAAACTCACGATGTCAATTTGAAAATAATAATAAAAA
ACAAAACAAGACAAAAGACTTGCAAAGCGTTGGGCTCGTTTTGATCGGCAGAAAACAACTAATAACGATGA
CATTGAAAGACAATGATCATGTAACATCTATTCGAATTTTACAGTGACGTAAGTACTTAATCGTTACTGGGGTT
TTACTGATCGCATTTACGAAAACGTTAGTACAAAAGAACGACTAGCTAATGTAAAATTCATTTTGCTACGGGC
TGCACAAATAGTTGCGTTCCAGTTCACTAAAACATCACTACATCAATTTGAAATGAAATGAAAAAATCATACTT
GAATTTCGTTGGCAAAACCGTTGGACCGTTGTCGATCGCCTACGTTTTTCGTGCGTCTGCCTACCCAATAAGT
TATGGGGAATGTTGTTGGGTTTCGCGAATGGTTGAATTACTTCGTCTGCAAAATCTTTCAAGTTCAAAAATCT
ATCGTGATTGTTACAGATCAAGTGTACTTTACAGCTGTGTGATAAATCAGCTGATGTGTTATCATATATTTGATC
AAAGATAAAACATTTCTTTGTTTCTCTTTTGAGTGAAAACCGTTACGTGTAATCTGGATACCGTCATACTTTGT
GTGTGTTATAACCAGAGGTATTTTTCCTGACAAACTCTCAGAAATGAAACAAGATAACAACAAGATTGAGTT
TTAAAAATTTTGAGATATATTTACAGTTTGAAATCGTCAGCCAAGTAATGTAAACAAATAAAAAGAGAGAACA
AACAAACAAGAATTTTGCCACAGACATATTTACAGTATGGTACAGAGTAAAAATTTTGTAAACAGTTACATTG
GCCAAATCAAAAAACAATAAAAAGAGAGAACAAAGACACATGAATTTTGTAACAGACATATTTACAGTGAC
AGTGCAGACCACAAGTGGTGCATCGTAGAAGTCTGTTGAGCTCTGCAATCATCATCATCTCTTTGCATGCGCA
GAAATAGCAACAAAGACATAAATGATCAAACAAGAGCTTTCAAGTGTCGAAGATGCTGATCAAAGGATGTG
ACCAGTGTCTGGATGATTGTCTGTACTCCATCGAAAAATGATTCCAAACTGCATCGATGTTTAGGCAGGAACG
ACATCGACTTCCTTCGGGGCCAGAATCAAAGGGGCTCGAGTGATGCGTGGTGGCAGGAAGATGTGAGCAT
CAGGTCCCAAAGCCTCTCGTTGAGCACGCCAGGCAGCCGAACGTTTCTTGTTGGTGTAGATGTTACGCCTTT
GTTTTGCAGTCAGCTGGGCGAACCAAGCTTCGTCGTATCCTGGCGGTGCGACAGGTGCAGCCACTGGAGC
AGGTGGAACATCGGAAGCCTTCTTCTTTCTGCGGCTACGCTTCTTCTTCACAGGAGCAACTTCAGAGGGAA

CAGTGTCCTCAGCAGCCGAAGCTACAACGGGAGGTACCACTTCATCCTCTTCAGCGGCTTCAGCGGCTTCA
GCAACCTCTGCAAGAACAGGGAGGTCTTCAATCACCTGTGCAGCATCTACCTCAACAGCCTCTAGAGCCGG
AGCAACTTCAACAACTTCGACGACAGCTGGAACTTCAACAACTTCGTCTGCAACTGGAATGACATTAGCTGC
GTTTTCAGACGGAGCATCTTCAACAACGGGGACTTCAACAGCTACATCTGGAACTTCCTCAACTGCGTCTTG
AGATGGGACGGCTGGAACAGCGACAGGAGGAACTTCCATAGCTTTCGGAGCCTCGTTGATGGGACTCTTC
CTTTTCAGCACAGGTCGAAATGGCTGGGCGTAGTGGCTTTTTGGCACAGGATTGGCGCGGAAGGGTTTCA
CCAATTCTGGAACGGGGGTTGGTTTGGAAGACTTGGCACCGGTGACAATGGGCGATGTCTTCGATTTCTGA
TTCTCCTGGATCAATTTGCCCGGGGCAGCAGCGGATGGAACAGCTGGTCTCCTGCAGTCAACCTTGGATTG
GACGTGGGCAAACTGCTTCTTCGCTCCTGGAAGTTTTGGAATTGGAGAATTCATCTTCAGATCGTTCGCTAA
GCGCTTGGAGTGATGGAGTAAATCGGCAGAGATAGAGTTTTCACAACAAGAGGTATGTAAACTCGAAAAAT
GCGATAATTTTTCGAGGGGGGGACGTTTCTTATACCCGAATTGAGGGTCATGTTACGAACTCTTAACTCAGG
ATTCTGCTTTATTTTTGCGTTTTTTATTTGCATTTTCATGTGATTACGGGACTTGAAACGTAAAATCAGTTCCAA
ATACATTGACAATTGATGTTCAACGGTTTTTAACATCGAATTAATTAAGAAAAGTATTATTTTAAGCTTAAAAT
GTACGTCGGATTCATAGCTTTATCAGCTCGAGTCGATTTCAAATTAGCGATTGCGCGGAACATGTGCTGTTGC
TATGGCGTTTAAATATAGGCTATGGTAACGGTTATTGCGTCGTGACCTTGAAACCGA

>11 c16911_g1
GGCTCTGTTTTCCAACGAACAGGGTGAGTGTGTCTCGAATTACCCTTTATCATACCGAATAGTGGTTACGTGT
TTGTGTCGATGCCTGCGAAAGTCGATTCCTGAATCCACACATTTGGACTTTTATCTTTAACCTTGATAGATAAT
CAGTATAACCCTGCTTCCGGGAGGGCTACTGTCGATTCTTTTCTCTAATATTTGACTTGTTTTTCAATTTCAATT
TCTTGTGATGGCTACTTGAGAATATACCCTATTTGATGGAGGTCGGAAATAGAATGGGTAGGAGGTAAAATTC
TTTTACTTGTAGGACCTTAGGAGCAACGTCATACGAGCCAATAATGCACCAGCTCTACACATAGGACTCAAGC
TCGAGCTATTTCGGTTTCCCTGCTCTAGGACCGTGATTAATTCAAACAAAAAAAAAAGCAAAAAGAAGTTTG
ACTTTATGCAACTATATTATTTCTCTCGTTTCATTGTCATACATCCTAATCGAATTAACTCGCCTGTCTAGATAATT
CTTTAGGGATATCAAACAGCTGATGTTGCTCTAGAAAAATGCTGACCCTTTCTTTGGATTGAATTGAAAGTAC
CGGCATGTGTCATTGCTCTTTATGTTTGATCTTGACTCTATGTCCTTGATCTTGCTGTTGCCGAGCCAGATCATA
TAGCGCCAGTAAAGCATAATTTACGGCGAAAGCCATTCGTCGTTGTCGTCGATAGTTCATGACTTTCTCTCAC
TCCAACGTTGATCAGCGTTCTGGCTGGTTAGGGTAGATACAGTTGTTGGATTTCAGATATTTAAATTTAATTAT
GTTTTAGTCGTTTCACTTTCCGAATCGATCTCCTCTCTCTTCGTTGTCGGAAACCTGTACTGTGCCCAATATCG
TCCTTAGCCAATCGACTTCGTTAGTGAAGTTAATGCCATCGATTTTTTTTTGTTTCTTTCATTTTTTTCCCCTCC
AGGTTTTATTCACGTGCATTAGGGTTTGTTGGCAGGTTAGGTTCAACGCCACCTGTCTTCAGACGAACGAAC
ATAGCTCTAGGCTTCAAAAGTTTCTCTTTACTGGTATTTAACCAAGTCAATTCTTCCTCCCCTTCCCTTTTGTAC
CTGAGCGGCTGTTCAGTCTGTTGAAAGAAGCTGCTGTCAACGTGGCCTGTAACACGCCCGTGATTTTCTTTG
CTTCATTTTTCGACAGTTGTGGAAACTAATATCCCTATCTCGAATTCACTGATTTGTTTAAACCGTACAAAGAT
AGAGTTGTGACCGGTGCTATTGCTAATCAGCTAGTAAAACCATCGCTCTTTGTGTTTTCCATTTCCTACTGTGT
TTCCCTTTGTGTGGGATAACAGCTGGTAGTGCAGGTGCTCAGGCCGTGTGCCGGTTGACCTCATTTGCTCAT
CTTTTTCTCCCTACTTTTTCATCTTCAGTCATCCTTACGGGGTTTTGATTTTTTTAAACTTAAAAATGGGTCGTC
GTAATCGTGAAATTAATACAACTGAGGAGGTTTACGAATCTCAGGGTCCTGTTGTGGATTTGGTGCACTGGC
GCGATCCGCGTAAGAGCGGAATTGTCCTGGGGGCTATCCTAGCCATCCTCCTTTCCCTGTCCGTCGTCTCGGT
CGTATCTGTCATCTCCTACTCAGCATTGGCCGTTTTGTCGGGCACCCTTTCTTTCCGACTCTACAAGAATGTGT
TGCAGGCCGTTCAAAAGACTCAAGATGGACATCCTTTCAAGGAATACTTGGAAAAAGATGTTACCGTCACA
ACGGAAAAGGTGCACGAAGTAGCTGATTTGACTGCAGCCAAATTGAATGCAACTTTAGTCGAACTGCGCCG
ATTGTTCCTGGTTGAAGATTTGGTGGATTCCGTCAAGTTCGCACTCGTCTTGTGGGCCCTCACCTACGTTGGT
TCCATGTTTAACGGTCTTACACTGGTCATCTTGGGTGTGATTGGTCTTTTTACTTTCCCCAAGGTGTACGAAA

CACACCAGGAAAAAATCGATCAGAATGTTGATCTCGTGAAAGGCAAAATCAACGAAATTGTTGACAAGGTT
CAGGCTGTGTTGCCAATCGGCAAGAAGGCCAAGAGCCAATAAAACTGAAAATTTAGAATAGCGAGGCTTGT
GTTTCGGCCCCGTCCTCGGTGTGCTTGGGTCAGTCGGGACTCGGTAGACTTGACGAGAAGAAACCTTTACT
AGTTGATTTTTCTCCTTTCTCTTAAATTGGTTGTGGCTGCTTTATCTCTTTAGACCTTATTTGCGAGAAGAGAA
ATAACTGGTCATACATTTTCCTTTCTTCCTATCCCCCCCTATACTATACCTTGGCCTGTAGATGAACAACCATAAC
CGTCATTTACATCGGGCGATCGCACTTGTTGTGTGCTCCATAGCAAATTTCTTTCCTATCTCCATCACCTAGGG
ATTTTGAAATTCGGAGGGCGTCATATGTTTGTCAAAGACAATTGTTGGGATTTCAAGGGCTAAAAAGAAAG
ATACTGAACTAGCGAAATGCCCTTGGCTAGAATTTGAGTTTGCTCACCTATAATATCCTCTTATAACTACTCTTT
GTGTTGTCATGTAACCGATTCTCTCAACAAGTCTAAAAACATCATTTTATTGTCTTGTGAATCAGTGTGTGGTT
TGTTGACACGCTAATTTTGTCAATCTCTCGAATCTCAGTGTGAGAAGAGCTGTTGTAGTGTTTCGCTGTCCCG
GCATGTCGTCAGTCTCTTACTCGATACAGAATTTCAGCCAGAAAGTTATACGCGCTTCTCATCCTGCTTTGGT
GGCACTCATGAGTTCCCTTTGTCAATTCTCTACTCATATTTCAGATAAAATTTATTGTTATATTTTTTATTCTTTAA
GAGCTGAAGGTATAAAGCCTCTTTTCTACTAATAATATATAGTACTGTGCTCCTTGTCTGCTCTGTTCCCACCTA
TTCCCATCACACTTTGGAAAATAAATATTCAACGTTCAACCATAAAAAAAAAAAAAAAAAAAAA

>12 c21985_g2
CTACGGAGCTCACTTGGTCAGAAGTGCCTGTGACTACCAAACCACATCGTTTAGTTTGTCAAATCTCAAAGT
ATTTTCTTTTAGCGTGGAAGAAAATGTCGTGGTGGTCCAATGGAATTCTTCTGATGGGCCTATTTTTCACCGC
CAGCCAAGCTTATGGTCCACCGAGACAACATCAGTACGTCGTCCAAGATGCAGCATATTACAGACGAGGTTA
CCCACAACCACCATACCCACAACCACCATACACCCAACCGCCATACCCACAACCACCATACACCCAACCGCCA
TACACACAACCAACGGCACCAACTTACGAGCCGGTCCACTACTGCCCAAATGTTGCTGGATTAGAGACTCGA
TGCCGTCCTACTAAGGATTGTGCAGTATGGTATGACGAAACCCTCGCCACGCCATACACAGCTTGCCAATTGA
GTGATGGAAATGCAGGATCCTGCTGTCCCGACATTCCTTCTAACACTAAGGGTACGCCATTCAAAGAGCCAC
CTGCATGCCAGGGCGAAATTCAACTGGACGACCACGTTGGATGTCTCGACGTATTTTCTGTGAATGCAGCTG
CTGACGCTGGAGAATTAGAGCTCAGATTACACAAAGAAACAGCCAAACGTCTCAAGGATAGCGATATTGTC
GTCCAAGCCGCTTCACCCCGTGCTACACATGCCCGTTTCTCCAACCCATCAGATCACGCTCGCGTAGTAAACA
CAGAAGCATTCGTCGGTGTCCATACTGCCGTCGTTCTCGTCAAAAGATTCAAAATCAAAACCGAACAGGTTG
GCTGCGCTTTGCACAAATTCAAGCTAACTGACACCGATCTGGATAAAAAATGTACCTCCATTAGCAAAGCCC
ATTGTACTGAGCACCAATTGAATTCCCCCTACCGAACGATTGACGGTTCTTGTAATAACGAAAGACACTTCGT
TTGGGGCCGTGCAGCCACTCAATACCATCGCATGCTCGCTTCTGATTACGCTGACGGTGTTTGGGCTCCGCG
TACCGGCAAGAACGGAATAGAACTTCCCAGTGCCCGTTTGGTATCTACTACCTTGGTTCGCGATGAAGACAA
GCCTAGCGAGTCGACCACTATCTGGTTCATGCAGTACGGACAATTTCTAGACCACGATATCGTATCCACTCCA
GAAATGACGAACGAAGATGGAACACCAGTTACCTGTTGTAGCGAGGATGGAAAACATCTTGAAAACGAGG
ATCGTAGTCACGGCAAATGCCTTCCCATCGACGTCCCAACCAACGATCCATTTTTCGCCAAATTTGGCCGAAC
TTGCATCCAGTTTGTCCGAGCTAGCCTTGCGTGCAGAACCGATCACCAACTGGGTCATGCTACACAGCTCAA
CGACAACACCCATTTCATTGATTTATCGCTCGTCTATGGCTCTAACGATAAATTAGCAAGTGAGCTTCGGACA
CGAGTTAACGGTGAATTGAATGTCACGCTGAAACAAGGCAGAGGTTTCCACAAGTTTGACTTGCTACCACC
AAACGAAGGTAGTCCTCTGCATGCACCTTGCACCTTACCCAGAGAAGTGAGTGGTGTTGAACCACCGTCAA
ATGTCAGATGCTTCAAAGCCGGCGATGGCCGTCCCGACGTAACCCCTAATATGGCCACCTCGCAAATTATCTT
CTTGCGAGAACACAACAGACTTGCCAAAGAATTGGCACAGTTGAATCCATCGTGGGATGATGAGCGTCTCT
ACCAGGAAGCCCGCCGCATTTTGATTGCCCAGGCTCAACACATTACCTACAACGAATGGCTACCTATCCTTCT
CGGCCGCTCGAAAATGGCCCAACTTGGCCTGTTGCCATTGCAGAGTGGTTTCAGCACAGATTACGATGATCA
CCTCAACCCCAGCATCTTGAGCGAATTTGTTGCCGGTGCCTTCCGCTTCGGTCACTCTATGGTGCAAGGAAA
AGCTCTAATGGTCAACCAACAACGGTCCGTTGAGGAAGATATTCAGTTACGTCATCACTTTTTCAAACCGCA

AACGCTCTACACTCCTGGCAATCTTGATAAATACCTCATCGGTTTGGCTACTCAACCTACTCAGAAAGTGGAT
CTCTCCTTCACAAAAGAGCTCACTGAGCACTTGTTCGAAGAAACCGGCATGGGTTTCGGTTTGGATTTGGT
GGCTCTCAACATTCAGCGTGGGCGCGATTTCGGACTTGGAAGCTATAATGATTACCGTGAACTTTGTGGTATT
GGCCGAGTCACAATTTACGATGATCTCGCCGATTTAATTGATCCTGTGGTTATCGAGAAACTCAAAGAAGTTT
ACACTAACGTGAACGATATTGACCTGTTTGTTGGAGGCATTAGCGAGACAAAAGCAGAAGGCGCTTATGTA
GGCCCCACCTTCCAATGTATTCTGGCCGACCAGTTCTTGAAACTGAAGCGAGGAGATCGTCACTTCTACGAC
TTGGGCGGACAACCTAGTTCATTCACCGAAGACCAATTGAACGAAATCCGCAAGACAAGTTTGGCCCGTAT
CGTTTGCGACAACAGCCAAGTTAACGACATTCAACCCATGGTTTTCAAAATGCCATCTACCATCAATCCTGTC
GTGAGTTGCCAATCTTCCTCTATTCCCCGCATGAGCCTTCTCCCATGGCAAGAAGCTACTAAATCCAATTCCAC
TGGCTACTACGATGAGTATTACTAGAACGTTCTTTCTGAAATTCGGATTTCGCATGGCCAAATGCTGATAGCAT
TAACACTCTTGTACTAGACTAGGAAAATATACATTGAACAAATTGGAAAAAAAAAA

>13 c16323_g1
TCGACGATTGTTAGTGGAAGAACGTTGGGTTTGTCATTCAGTCCACAGCTTTCGTCTTGTCTGGTCCTTCTTG
CAAGTACCTGACTTCGCGGCTATTTCAGTCTTCTTTACAAGTCCATTTATCCCATTAATTCATCAACATGCGTGA
AATTGTCCATCTTCAAGCCGGTCAGTGCGGAAACCAGATCGGTTCTAAGTTCTGGGAGATCATTTCCGACGA
ACATGGAGTAGATCCAGAGGGCAAATACATTGGTGACAGCAACCTTCAACTTGAAAGAATCAATGTTTACTA
CAATGAGGCTTCAAGCGGAAAGTATGTCCCCCGTGCTGTTCTGGTTGACTTGGAGCCAGGTACCATGGACT
CCGTTCGCTCTGGACCATATGGCAGGATTTTCCGCCCAGACAACTTTGTGTTCGGTCAGAGTGGAGCTGGTA
ACAACTGGGCCAAGGGTCACTACACCGAGGGTGCTGAGTTGGTCGACTCAGTCCTAGATGTTGTCCGTAAA
GAAGCTGAAAATTGTGACTGTCTCCAGGGATTCCAACTTGCTCATTCCTTGGGAGGAGGTACCGGATCTGG
CATGGGAACCCTGCTCATTTCCAAGATCCGAGAGGAATACCCAGACCGCATCATGAACACATTCTCAGTTGT
TCCCTCTCCCAAAGTGTCCGATACTGTTGTGGAACCTTACAATGCAACCCTCTCCGTTCACCAGCTGGTTGAA
AACACCGACGAAACCTTCTGTATCGACAACGAAGCGCTTTATGACATCTGTTTCCGCACCCTTAAATTGACCT
CTCCTACTTATGGCGACCTCAACCATTTAGTTTCGGTCACCATGTCTGGAGTCACTACCTGCCTCCGATTCCCA
GGTCAGCTCAACGCTGATTTGCGTAAGTTGGCTGTCAACATGGTGCCCTTCCCCCGTCTTCACTTCTTTATGC
CCGGATTTGCTCCTCTGACTGCCCGTGGATCTCAGCAGTACCGTGCACTTTCCGTCCCAGAACTCACCCAAC
AGATGTTCGATGCCAAGAACATGATGGCCGCCTGCGATCCTCGCCACGGCCGTTACTTGACCGTCGCCGCCA
TCTTCCGTGGACGTATGTCCATGAAGGAGGTCGACGAACAAATGTTGAACGTCCAGAACAAGAACTCGTCG
TACTTCGTCGAATGGATTCCTAACAACGTCAAGACAGCCGTTTGTGACATTCCACCAAGGGGTCTCAAGATG
GCCGCCACATTCATTGGCAACTCGACTGCTATCCAAGAGATCTTCAAGCGCATTTCCGAGCAGTTCACTGCC
ATGTTTAGGCGCAAGGCTTTCCTCCATTGGTATACTGGTGAGGGTATGGACGAGATGGAGTTCACTGAAGCA
GAATCCAACATGAACGACCTTGTTTCCGAATATCAACAATACCAGGAAGCTACTGTCGATGATGGTGAATTTG
TGGAGGATGACAACCCAGAGGAGGAGACCGCCTAAGGAGGAGCAAGTTTCAATGCGTGAACGAAACTTG
GAAGTTTGATTGGGGAATTGCCAGAGTGGACGCAGTAATTTGGGGAGAATGTTATTAGTTGCACCAATTGA
AACCAGTTGCTGTATCCTTCGTGAAACAGTTGAATGCTAAAATTTTAAAATTTAATTGGCAAAATTTCTTAACA
ATTCAATGTTATGAAAATTTTATTAGTAATATTTTGGAGTATTGTTGAAGCATTCCGGTGCCAAATAATTCGACA
ACATTGCTTGCTTCTTTTGGGTTTTAACCGCCACCCTTCTGCTCCATTTTTCTGCGTCGAAATTTTGGAATTTC
TGCGAGTTGCATCCTTATGAACAATATGAATAAAATTTTGGAAACCCAAAAAAAAAAAAAAAAAAAAAA

>14 c14544_g1
TGTCGTTGGTCCGGATCAATTCGACAACTATTCGAGACCCATCAGTCTTCGAAGAGCTACCGAATTGATCCG
GACCAACGACATGGCCATCCAGAACAACATTGCTGTCTCTATGCTAGTTTTAGCTGTATGCTTGGCATCAGCT
ATTGCCTCCACCCCCACCAGGGATCCAAAAGAGCTTCTGATTTTCCCGCATTCCTACTCGCCTTTTTATCGTGT

GCCGTCCATGCAGTCTCGTTATGGCCCGACCCAACGCCAAACCAACTCACCAATTAACCCCATGATTGGCCA
ACAAGGTCTGGGTCCATTTTTCGGCTATTTTCCAAATCTTTTGACTCAACCATGCCCGGCTTGCCCTGATTGC
GAAATCTGTCCCGCCTGCGAGGTCTGCCCAGTAGTCCCGGAAATGCCTCCTCCTGGAGAAATTACAACATGC
GGTGAAAACTATGAGGCACCTATGTCGGCATCGCTTAATGGCACTATCAAGCTGAACATTGCCCCTACCGGC
ACTTCTAACGCGAAAATTTGCCAAGTTGGTTTGACGGCTTTTTATCCAAATACCAAAGTCAAGATCAACTGC
GGCACGTTAGGATCTGGCGTCACATTGAACGCTGCTCCGCTCTCCCAACCAGTTACAGTAGCTGAGGCTAAC
AAAGATTACCTTTCCAAGGATAACTTTCTCTTCGTGATTTTCAACAACGGTGCAAGCCCGAGTTCAACTATTG
CTGCCGAATGCACTTGGACAAATGTTCCGGCGTAATACGTGAACATTGATTTGAAGTGGCTTTTCAGCCATCT
ACACCCTTTTCAAAGTAAACATGGACACATCGTAGCACTTCTCGAAACATTTGACATGATGAATTGCTTTACA
GGATATATTCAGTTTTCTGCTTAACGTATGAAAAAGAGTTTTGATTGTTTGTGCACTTTATGAGGAATTTTCGG
GAATGATGCAACACACTTTGGTTTTTTATGGAATCATGCTTTTTCGTCTGAATATTTAATACAAGCATTTGAAA
AAAA

>15 c31684_g1
TGGTAGTTATAATAATTTAAATGACACTGAATCAAATATTGATTGTCTTACCACCAACGGTAAGCTTCCGTCTTA
AAGTGATGTCGCGTGTTGAAACGTTTTGCGGTAGTCAGACGTCACGATCGCGGAATTTCCAACGAACCCCA
CCCATTCGTCAGCTCCCTAGTTTGCTGTTGCCATACAAATACAAGTTCAGCAATGCCCTTGCGTTGAACGTTG
TTTTAGATTCGTGCTACCAATCGAATTCTCGTTTTTCCGCAGTGCCAGTGAACATATGACGGGATGGCAAAAA
GGTGTGGTCCATCCAAAACAATGAAAGAGACAACTACCTGTAAAATTGAATCTTGTCAAACAGGAATTCAAG
GTTATTAAGATCTTCTCGTGAAGGATTAGCTTTGACCGTGAGTCAAACAAACAAAAAAAACGAACACGCGG
AAGTTAACTGCAGTTCAAGTAAAGGGAGTAGCCACTTGCCCACTGGCCAATTTGAACGATATAAAATGAGG
ACCGAAGTTCGATATGGGCACTTGTTCACCGCACCTTCTGTCGATTACAAGCGTTCAACATGAAATTCCTGAT
TTTGAGCGTCTTCTTGGCTTGCGCCTCGGCCCAGTACATCCCGTACAGCCAATGGTCGCCGTACAATCCCCA
GTTTGTGAACGACCTGCACTCGAAGAGCGTCGATTTGAACAAGGACGGCCAACCTGACGTCCCAGTCTACT
CAGCTGTTCCTGCATTCGTGCCCAGCTACTACCCTTATGCGGATTTGGCTTCTCGCACTGGATACCCATTCCCT
TACGGCGCTCCCTTCACCTACCCTTTCGTCCAGGCCGCCACTTCTGTTGTCGCCAATGCTGACCCTGCTACCA
CCAAAACCACACGCGCTAAGCGCCAAGTGCCTCTTCTGCCCCACAAACCATCGGAATTTCTCAATGATTTCG
CATACAGCAGCGTCGATTTGAACCAAGATGGACAGCCCGATAACGCCGTATTCACACGCACAGTTGCCCCAT
TCACTGGTGCCCGATTCACCTATCCGTCTTACCCCGGATACTACCCCGGATACTATCCCAACGTCTACCCATTT
GGCAACGGATTCCTCGTCTAAACATGTCACCAAGCAGTCCACAGAAACCACCCGAATGAAGCGCTGTTATCT
AATTTTTCTCGTCCTATGAAATATGTTGCTTGTTGCTGTTATAATTCTTCTCCTAGCGAATACAAATTTTGATTGC
ACGTCGGTCGACTCACACTTA

>16 c9802_g1
GTTAGAAACCCTTCGGGTAATTCAAACAACCGTGTATTTAAAATCTCATTGCTTTCTTTTTCAATTTATTTATTA
TTATTTCTATCATCCGTGATTCTTTTTTTTTTACTAAAACTATATGAATGATTTTTTTTTTTCTTTTTCGCAAAATT
CGTTTAACTTCTACTGTTTCCGTCACGGGACAAAATAAAAAAGAATGAATTGCTCACACATAGAATAACAACA
CCAAACATTTTCTTTTTTGAACATATTTGCAGTTGAACATGCGGGGAGTGTCGGCGATTTATTCCATGCGTGT
TTGTCAGTAAAAATGTCAAAAAAAAAAAAAATACCGTAAAAAAGTATGTGTTGAAAAATCATAAAATCAGTGA
AGCTTCACGACGACGAAACGGGTTGTGAGGAGTCTGACATGTCGGTATAATAACACATGGAACAAACAAAA
CAAAACCCAAAGAGAGAGACAGAAAAGATGTATGGGATGGGAGACGTAAACAAAAAAAAAACTAAAAAA
GATAATGGTTTTGAATAAGGCAATTGGGACCCAAACATGGATGTCACACACGATACATTTTTATCATCAATTTC
GTCAAGGAAAATGAAAATAAACTTAAAAAAAAAAAAAATGCAAGAGACTGTATCGTTTCGACTGTCTCGATGT
CGCTTTTCTCTTTTTCCACCTGCGAGTTGAATGTTGTTTGACGTTTTAGACTGTCTGCACTTGATTGTTATGCG

TCAATTTCACGTTGGAAAAACATCCGCCAACAGCGTTGGCAACCGGTTCCAACGTCGTCTGCACCAAAGCG
ACCCAGACCCTTCGAATGTAGAAGAGGAAGACATCCATGATACGCAGAATGGGCGTGGCGAACCAAACGG
AGAAGAAGGTGATGAGCGAGAAGGTAATACCCCAAACGAGGGCCAATGGTAAGGCGAGGATGGCCGATA
GCAGACGATAAATCCAGAAGCGCGTACCGGTGAAGAGGATGAAAGCCGAGCGCCAGATCGGGTCGAACCC
ATGGGCCACATCCGGTTCAGCCAAGATGTCTTCAAAGTTCAAGTTGATGGTGTCGTTGATGTGCTTCTCGTC
ACGGTCGAGAACGTTCAGACCGACGGTGAAAGTCTGGACGCAGCTTTGTGTCCGTCGATGGCAGGCCGTC
TTCTCCTTCTTTTCTTTCTTTTCTTTCTTCTCTTTCTTTTCCTTCTTCTTCTTCTTCTTGTCGCCGCTTTCATCATC
GCCGTCCTTCTTTTCTTCCTCAATCTTTTCGGGATTAGAGTCGTCCTTTTCACCCTCCGATGTGGTGGATGCCT
TTTCCTTCATCTCAATGACATCCTTTTCGGGAGTCTCGGCCTTCTCGTGAGCCTCATCGTCCAGCAATGGCATA
GTGGACTCGTTCAAGTTCAATTTCGAGCCGGAGCGGGAAGCGGATTTGGTTTCGGCCATTTTCTTTTTGGA
AATTAACTTATTTAGTTGATGATTGGAACGAAGCTGATTTAGTAGACGATTAGACGAGTTACAGAGAGACAC
GTTCGTACGCTTCGAATGAAGAGACGCTTCTGAAGGTTTCGGCTTGTAAGTCCGCTAACCAAAAGGATCTCA
AACTATATGGTAACACGTTACTTAGTTTTTTAAG

>17 c31718_g1
TTTTTTTTTTAAGATAACAAAAAAATGTATTGCGTGGCCGAAAAGAGACATAAGAGAATGTTACGCAAATGC
TTTTTTCTTTTCATGTGTGGTGTCAGTGTGGTGTTTGCGTGTGTAATAATAACTGGGGTTTTTTCAAATGGGG
AAACCTGCTGTAGAGGACAAAAGAGAGAGGGGAAGAGCAGCAATACGGTTGGGGTCGTCAGAGACCCCT
ACATGTTTGTGTATGCGTGAACATTTATTGCAAAAGGGTCTGGCTGTATGTACATCGCCATGTGAACCGTTGC
ACAGCATTACGGCCATTAATGATTGAGTTGTTTTTTTCGTAATTTAAATGCTTATCCGAAAATAAACGAGCTGT
TGTAAAAAAAATAATAAAATAAACAAGAAGTTGTTCATTCCCTTAGAGGGGCACTTCCGGGTTGCGGGAGC
GGACAAAGTTCAGCAACTGTTCGCTGTCCTCGCAGATGAAGGGTTTCTCGTGGCTGCAAGCAACGTCGTGC
CACTTGATGCCATCGTTGTAGAAGTTGTTCAAGATGGCCAAGCAAGGCTCACCTTCGTCATCTTCGCGGTTG
TCGGGTTGAGGGCGACCATCTCCACCGGTGTTGGACCAATCTCCCAAAGCACGTTGGTCAGTAGGTGGGAC
TTTAGCCTCGGATCCGGACCAGAACCATCCGTTGACAATGGTTGGCTGCAAATCAGCGCGGTTGCATCCGTC
GAAGTCACATTTGCGGCCGCTAGTCCAGATGTACTTGACGCCATAGCGAGCCAACTGTTGCTTGATGAATTC
GTTCTCTTGGAGAGTCTCGAGGCTGACGGCGTCCATGCAGTGGCGACGGCAGACGTTACGGGCGTCGAGC
CAGTCGACTTCCAAAGAGGCAGTGGCCGGATGCAGCCAGCTGAAGAAATAGTTGTGGGTGTTGCCAAGGG
GATCCGTCCATAGGGCATGTTTAACACGTTTGGCACAAGACTTGGGGTCGGGGAGAGCCAAGACTCGTTG
GGCAGTGACGGCGGTCAAGCCGCAAACGACCATGAACAATGTCTGCAGCAACATTCTTTGATCGTGAAAAA
GTGTCTTCAAAAAATCAACAAGTTTTGTTTTTGTTCTCGCTTATCTTGTGCAGCACTGGCTTTTGCTGCTGCT
GTCTGAGGTATGATCGGCAATCTACTCGGTCCGCAGGTATTTATGCTCGCACCGGGATGGGACGGGAATGTT
CGTGAGGTTTTGTGTGTGTGAGTTGCGCGTTTCGGTTAAGG
>18 c13264_g1
GAAAGCGTGAGGCGTGACTATTATTCTTTATTCCTCAGTTGGCAGTTTGAAATCTTACGTTATTCTGATTTTAA
TCGCAAGAAAAACATGTCAGATGCATTAATAATGTGCTATTTCACCTTTCTAATGTTCATTTTATCTGCAATAAC
GAGCATGTTCTTTGTAACCTTAAGTTTGCAGTAATCACACACGTGATAGGTATCGTAAATTTTTTGTTTTCACT
GTACTGCTTAATTGGAGATCTTTCTTTTCCAGTATAAACATTAGACCTAGCCGTTTCATCGCCATATCAATTATC
AAGACGTCATCATCGACATTGATGCCATTGCAAAATATACAAAACTGTTCATTTATTTTTTTTCCTTAGAAAGT
TAGGATTTTATAATGATTTACTACGAAAAAAACGATCATTTGTGTCTCCCATCACAAGCGCTTGTCGAATACTA
CGCTGTATGCTGAAAATTTTCCTTCTAGATCGAATAATGACTCAAGATTGAAGAGCAGTAACATGATTGCATA
AACTGGGATGTCAAACAGGACGAGGAATGAAAGTATCCAACAATTACCGCTATACAACTTGACAACACCACA
ACAATCGAGAAGGGGGAAGTAACATGTGGGGCCGGAACCAAAACAACTGACTCGGATATGGGACTTTACA
TCATTCCACCCATGCCTCCCATGCCACCCATACCGCCCATGCCTCCCATACCACCCATGCCTGCTTCGGCCTTG

TCTTCTTTAGGGATTTCGCAAACGACACATTCAGCAGTCGTGAGGAGAGAAGCAACTCCAGCGGCGTCAGT
CAGAGATGTACGTACTACTTTGGTCGGGTCAATAATACCGCGTTCAATCATATTGACGTACTCATTTCGGAGC
GCATCATAACCGATAGACGCTTCCGAATCCATAACCTTGGCGACGACGACTGAAGCATCAACCCCAGCATTC
CTGGCAATAGTCATGCAAGGCATCTTCAATGCGCGACGAATAATGTCAATGCCAACTTTTTGATCCTCATTTG
CGAAGGGTAATTTGTCCAAAGCTGGGATACAACGAAGGAGGGCAGTACCACCACCGGGAACGATGCCTTC
CTCAACGGCAGCTCGTGTGGCGCACAAGGCATCGTTGACACGATCCTTTTTCTCGTTTACTTCTACTTCGCTT
GAACCACCGATCTTAAGAACAGCTACTCCAGACGCGAGTTTAGCTAGCCGTTCTTGCAATTTTTCCTTCTCAT
ACTCGGACGTGGTTTCAGCGATTTGATCCTTGATCATAGTAACACGCTGGGCAATGTCTTCTTTTCGACCTTT
TCCTCTTAACAAAAGGGTGTCATCTTTGGTGACAACAACTTCTCCAACCTGGCCAAAGTCATGCAACTGAAT
ATCTTCGAGTTTGACGAGATTTGCCTCATCGCCGAAGACCAGGCCACCAGTAGCATTGGCCATGTCATGAAG
GGTGTTTTTGCGGTTGTCCCCAAAACCAGGTGCTTTAACAGCGACAACTTGGAGTCCAATTTTCAACCTGTT
GACAACCAGAGTACTAAGAGCTTCGCCATCGATGTCTTCCGCAATAATGACTAGAGGTTTGCGGTGTTGGTT
TGCAAGCTCTAAAGCAGGAATGATAGACTGGATTGATGAGATTTTCTTCTCGCTCAGTAGCACTAACGCATCC
TGATATTCGACTTTGGCGCCCTTTGCTGTGTTGATGAAGTACGGCGAAATATAACCGCGATCGAATTTCATGC
CTTCTATCACTTCTAGTTCATCAATCAAAGTTTTGCCATCTTTAACAGTGATAACTCCTTCCCGTCCCACTTTGT
TCATTGCTTGGGAAATCAATTTTCCTACTGATTCATCACCATTAGCTGAGATCGTGGCAACCTGAGCAATTTCC
TCTGGAGTGGTAACTTGTTTTGACATAGCTCGCAAATTACCAATTACTGAATCAACAGCAAGCATGACACCAC
GGCGTACTTCAACAGGGTTAGCACCCTTAGTAATCTTTTCAAAACCTTCCTTTGCGATTGAGCGGGCCAAAA
CTGTTGCAGCAGTTGTTCCATCACCAGCTGATTCATTTGTGTTATTCGCAACATCTTGAACTAACTTAGCACCA
ATGTTCTGGAACTTGTCCTTCAGTTCAATACCCTTTGCTACCGTCACACCATCCTTTGTAATTTTTGGTGATCCC
CATGATTGCTCCAGAATAACATTTCGTCCCTTTGGCCCCATTGTGACTGCGACAGCATCAGCTAGGACATCTA
CTCCTTGCAACATGGCTGCTCTGACTTCGGCTCCAAAACGCACATCTTTGGCATAAGCTCTGTACGATTGGG
GTGCAATTTGACGAAGAGCTGCTGTTCTCAGAAGACTTGGTAGCCGATACATTTTTTAAGTGATTTAAAGTA
AATTTGTCTGAGTAGATCCCACGTGCAACGCTTGCCGACGATAGTACGACTGAATGTGAAACAGAAGACGA
GCGGTACCGTCCCTAAAATAAAAGAGCCTCTCGCCGTCTCGCGGAAGCTTCGAGATCACCCCTTACCTCACC
ACTTCACCAGTCAGTCACCATAATAATGTTATGGTTATTTATTTATAACTGAATCCCGATAGCGATAAGAAATTT
CTGTTTGTAAAATATGTGTCTTTAGTCGTGGGCTTGTGGGTTTAACCGGTGTCCTTCTTTGTTCTAGAAGGAT
CTTCTAATCGCGAAAAGAAGCATTTGGATTCAAATATTGTGTTAAATCATTATCGGAAAATGTATTTAAGATTA
TTAATTCAACAATGATGGAACCACGTGGCTTACGTAAGTTAAACCAGGCGAGCAAGCAGACGCTGTTCTTCG
TTTTTTCCAGTCCGCCAGAGCTAGTCTTGCCGGTTTAGAAGAAGCACGCAGTTTTTCTAACTCAGTAGTTAA
CAAACCAAAATGGCGAACGCACTCAAACGATTTATTCCCATGTTTGATCGTGTTTTGATCGAAAGGGCTGAG
GCCATGACTAAAACTCGAGGTGGAATTGTTATTCCTGAGAAAGCCCAACAAAAAGTGTTGAAAGGCATGGT
TGTTGCAGTTGGACCGGGATCCAGAACAGAGAAAGGTGATCTAGTTCCATTGGCAGTGAAGGTTGGTGAC
CATGTTTTACTTCCCGAGTATGGTGGCACTAAAGTTGAGATTGAAGAAAAAGAATACCACTTATTCCGTGAA
AGTGACTTGTTGGCCAAGATTGAACAGTAATTCATATAAAGCATTTCCCCCTTAAAAAAAGAAACAAACTGA
GTGAATGGGAAGATTTGAATGTCAGCTTGTATGGTAGTCATTTATATCATGTGCAGTTTAGGAGATGAAATAA
ATCCACTTTTTGTTACATTTT

>19 c31673_g1
AGTCTGTCATGGAAAAAGAATTGCATGATTCAATGACAGATGGTGTTGTAAACCTTTATAGTTTCCATGGTTT
CCATGATGTCTTTCTTTCTGAAGATACCTTTTCTAGAAACATTGCATCTAATTTCAATGATTCATGCTCATTTAG
TGTAAAAAATTACGATGAGTGTTGTAGAAGCTTTGGTTGCTTTTGATGAACCGGTATTTATTCTCTATGCCTGC
CATATTGTTGAGAATTTTTGCCTGTTTTAAAGGCCACAAGCCCCACAGGATACCAAAAAATTCTCAGTTGTCT
TTATTGATTTCTTCCCCACCCAAAGTAGTAAAAACAAAGATCCGGCACGTATGGAATAACGTAACCATGATTT

CAGGATTGCAATGGCGCTCCATCCGATTAAATTCTAAAGATTTCACTCGTATCAAGCATTTCACTCTTATATTTT
TATCAAAATGTTTTACCCTCTACAACCGTCCTTCTTCCCACAAAGCTTCTCAAACCTTCTGCGTCAAACTGGGT
GCCTTTATCTATTTCGAATCCTCAGCCAAAGATGGCGTAGACTGGTGACATAACGTGTTCATCTACAGTTTCG
CAAACGTCAATGGCCCGACTGTTTAAAGTCACAAATTAAGATTTTTCTGATCGAAGAAAATCCAAGAAACTT
ACGAACGAAAAGTTAATATAAATCCTGAATTGTTCATTCGTGAAAAAATCACTTATTCTATTCAGAAAGACGC
CGTTTTTTTTTGTGTTTCTAACGAATAGCATTCCCATTATACATAGTGTATAATGGCTAGTTGGTTTAGGGTAGG
TAGGTTCCCCTTCGCAGTGCATATAGATTTCCCCATCCTATTTTCCTGTGTTAACGTTTCGTGTGTGTAGATGTG
TGTGTGTGTGTTACGGTGGGGCTATATAAAATCTTGCCTCAAGGTTTCCCCAAAGGTTCGTAGGCCAATAGG
GGCTCAGTTAGTGTGCCACGCAAGAGCCCTTTCTGTAAGAAGAAAGAGGATTAGCCGCCCTCTAGGCGCCA
GAATTGAAAATTCAAAAAAGCAAATTCTCATTGGCTGCCCCGTCCCTAGTCCATTGCCATATTCCCCACTCTTT
GGAGGTTCTTATACGCTGGCACACAGACTGTTCCTGTGGACTTTTGTATGCTTCGAGATAGCCCTCGTTGTAC
TGAACTCACGGCTTAAAGGTTTTTTACGAATTTACGAATTTGCTGCTTTTCGAATTATCGACGTTTCAGAAAA
TGCCACGCGCCAAGGCTGATGCTAACAAACCCCGTGGTCGTATGACGGCTTATGCTTTTTTTGTCCAGACTT
GCCGCGAGGAGCACAAGAAGAAGCATCCCGATGAGAATGTTGTGTTTTCCGAGTTCTCTAAGAAATGTGCT
GAACGATGGAAGACTATGAGTGACAAGGAAAAGAAGAGATTCCAAGAAATGGCTGAGCGTGACAAAGTTC
GCTTTGATGATGAAATGAGGCATTATGAGCCTGCTGAAAAAGGTGCAGGAAGGGGACGTAAACGCAAGCA
AGCCAAGGATCCCAATGCTCCCAAGCGCTCTCTATCAGCATTCTTCTGGTTTTGCAATGATGAACGTGGAAAT
GTGAAAGCTGCTCACCCAGAGTACACTGTTGGAGATATTGCCAAAGATCTTGGCAGGCAGTGGGGTGAGG
TAGATGAAGCTACCAAATCCAAATATGAAGCCATGGCTGAGAAGGATAAGGCTCGCTATGAAAGAGAAAATA
ATGCCTACAAGAAAAAGCTTAAGGGTGAGGCTGACGAAGAAGATGAAGATGATGATGACGATGATGATGA
GTAAACATCTTCGTTAGACTTACCTCTGCTCCTTCCCTCCTCAAACCCCCGGCTCTTTTCATCCCCGTCGCATTT
TTTTTTCTTTTTTTACTCCAGTTAGACATGTGTTCGCCAGAGTATCTTCATGTTCGTTTTATTTGCATCTTCATCG
TATCATCTTCATTTCTTCCTCCCCAAAACTGCTGACTGGTGTACATATCGAGGAAGACAGACACAACAGATAA
TCGCTCGCAGCTCCCATAACCCCCTTGCCCATCCACATATTTTGAAAAGTTCTTGCAATAGCAGCCATTTCATT
CCGACAGATCGAAGTGAACATCACCCATTTTCGTTAAGGGGTTTCTTTCCCCTGCTCACCTGTTTGTTTTGGT
CATTATCGATTGGGTTGGAAAGGCGTGAGACAAGATAATCTCTTAAATGAGATCTTTTTACATCGGAATCCCC
ATCTCCTCTCTCGCATATGTTCACCAAGGAAGCCGGTTATAATATCTAACAATTCTCCAAGATTTCTTGTGGCG
TTCTGTTTCGTACTTCCACGTCCCAACATCATTTTGAAAGAAAAAAAACGATAAAAAAACAAGCAATGAATACA
CATCCCATCTTTCCATGTTTCAAAAAAAAACGATAAAAAAACAAGCAATGAATACACATCCCATCTTTCCATGTT
TCAAAAAAAAACGATAAAAAAACAAGCAATGAATACACATCCCATCTTTCCATGTTTCA

>20 c22501_g1
TTCTTTTTTTTTCCATTTATCAAATAGCAATACAAAACGCGTATCGGTTCTAGATGTTCACTAAACCCTTTGTTT
AAAATATATAAATATGCAAATAGAAAAAAAGCTAATAAAAAGCATTGAGAGGAGATTAGGAGGACATCATAA
TTTCATGTACAATTTCGTAAAACAAACAGAACATTTTTTAGTAAAAATTAATTTTGGTTTTTTGTTTTATTTTTT
GAAGACAGGGTATATTTTTCAGAGCGCCGCAACGGCACACGTGAGCGAATTCATCGATTCAGAAGATTTCG
TCCCGCTGTTTATTCGTTCAGCCACATCGACGCCGCCGCCAATGGGATGGCCTTCGTCACTCTCCTCGCCGCT
GTCCGGAAAGGAAGCCGAAGAGGGTGGAGGATCCAGTGCCGAATGGAGCGTGACTTGAACGTTGCGGTT
GAGCGAAACGGCCGGATAGAAAACGCCGCATAGGTCGACGAATGCCACCGTTCCCTGAGGCTCTTCGTTGA
CGAAGAAACTCAATTGACGCCGATCGAGGTCGAGTAGGACGCCAACTGTAGAACCGACGTCGATTCCGCCA
TCGGTTCGATGACTGTGCACGTTATTGTGGAGGAACCAAGACCGTTGATGATCAATATACATGGACCATCCTA
ATTCATCTTTACCCAGCATGGAGTCTCTTGCGACGTCTCGCCTTGCCACGCCAAATGCTGGATCGGCTGCTCC
ATCGTAACGGTCGACGGTGTATTCCCAGTAGTGGACGCCACGTGAGAAACCGACTGAACTGAGCAGAACAC
GCGGTTCGTAACTGTCGGTCGTGGCAGTTAGTCCGTCTGCTGATAAACGAGTTTCTGCTGGATAGGCTGTCA

TGTCAAATGTGAACCAAGCCACTTCGGCGGTGTGTAGACCCAGCGAATCGCTGTAGGGCCCTTCACCGCTG
CTGTTGAAGGCTTTAACACGGGCATTGTAAAGCGAATTGAAATGCAGGCCGTCCACCGTGCAGATGGTTTC
TTTGCCACAGTAAACCTCGCGGAAACGGCCGGCGGCGACGCCTCCTCCATCGTCCAGCTCGAGGACGTAAC
CTTCGACAAAGGAGGCCGGATGCGGTTGCCAAGCTACGGTGATTGAATTGTTTTCTGCGCTGCAATCCTCG
GCCAAGATTCTTGGTGGCCCGGGAGGTTTCATTTCCAGGAAAGTCAGTTGATCAATGTTACGCCGCAGACC
TAAATCGTCTAACGTGAGCTCAAGTGTCGGACTAGCCCGATTGGTCAATTCGTCCATTTCCTGCCGCCAAGTC
AGATCTAAATTGGAGACGCGATTGACTAGCATCGACCCAATCTGAAGAAAAGCCGATGAATCGGATTCTTTC
AAAGCTTCGATGCAGAATTGAACGAGCGCCGTCGTGTGCTGCAGGTGAGCCGTGCAAGTGGCCACCTCTTC
CCGCAATCGTCGCAATTGCAAGTCACGTTCACGCCGGGCAAAGTCCACCAGGGCCTCTTCCCTGCGTCGCA
GTGCTTCCATCAGTTCCAGACATTGAGCATGGATGGCCGATTCGACGTTGGCACAACTCTCCTGGACCCGTT
CCGACGAGCTCTTCAAGCGCTGGATCACCTCCGTCGCACTTTTGGCTTTCTCCGACAGCTGTTGCAACGATT
GCGTCAACTCGGCCTTTTGCTGTTTGCAGATAAGGGCCAATAGATGGACGTCGTGATTTTGATGTCGGCCAG
CATCCGATAAGACGCACAAGTGGCAAGCTCCCAATCGACAAGTGTTGCAATAATATGATTGCACCTGATCACC
GTGTTCGCTGCAGTGTGGAACGGCGTCTGCAGACGACTGCTGCTGCTGTGTTGGAAAGCCAAAAACAAAA
AGAAGGTGGGGTGACGATGATGTGGCACGCTATTTTGCTTCATTGGATGACGGCTGTGAGTGGAGATAAGA
GCGTGTGCTGCCGCTGCAAGTTGGAAACCAATTATAAAGAAAAAGTTTAAGACGACGAAAGAAGGTGTATA
CACCAGGTTTCCCAGACAACGACGATCCCATATCGTCACGACCATTATAACCATAGCGTAACCATAAAAGGAT
AACGTGCCAACTGTAATAGATCTATAACAAGAAGCGACTATTCAAAACGCGGGAATGGGTACAATTTTCTGG
AAGATCTATCGTTAAAGGAGAAAGTCTTGATCAGCTTTATCGGCGATCATTTTAGCGATCGCGAGAGAACTA
GTGGCTCCAGGTGAAGGGGTGTTGCGGCAGTGAATAATGTTACGACCGATTTCACCCATTCCGTAGTCGAA
AACAAAGTCCTCTATTAGACTGCCGTCAGCACCGAGAGCTTGAGCACGGACCCCAGCTGGTCCTCTGCAAA
ATGGAAATAAAAGTGATCAACAGTGATGCTGGAAATGGACGTACATTAAAGAGCTCTTTCACTGCTAAACTG
ACGAAGGCGGACCGAACGATTTCTTGAACTCCGAACGAAGCATATTTCAATGCTAATTTCTGGAAACCACGA
TAACTCAACGAATCGGCAAGGTCACGTAAGTTAATGTCTCCCCAGCTGTATCCTTCACGCTTCAAAGCTAACA
CAGCATTAGGCCCCAGCCAAACAGATCCATCTATTCTTGGGGTAAAGTGGACACCGAGGAATGGGAAACGA
GGATCAGGTACCGGGTAGATATTTCCTTTCACTAAGTGTGTTTTCGCAGGATTGAGCAAAAGATATTCGCCAC
GGAAAGGCACAATACGTGGCTCCCTCGAGCAGCCTGAAAGTTCTGCCAAACGATCCGATTGCAGGCCACCA
CACGTCAGGACGTACCGGCAGCGTACAGATTGCCCATTGCTTGCTGTGACTTGTATGCCTTTTTTATTTGCTG
CGACCGCCTTGACTTTACTTTCTTCGGCAACTTTGAAATCCTTAACTTCAAAGTTGAGATGAATTTGACCTCC
AAGTGATTTGAAATCCTCGCCGTAATACTGAGTAACTAAACCCCAGTCCACGATTCCGGTATGTGGCGACCAC
ACTGCTTTCAGACCCTGACAGTAGGGCTCTATTTCTTTGATTTTTGCTCCATCAACCATTTGAATATCTGGGAC
ACCGTTCTTAATTCCGCGATTGTAGAGATCCTCCAGCCTTGGCAACTCCTCTTTAGAAACAGCTACAATAAGC
TTGCCACATTTTTTGTAGAGAATCCCTTTCTCATCAAAATACTTGTAGGACAGGTGGAGGCCTTCAACACACA
GTTTTGCTTTCAGCGAGCCAGGTTTATAGTAAATCCCACCATGTATCACTCCGCTATTGTGCCCACTTTGATGT
GCAGCTAAACGGTTTTCTTTTTCAAGAACTGCAAATTTTAAATTTGGATGTCTCATTAACATCTCCCTGGCAG
CTGCCATCCCAACAATTCCGCCTCCAACAATAACAACATCATATTCTGGCTCCGTTCGTTGAGATGAAAATTTT
CTATTTATAGCCGATGACGAAGCTGGAACACAAACTGCTCGTTGTACATTTTGCCTGTGTCTCGAAAACGAA
GAACACTGGCGTGTATTTCGAAAAACGTTGTGCATTGTGCTAATCTTCGACAACTTGTTCGTTCGTCTTGCGT
GTTACGTGAGATGTTGGTTTATGTAAGTTTATGTAATGTTTGTTGTCGTGCCGAGGGTCAGGTCGACGAGTAC
GAGAGCCGGCAAGGGCAACAACAAGGTTGAACGTTCAGCTTAAACAGTTCTTTCACACTCCAAAAGGTGG
CCTCAATTTATTCCCAAAAAGAAATCACACTGCCATCACGTGCTGAGCAGTTCTTTGAATCTGTAATCTCGTCC
TTGAGTTGATTTCTGATTGTTTTCCAGCTAGTAATCAAATACGACTAGTTCGTACAATCTTCGCGATAACGTGG
CTTAATAAGCATTATACAGCATTTTACAGCTGTTAAGGAAAACCTTGTATTTTTCGATTGGCTCCAAACTCCGG
TAAGACACTCTTCCATCTGTTTCAGTTTGTGAATTTCTGATGCACATGGGCATGCACATGGGTAAGCAACGAT

AAACGAACGGAATAGTTTAAACAAAAACGTCTTGGCTAGCTTGTAGGAAAATTCTAATGTTATTGGATTAGT
GGCCTTGATGTTGAAATTTCAGACAAAACTTCGCTATTTACGAAATTCTGTGTGGGAGCATCTGTCGATGGG
TGTTCTGTTGAAGGCTGTTTCTGATCAGCGGTGGACTCGAGCAGCAGCTTGACAAGCACCTTGGCGTCTTC
GTTGGTAAAATCAATTTTTTTGTGTCCTGTTTTACCAATGTAGAGATGCTTAAATCGGCCTTTGAGACGGCGA
AGAAGGTATCCGGTGTTCCAAGCATTTGTAGTTTCTTGACTGAGGTAACCTACAAGGCGGGCAATTACTTGT
TCAGAGACGACTAATTCATCATCTTTTCCCAGTTGGAGGCTGACAACTGCTTCACTTGGTTGAATTTTCTTGG
GCTTGAACCTTGCTGACATCAACTGTCCATCCAAAACCTTTAGGCGGAGTCGAGCCATCAATGCAGGAAATG
CAACTCGTGATTTCTTAAACGGAAAGGCAGCTTTTTTCTCCATGACGAATGTCAATGGGCCAATTTGCAGCT
GAACATCGACGACGTGAACAGATGAATTCTCTCGTGAACCGATCATGACATCGTCACTTCTTTCAATCAATTC
TACACCTTCCACTCGAGTAAATGTTTTTCGTGAGTAACGCCGGGCGTGAGCTCGGCGTTGCTTTCTGCTCATC
TTCAGGTCTTTACCCTCAACTTTCAATCCTGTAGATGGCTCTACTGACGATCCATTTGTACAGTGGATTTCCTG
TTGGCTTAGCAACTTCACATAATATTCGGTGGCACCGTCGGATAGCACAACAGGTGATGGCTTTGAAATTAG
ATGAAAGCGTTTCGAATTGTTGTTCTCTACGCAACTAATATTGGCAGGGGTTTCGCTAACTGCATCTTCCTCG
TCGTCATCGTCATCATCATCATCTTCTGCAGCTTTATTGGCTCCTTCTTTCCCACTGCTGACCTCAGCTGATGTT
TGTAGTTCATCCTCCGTATCGGCAGATAGTTTAGACTCGCCTTCGGCGTTTGCGTCATCTTCGTTATCGTCGAC
ATCACTATCGGTTTCCACTTCGGATGTTTCCAGTTTATTGGCGTTATCCGATTCACCAGTGATACTTTTTTCGCC
CGAACCAGCGCTACCGGCGAGTTGAACGAACATCCATGGAACAATTTTCATATCTTCTTGAGGTTTATTTAAT
CTTAAATGAAATGTGGAGTTTCCATTGTTCAATGTTAGCGAAAAAAGTGCGAGCATCTTATCCATGGCATCGT
TCAACTGGTCAGTATGGGACGATCGTTCCTTGTACTGGATTTTCGAGTAGAATTTCTTGTTGCCGTTCGCCAG
ACGATTTTTGCGTTCCTCAATTCGCTTTGCTTTTTTACTCCAACGATGGTGGTGGTGATGATGGCGGTGGTGG
TGATCGATAAAACGATTCTTTTCTCGTCTGTGTAACCATTTTTTCTTCCATTTTTGCCTTCTTTCCAAATTCGAT
GTTGCCGCGTCCGGGTTGATGCCACTAATTTCAGAGGGGTCGGTTTCTTCCTCTTCTTCGCTTTCATCGGTCG
TTACGCATATGATGCATACCTTTGCTCCCTTGACCGAGACATCCGTCGAACTGTCTTCGGCTCCAGTACCTTCA
ATTGAATTGGATAAAACAGGCGAAACACATAAAATAACAAGGCCCAAAAAGAGTAGCCTCATGTTGGAATCT
TACAACAAGCGAGGTTTGATGACTGTAATTCTAGCTTAACGTGTACGTCTTCACCAACGATTGACGATAAATT
TTCTGTGCAGAATTGTAGCCGGCTTCCATATATATACCACTAGGAATCTAGTAGGACAAATCGTTAGTTACGAA
TCGCTGATACCATGAACTGCGTAGTACACCAGCAGACATGTCAAAGAAGCCGACTGGGTTTTTCCATCATGT
TAACGCCGTGGCTGCAGTTCTGTTAAATTTCGGTCTAAATTACAGATGTCAACCACACATGCATCTAATGCCTT
CAGGCCATTCAAAGATGTTACTCTTTCCTACATTGGTGGCTTTTTGTATCCTGATATTTTCATGGTGGCCTCAG
CCATTAAATGCAAACCTCAGTAGATCACTGCTGCAACTTGATGATCTACCCAGCAGTGACCTGAACCTGATGG
AGTTGCTTTGCTTGAAAATCTAGCAGAACAGGAGAACGTTTTCATTCCCTACCGCAAAGAACTAGTAAGAAA
AACAAACTTTGGCACGCCCAATTAAATTTTTCCTGCATCGTCAAGGGTTTTTACTCGCTTCGTTTGACAAACG
ACGAAACGAATCAACACAGATTTACTGTCGGCGTGAAAACAATGCAGTTTGAAAATCCCGCTATTTGTGTCG
TCAAGGTACCTAGCCACCTAGCGGTCGTTATAAACATTACGAAAAGGGGATTAGAAGATGATAACAAACATT
CCCAATGGAGAAAACACAGTCCTCTTTGGCTTTGGTTTCAGTTGAATTTGAAATATTTGGAAGGGTCCAAGG
TGTTTTCTTCCGCAAATATACTCGTGATCAAGGTAACAAATTGGGACTGAAAGGATGGTGTCGAAACACCGA
AGCCGGTACGGTTGAAGGAGTCATGGAGGGAACCCAAGAGCACGTGAACATGATGAAGGAATGGTTAAG
ATACAAAGGGAGTCCACAGTCACGCATCGATAAAGCGGAATTCTGCAACGAAAAATTCATTGAAAGTCCAA
CATTTACCACATTTGGTGTAAAAAAATAATGCTATTGAACAACAAAAAATCATGTAAAATACAGATTTAAAATA
AAAAACATGTCAAGAGTGAAAGTGA

>21 c23556_g2
CTGCGAGATAAAAAAAGAAAATCAGTACAAGCAGACGACGGTGAGAAATATTCTTGGACAGAACAACAATA
AAATGAATTATTCTTAAAAACAAAGAAACGAACAAAAAGGGCAAAATAAATCTTCTCAGCTAGATGACTTCA

CATGATTGAAAAACCGGAATACCAGTATTTTTTTTTTGCCAACCAGTCTGGTTTACACTCGCAAAATGTATGC
ACGCAGGGTGGCTCATCCCGTCGCCATATGCAAAACACGGTCGTGCTGGCATATAAAAGAGCAGGAAGAAC
TGCAGAAAAACATCGCCTCCTCAGTCAGTTCAGACTACTGTTCGATTCAACTTCTCTTCAGTTGCTTCTCCTC
AAGTATACTCTTTTATTACTCACCATGTCTAATTTGTTCTTGGTATCTTTCTTGATTGGCTGCTGTGTATCGTCTG
CTTGGGCCAGTCTCAATCTTCATGACGGACCTCTCCTTTCGGGAGTGACCTCTTACACCATCACGGTAGAGA
CAAAGACAGTGGACAAAAGACATACTTGTTACGTAACAGAAGGCGTAGTCAACCAATGTCGGCGAAAACGT
GGCATGGAAGAGCAACCCGTCTACGAAGGTTTGGAGATTCAACCATCGGAAGTAATTGGAATTGAAGCTAC
TCCCGTTCCTCGTGCATTGAAACCGATGAATTTCCACGATTCCGAAAAGGTTATCGGCTCATTCGACGATGCC
TATGTCAACTCTCAGAATATTTTCCGACAAATTGCTGTTAAGGGCCGTAGCAATAAAATCACGGTCGGTGATT
GCGGCAAATCCACCGTCAATCTCAGCGAGTTCCTTAACTGTCTGGGCATGACTGTTCAAGAAACTACAACGT
TGACGGCCACTTTTACAGAGCTCACAACCAACTACGTTGGTTACGCCACAATGACCGTCAAGCATTGTACCC
CAGCAGGTTTCCCTTACACTTATTGCCCTGTGTGAATTCAGCATCCTGCATATTGAATTTCGTTAATGGTAAAA
GACTTGTGCAATTCCTGCCCGATCCCTGGAAATGGAAGAGATTCCTTCAAGCCCTGGAAGATCACTTATGAA
AGAGTAATCTTTCAGGTGGATCATCTGTTGAGGTGGTCTTGATGTTGACTTTTCCATTGTTTTCGTTATTTGTT
TTCTAAATTATTTGATACTTTCTTTCCACGGCTTTTAAACAACCAACTTCAATTTTCGGCTATCTAAAATCGTTT
TCTTCTTCTTCTTCTAAATTATACCGACTTTTGCCACATAAT

>22 c16151_g1
AGGACGCGCCTGGCTGCCAAGGTAAGTCGAATTATTTTGTCACTCTTCTCTGTAATTCAAGTAAAATTAGTTG
GGATTTTTTGCAAAACCACCACAATTAATACTAACTAGAGTGACGTTATGAGGATTTTTGCACAATTTTTCTTG
TGGTTTAGAACGTAGGCAAAACGAGTTAACGAATTTTTCTCCAAAGTCTCATCTCCAAACCCTTGGGTTGGC
TTTGCTTTCCCTTTTGGTCGGGCACGCGGGGGCCCGCCATAGAACAAGTCTACCCGTTTTTCCTCCGTTTGA
CGCAGTGTATCTCTGTGTGTGTGCGCCAGTGTGTGTTAGTGTCCCTGTGTGTGTGTACCCCAATAACGGCGTG
TAGATATTCCCGGCCCTACGCGTTCTGAGAAAAAAAAAAATTATCCATTTTGGTATTTACGCCATTTAGGTTTTT
AGTCTTAAAGAGTAAAGGGATCCAACTGTTTGATTAAAGATGGCTGATGCTGCATCACCTAAGAAACGCGGA
CGTCCTGCAAAGAAAGCTGCTGATGGCGAACACAAAGAGAAAGATGAGCCCAAGAAGGCCGAAAAGAGG
GTGGCTGCTCCAGCACCAGCAGCATCATCAGGAGGCGAAAGCGGCGAATCTCAGGTGAAGCGAGGCCGA
GGCCGTCCTAAAGGATCTGGCAAGAAAGCCAGTGGAGCCGCAGGAGCGTCTGCTGCCAAGTCCAAGCCCC
CTGCCGGCGGTCGTGGCCGTGGCCGTCCCAAGAAGAGCGAAGCCAGCAAAAAGGATGAATCAGCCGAAG
ATGAGTCCGCCGGAGATGAAGGAGAGTCGTCATAAAACGTGTTCACAAACTACCCACAACTAAATTGGGAG
TTTTTTTTAATTTTCATCGTTTTTTGAGTTCGACATAGAACACGATTCGACGAGATTATTAGAACACGCCTCTT
AAAAAACAAAATGCAGAAAACCATGTCGCGTCATCCATTATTTTAGTTTTTTTTTTCTTTTAACACAACTTATC
CACATCTCATTACACCCCACCATCTTTTAAATAGTGTCCACCCCTCTTGTAAACCCTTAACAGTAAATCGCCAA
CATCTGCTCCTGTTTTTTTTTGTTTTTAAGTCCTCCATCCTTTCTCACCGTCGTCTCGTGTGCGTACGTCTCCCT
TTTTACTCTTATTATACCGTTTTCATCCCGACCTGATGGTGTGTAAGTGCAGTTCTTAGTCTCTCCTATTTTTACT
TGGATGAATTCTTGGATGGATGATACCCTCCTCCGTTTCTCGCACCTGTAAAATCCCCATCTCGGCAAAGAAA
CACAACTCTGCCATCCCATCCGATTCTGCACATCATACATTGAATTCAACAACTGAGCCACAAACATATTATTA
AACAACGTTTCACTCGTGACGCTATCCTCATTTTCCTGGCCATCCAATACAAGATTTCGGTTGATAAGAAAAC
CATTTCAACTTGACTTGTTTAGTAATCTCGTATTATTGATCGCACACAGAACGAGAGGAAAACCCAACCGTCA
TAACAAACTTCCAATCTCAATCTTCCAAGGCGTTGACTAGAAGGCGTGGCAGGAATCAGGATGAAAGATGA
TTAGGATAAACACACAGGAGAACAAAAAACAGGGAAATGGGATCACACAAGGATAAATGTTATTCTTCATTC
AACGTCCACCTTCAGCCGAGAAACTGCAATCGCGTACAGCTCGTCAAACGGCGAATCCATAAATAACTCGTG
AAGAAAACGGCGCGCGCGCAATCGGTAGCCGTGAGATGCCATGACGTGACAGACATCTGAGAAGAGACAG
ACATCGTGAAACGCCCATGGGAATCGCTGCTTTAACCTGAGAAGACCTTGCTCATTTGCATTCAACCCAACG

GAGCTTCCCATATTTGTTACAAGCCGTAATAATTCTTTGCGAACTAGCCATTTTTCCTCCTCGGTTTGTTTTGT
GTCTTGTTCCACGATCGCGGATGGATCTTTGGAGCAAACGATGCAGTCCTGCCATTGGTGAACAAAATGGG
GCTCTTCATTGAGTGTCATGTTGGAAAAACTGGTGCAAGGCATCGGAGGAACTGGTTGCTCAAACCAATCC
GCTGGTGGATGGAGGTATGATAGCTGAATCGGCAAACAGAGTCCAATGTAACAACCAGACCTATTGTTCAAC
GAGCTCAAAAGACGCGGCCTTCTTTTATACGACAATGATCTTAAACGACTCAGAATGGAGCCGATGCGATTG
AGTTGATACTCAAAATGTAATGGTGCTGTATCCCTTTCTTCTCTATCGTCCTCTTCGCTGGACGCTTCTTCGGC
AGCAGAAGAGGAGAAGGTTTCTTCTGTAGAACACATCGCTCCATCCGTCGGTTCAGAGCGGAGCAACTGG
ATAGGTTCAGTTTCTGGTGGTATAAAGGAACTTTGTTTCGACGACGATCGCACAGGCAAAGAAAAGCGTTT
AGATCTACGTAAACGTAAACTGGTACGTAATTTACTCCAAATGCGGGACTCTCTTTTCGGTCTCGTTGTTGGA
GAAGTAGGAACCGCACACGACACGCCATCTGCCATTGTGTAATGCGAATCGCTGAAGCTGCGGAAGTGAGG
CACGTATTCTTGTCCAGGGGTTTGAACCGAAAGAGGCAACGTGTACGCTCTTCGGCAACTCCGTATGGCATC
TTCCGGCGTGGGCACATCTGACGAATGGTCAGAATCCGAATCAAAATTGATGGACGAGCCTTCAAGTGAGT
ATGGCTGTGAATCCGTTTCCCAGCAACCATTCGGTTGTTCAACTGAGCCACTTCTTTGTCTAGGAGTTGAGG
TTAATATCGATGCTGCTCTTGTATCATCTCTTTCGTCAGCTGCAAAGGTAACACTGTGACAGGAACTCTGGCTC
ACGGTGCGAGGACGGTCGCGAACGACACCGAGATGAGAATTGTCTTTCAGTATCAGTGGGTCACTGACATG
TTGGAAATATAAGGAATTCCACTCGGAGTCGTCCAAGACATGCCATAGTTCACCATGATGTCGTTTTATGGAA
GCCCAACCAAATTGGGCAAGAATGTCGACTCCAGCGCTTGTCATGGCGATTAGATTTAGGGCGAAAAAAGC
TGTGCCTCTTAGAGTTAATACAGATGAGAATGACGCAATACGAACCAAGGCTTCCACCACTTGAGCTGATTC
CAATAATGCAACACCGGGTTCCGAAGTTCCCAGATGAGCACATGCCCATACAGCAGCTTTGAGTTCTAATATC
TCTTCATCAGTTTCAATGGAGGCGTTTGTAACCACTGAAAAAAGCTTGGATAGCTGTCCTGAGGTACGCAAA
AAATCTAGCCCTTCTCTATGCTGATTTAACTGGGAGAAAAGATGTGGCGGCATAGGCAAGTCTCGAATTGCA
TGGCGTACGTTGCTCAGACGGCCATATCGACCATCTTCCGCTCGCTGATGCTGAGTCAGACCATCGGCTAAG
TCACTTTCAACTAACTGGACATATCGAATATTTAAATTGCTAATCCACTGATCCAACTCTTTCTGAACAAGGGA
CACTTTTCCATTCTCGGAATCGGTGACGGTTTCAAGCCAATGTTTGAATCCAGCTGGGATCGACAGAGTTTT
GATAAGTAATAGTCGAGCACGAGAATCTAAATGCTGCTTTTCCTGCTCCTGAATTAGCTGAACGAACTGCGTC
TGCAGCGGGATCAATACCTCAATGCATGCCGGATCGTCTGTTGCTTCATCTAAAATATCTGCAGCAGTTATGAC
AACAGCCCGGCTTTTATCATTCAATTGACCAAGTAGCAACGGAACAGCCCACTTTGCAAGAACGCTGGTCGT
TCCATATTCTTCTGCCGCTCGCAGTAGAACGCGAATATGATTTGTCGCATAAATTCGACCAGAATCGACAGGT
CCAAGTAAGATCTTTTTCAAGATAGCTCTGGCATATCCGTCGGTGTGAAAATCAAGTGTGGAAACGATGACT
TTCAGATAACAATCGTGGTTCGTGCTCGCCACCAATTCTAAAAGATCTTGAAAAATGCCGGCCTTTTCCAAGG
CATTAATTCCATTGGTGGTACTGCTCAGCCGGCCAATAAATAAGAAGTAATCCTGGCAAGCTGTTGTGGAAA
GTCGACCAGGGCTAAGCAAGCAATCGTGGGCTGAAGAAGCTTGTGATATATCTCGGATTGCGCTACTGACAT
CGTCAAGGAAATCGGACAAGATTTTGTCGCACTCAAACTCTTCGACAACTGTAAGGGCGTCCATCAAGTGA
CAACCAACAGAAGAATAGAGACGGGTCCGGGTGTTACTGAACTCGCACTGGCAAAACTGGTTGCTGCTGG
GCTTGTAAAACTGCATCATTCGGCGAATAATCGAGCGCAAGATGGAATCTTCCAACGCACAAGAATTCATCTT
GCTCCACTGGAGAAGATTGGACAAGACAGGCCAGTTCCAGATCAATGGATCTTTCTGTGCTAAAACACCAC
AGTCTCGTGAAAGCGCAGAAAGAATGCCATCGGGATCTCTACTGGAACGCATTTCCGACTCAACTTCTCCAT
TATTCTGGAACTCCTGGTCGTCTCGTATGACGGTAGCTGCGTTCTCCTCAGTTTTCTTATTTTTGGCTGAAAAA
CCTTTTGCATTGTTGGAAGCTCTTCGTTCAAGTTCAACGATCTGCTTGAGAGCCAAACTATACGGAACGATTC
CTTTTTTCTTTAGTTGATGGATTTTTGAAAGGGCCGCTGCTGCTTGAGCGGCTCGATGCCGTTTTACAGGATC
GTTTTTGAGGGAGGTAATGCTGTCGACTAATTGAGGAAGGCACATCTTTGTACCGTGGCACGATGGTGGTA
ATAGCGTACTACCAAGATGCAACAGTTCCCCAAGTAAGATCGTAGCACGAACTGAGATGAACGTGTCACTAG
TTACAATGACTTCCACCAGTGTCTGGACAAGTCGCGCTTCAACCAAGCAATACACTAAGAGGGCCGTATGAT
TCAATACTAAGTTTGGTCTGAACTTTGAAATACGTGGTAGCAAGCATTTGCCCTCTGCGGCTACATAACCCTC

TTGAAGTTTCCAAGCATCTCGTACGCGGGAGGGGTCAGTGGCTAGAAGCGCCACAGAAAATTCGTCTGTCC
ATTCCGGAAGTGGTAATCGCAGCAACGTATAGAAGAATTCTAACATTGATTTCCTGGTTTCTAAATGACAGGG
TAGTAAGACCCGGACCAAGTCTTCTAGGATTTTACAGCTCATGACATGTAATACTCCATTCCAAGATCTGAGC
ACCGTGAGAAGAGCTTGCTTGGCACAAAGCAGGCGGAGCTCACGGTCATCTGTCGTGTCGGCGGAAGTAT
TGTTCCCTTCCCCTGACTCTGCATGGTGCTTGTAGTGAAAATCACTGTAGGGAGCAATCAGAAATCGCAGGT
TCAATTTTGAAAGCTCTCGCCGCTGCGCCGTGTCAAACAAGTATAGCAGAGAAGCAACCATAGCTTCCTGCA
TACGAGCAGAAGAGCAGTCCAAGACCGCAGAAGAGAAACATCGAACACCTCCGCTGGCAACAAACACAG
ACGGGTTAACGATACAAAGTTCTGCAAGAATAGCAAGTGATGCTCTAACCAAGCGGTCTTTTTCTTTGGTGC
CATCAAAAGCCAAGGCCACAATTGAACGGGCCAGAGCAATAGGAAATAAAGATGGGTCTACCAGTAACAAT
CTTCTGCATAATCTCAAGGCCTGGGCACGTTCTGCCAAGTTATCCACAAGCACATCCAAGCACTTTGCAATGA
AAAACTGCAGATCTGCTTCAACCAATTTTTTTGCATACTCTTCATTTTTTAGGCATAATCTGATCACTCTTAAGC
CAGCAGCTCTTACTGCTTTGCTGTCGTTAATCAATGGAATCCGCAAACATATGAAGAAATCCAAGCAGTCAA
GTTCAATACTGTCACAGCCTCTTTTCTCAATATGCTTTGATATCACATTTAACACCGATCCTACATGAGGTTTGC
CAAGATTGAAGGAATCCTTACAACACATCTGAAGCAAAGCATCTCTAATGTTGGGAGTTGCATCTGTTTGAG
CACCATTCGCACATGTCGAAGAAACTAGCACGTCTGATGTAGCATTGATTTTGACAGTTCGCCCCATTCTTCG
AGGCGCCTCCATTTTCGAAGGTTGACAAACAAGTGAACGAATACGCGCGCGATTTTAGTTTTTTGAACAAG
AAACGTGTTTCTCAATAGCAAATGAGTGGGACTCGAGCTGTCACTGCCATTTAATATTTGCGTTCAGAGCTG
GGTTGTAATCATAGTTGCAATTC

>23 c16413_g2
CGGGGACACTTGAACATCAAGTCAAACTCAGCAGCTCTCCTCTTCATTCAACATGTACAAGTCAATGAGCCT
TTTGGTTTTGGTGGCTTTGGCTGCTTGCGTTGTCTCCATGGAGACCCAGGACCAGGAAGCTGCTGAGCAAT
ACTACCGCGTGCACGGCTTTTACCCCACCTGGTACACCCCAGTCGCCACAGCCGTCACTGGAGTCCGCACTT
ACCCCTACCCTACCTATGGCAGCTTCCCATTCACTTATGCCGGTTATAATACCTACCCCGCCGCTTTCACTGGT
GTCAAGAATGTTGCCACACCTTACACCGCCGCTGCCTACCCCTACCCCTACGGATATGCATACAACACCTACC
CGTACGCTGCTTACGGCGCTGTTCCTTATGTTGCTGCCGCCCCTGCTGCCAACCCCGTCAAGGCCTAGATTAT
TTGTTACGTAACACCGACACGATCTTCAACGACCAGAGCTGGACTCGAATTAAATTTGCCGCACTTTACATCA
TCTGTGGCTGATCAAACCAATTTTTTTTTATAATGTTGCATTTTGGTACACAATGTTGATGTTCTGATGTGAAG
AAAAACAATCATCATTTGGTTAAAATTAAAATAATAAAATATGAAAATCAAAAAAAAAAAAA

>24 c12941_g1
ATATTTCTACTCTTATCCTTTCTACCCCGCTGCTGCCCCCGCTGCTGCGGCTCCCGTTGCCGCTGAGAGCTCCC
GCAAGAAACGTCAGGTCTTGCCTTACAGCACTGGTTTCTACCCCGCTACTCTCCCGTACGTTGCACCCGTTCC
AGTGGTAGCCAAGACTACATTGAAAACTGTCACTGCCGAGGCTGACTTGACTGCCAAGACTCCGGCTAGTA
CCAACAAACTCGATTTGAAGGAGAAGAGCGTCGATGTTGTCACTCCTGTGGCGTATCCCACTCCTTACGCTT
ACACCTACCCTCATGCCTACCCTTTCGTCAACGGATTCTATGGTTTTTATTAAATGTCTAGATGGAATTGATGTG
CTGGACAACGTCAACAAGATCTTCCCCTGATTCCTGTGAAAATTAGATGTGTTTTGTCGATGGAATTCATTTT
GCAAGCACGAAAACAAATTATTGCATTGATATTCAAATAAAATTGGTATTTCAAACAAAAATAACAAAGGAAT
GAATTCATTCCACACAGCTTGTTTCAACTGAAAAACTTAAAATACAGCTGCGAATTGGGTAGAAACCAAACC
TTAAATACAAAGTATCGACAAAGCTCATTATTCTTAATCATAAACCTGTTTTTGCCGTACACACTATTGTGTTCG
TCTTCAAAATACATATGACGAGTTTTCAATAATTATAAAAAGCATAACGACAGCATTTAA

>25 c19155_g1
TTTTTACAATACAAACTCACTGTATTTTGCCGCCCACGAATCGAGACACGCAGTTTTCGTATAAAAAAATAATCA

AAGGAAAGAAAACACACACCAGAAAAAAAGGGAGTCTTAAAAAATCACACGCAGAGAAGATTTTTTTTC
TCCGTGAACTAATAGTAGAAAAAAAAACACAATCGTGTAAACAAAATATGCATTTCCGATTCTGGACCTTCAT
GGAAAGTTTTTTTGACAAGCTTGCAGAGAAGTGGAGAATAATCAAACAGAAACGGATCAAACAAAAGGAG
AGAAAAAAAAATTAACTGTCACCCTAAAAACGTGGTGGAGAGAATATTGCCAAGAAAATTAGAAGATAGAA
AAACGTATGCAAATCTAGACTTGGAAATCCGGAAGATGTAGAAAAAAAAGACTATTTCGGGGACTACACAC
CTGTGAGATGAAACTCATACACTACCAAAAAAAACCTAATAAACTCATATGGAAAAGAGCGGAAAAAATTTT
TTTTTCAAGAAAAACCGTCGGTGTACTTTTTCAGTCCCTGCTTAAAGTATATTCGTCAACAAACAATTCATTCC
CCACGCAAAAAAAAAATATCTACTAAAAGGCATATAAGTAGAATACCAGAAGGCGCAAATACACGTACGAGG
GGAGAACGGTGAGCCCCGCCCGATATCAATTGCAAAAAGATGTTATTCAATTACCGGCATGGAAGACTGATT
GATTTCATTGGTCCCAAAACTCGTATTTCTTAGTTTTGTCTTACTTCTATTTTGAGCGCTCTCTAATTGGGTGTT
TTTTTGTGTTAGATGAGTGGGCGAAAATGTAGGGAAAATAATCTAACTTTGCTGGGAGAAAGAGATGAGGG
GGAATTAAAAAAAAAGATCGCTAGATAACATTGGCTGAAGACAACTCTTGTGAGTTCTGATCAAGCTGACG
GGGTCCCACCAATGGCGTCTTGAATATGCTGGTTAAACTGGTTCCTTTCCTTCGAATCGGCGTGCCAAATCGC
AGATCAATTCTTTATAGACGAGAGGGTCAATATTCTTTCCGAGAAGAAGGAGAACAAACCAATCTCCAATCT
GGCATTTGCGAGCCACCAATTCAACTTCATTGCGAGGTGCCAAGCGGGCCTGTGCTCTCAACAGGTACATG
CGGGCTTGAGAACCCAAGACAACAGCCAGACGGTACAGCAGCGCCAAACCGCTCACAACCGCCAACAAG
ATGAACCAGAACCAGAGGAACACGTAGATCTTTTCGTTGACAATGTTCAGAGGAAGAACGCACAGACCGTC
GATACGCGTTGTCGTACCGGAAGGACCGAATTTGTGGAACGTGCATTTGGTTACTTTAGGGAAAACCTTGG
CCATGGGATCAACACGGTCTTCGGGTTCCATTTCAGTCATGCTGATAACATCTCTGCCGTAGGTGGTGAATTC
TCCACCGAGGAAGAAATCGACGAAATAAATCTGACCGATGACGTTGATAAAGTTGAGTACTTCGCAGATAAA
GAATCGGATAGCGTAGAAATTGTGGTTGTGCAAGTTAGAGGCAAAGTATTCGACCAATAGTTTTTTTCGTTC
ATTTTTCGTCTCAACAGCGATGATGGGGCAGTTGAGGTCTAGGACGAGCATCTTCATCTTTCCGGCTTCCCAT
GTCTTCCACAGGTAGCGGGGAATGTAAAACAAAAGGGCCTGGAAGAAGAGGACGAAGCAGACCCACTGG
TAGTATTTATGGTATTTGACTTGATCGCCTTCCTGATGAGGGCGAACTCCAGGATGAGGAACATCTTTTCCAA
TCGTTTCGGATGCCAACCGATTGGGGATGGTGAATGTAGAATGGATCCAACAGTACGTGTCCATAACATTGC
CAGGTACGCCCTCCACGATGCAGTCGATGGGGTCGCCAATGTACTGGCGTGATGTTACCACCAAGGAAAAG
GCAATCAGCACAATCACCGTCGCTTTGTAATGCAGGCGGAAAATGTTGTTGTCAATATTGACAGAATCGAGC
TTTAGCAAGCCCTTCACCGATCCGAAGACGTCAAACATTTTCGACGACTTGGTGGTGTCTTTGTTTCAAAAA
AAAAATGTAACTAGGCCTTTGCAATCGGAAAAACAAAAGGTGGCGGAATGAGGTCTTCACGCCACAGCAAT
TCACTCGTACGCGTTCACCGCAGAGGAAGACGCGCCCAGAATTTCACTCGATTTAAACGACGTGTCTAATAT
ACGACGAGCAACGTGCCACGCGAACCGACACTGCCTACAGCTCAGCTCAGTCTGACAACGATGGCACAGTA
AAGCGATTATTGGCCGGTAGAGGCGCTTTTTACCCTGAGGCCGTCTGCAAGGGGACACGGAAATAAAGTCT
CGCCACGGGTAGAATGGAAAGTCACGTGATCTGGAAAAGTCTATCCCCTTCCTCTGGGACAGATGGCGT >2
6 c16110_g1
TAACAGTTCAAGTAGAACAACTATTTCTGGTGTTATTTTGAAATTTTCTTTTGATAGTTGTAACACATCGTTTTT
TAATTGATCGAAGTCCTTGTTCTTTTTCCCATGCTACAGCTCAGTGGTGAGTAAATCGATTGGTAAGCCCTGG
TGCGACTATAGCGCCGCCAGTTGTTGGCGTCTGCAACCTAAAAAATACCCGGCATTTCGAGATCGAACGTGT
ATACAGCGAGACCCTTCCATTCGTGCAGCGCCTTCCGTCAGACTAACAAGTGTGCAGACCTCTAGTAACACG
AGCTGACTAAGTTCTCCTACACACCGAAATTCGAACACTTTTTTAAATCTCTACTTTAACAGCAAGAAGTGTA
TTTGTAATCTAAAATGGCTGACACACACGTCGAAACCAGCAGCGACTTGAACCAAGATTCTTTGGACGGCG
CACAAGCTTCCGTCAGCGAAGTTGGATCAGCTGAAGAGTTTGAGAAGGAAAACACTGAAGACACCCCTTC
TGGATCTCATGGAGAAGCTGACGGTAACAAAAAAGAAGGTGAAGCAGTTGATGGAGCTAACGGAGAAGA
GAGCCGTGAGGCCGAAGATGCTGCCGTTCCCGCTGATGCCACCGCCGCGGCTGCTGTGACCGAAAACGGT
GCCGTTGAAAGTGAACCCCACGTAAATGGTAATGGAAAATGCGATGAGAATGCTGAGGCAGATTCCACGGA

AGTTGATGGATCCGCAGCTGAAAAGAGGAAATCAGTTGTCGAAACAGAGGCATTGAGCATTTCTCCCAAGA
AAGCTCGAGTTGAAGGCACTGATGAAGATGTAATTGGCACAGAGGAGGCTGCCCTTCCCACTAACGGAAA
CCACGAAACTGTCGCGTAAATGGAACAGAAACATCCGGCCCCCATTTTTTCTGAGTAACCTTCTCCCTTCCAA
ACCCACTTCCCCCGTTTTACCTTATTTTTATTTTGGTAAGCAGTTACTCTTTGCACACTTGTCTGTCTATGAATA
AGGATCAACGGCCATCACTTGTTGATTCAAATATGTATCTCATCATCTTCATTTGACGTGGATTTTTTTTTCAAC
AAAAAGCGCGTGCGTTCACTCATGTTGACTTAAACTGGAAGGGCAAATAAAGAATCAACTAAAGAAATTGT
TTCACAATGTATTTTGAATCAACTTTTGATGGTCTCCGTTTTACCTTTTTCACGAACGAATAAGAATCTATTATG
ATATTCGTTGTCAGTGTAGGATAGATTTTGGCATTGTCGATTGATACGAGCGTCATCTTAAAGTAATCGGTTAA
AATGTTCAACCCCATAGCGCAAGTTTCTCACATCGACATATCCATGTTACGTTATGTATCCAGCTCACATTAAAT
CGTCTGATGAACGACTTTCTAAACACGGACATTTCTGCACCTTCGTTCCGATGGAATGCGCAATTTGTCTTTG
ACATTTAGCGGTCCTTTTTTTGTATTTTTGAAGACAAACACTTATTTAAAGATTGGTCGTTAACGTAAGACTTA
CCATCTACCGATCAGTTTCCTATGTTTGATTCTATCAGCTTTTGGCGATGGCGATTCCATCTACTTTCATTAAAC
CTGGGTGTTGTGATTTCTTCCTTAGCTCTTTCCGACATAAAGTTGCATCCGTTCAAAAGTTCAAAAATTTGCG
CGCGCTCTACCCAAAAAGCTGCCCGCCACCTCTTCTATGTGTCCCTCGCGCACTTACTGTCGTGTCCATTTCCT
AACTATAATTCATTTTTTGGAAGTCGTCTCATCGTTTGCGTTAACCTTTCGTCAACACCGACATTATAGAGAAG
CTCGGTCAAAAAGTTAATCTTTTTCCTGCCAATACAGTTTTCCTCTTTCCATTTCCCCTTCTTTTTATTCACCATA
CAGTTCGGTATCTTCATACTTTTTATGTTATTTTTTCTATTCGTTTTGGTACCAGTCGCCTTCGGGTTTCAATAA
ACGGAATTGTACACTAAGAAAA

>27 c9752_g1
GATAGCTTCACCTGTTCATAACATGACCGCACCTGCAACAATACCGCACATCCGTACCTCTAAAAACAAAAAC
AGCGTACACCGTCCGGTAGAAATAGGGCTACTACCAAATAGAGAAAGCCCAAGATGCTCAAGGGCTCTGAC
AGGAAATGGCGAACATGGATGTCGTTCCAGTCTGCTAGCTACTACTGGAAATTTCCACACATCCGACAAATTT
TCCAGACTGATTGCATTAATGCTTAAGTTAAATTTTTTACGTAAATGGTCAAATTTATATTCAATGGAAATGTTG
CGGTATTGCTGCAATTGTATTGTCTGAAAGCAAGAGGGCAAGCCTTAATGCATGCACAAACAAAGCGAAAG
GACTTGGTAATAGGAATTTCGGATTTGTATATAATAAAGGAGTGGTGGGGCGAAGAGCAGATCGATAGCTTC
AGTTGGAAAATAAATAAAGGAGTATGAGGAAAAGGGCGAAGAAATGTAACAGTTAACAATAGCTTTCGAGC
AATTTAAGCATTGTCAGCTTCGGCATCGGGATCTTCCTCAAAATCCGCCTCATCATCAGCCGTAGCTTCTTGAT
ATTGTTGGTACTCAGAAACGAGATCGTTCATGTTGGATTCAGCTTCAGTGAACTCCATTTCATCCATGCCTTC
ACCAGTGTACCAGTGGAGGAAAGCCTTACGACGGAACATGGCCGAAAATTGCTCCGAAATGCGTTTGAAG
AGTTCTTGAATAGCTGTCGAGTTGCCAATGAAAGTCGCCGCCATTTTCAAACCACGGGGAGGAATATCGCA
GACGGCTGTCTTGACATTGTTGGGAATCCATTCGACAAAATAGGAAGAATTTTTGTTCTGAATGTTCAGCATC
TGCTCGTCAACTTCCTTCATGGACATGCGGCCACGGAAGATGGAAGCTACGGTTAGATAACGGCCATGACG
AGGATCACAAGCGGCCATCATGTTTTTGGCATCAAACATTTGCTGAGTGAGCTCTGGAACAGTAAGGGCGC
GGTATTGTTGTGAGCCTCGAGAAGTCAGCGGAGCGAAACCAGGCATGAAAAAGTGCAGACGAGGGAATG
GGACCATGTTGACGGCCAATTTTCGAAGATCGGCATTGAGTTGGCCTGGGAAACGGAGGCAAGTTGTCAC
GCCCGACATGGTAAGCGAGACAAGGTGGTTGAGATCGCCATAAGTGGGCGTGGTTAGTTTGAGCGTGCGG
AAGCAGATATCATACAGGGCCTCGTTGTCAATGCAATACGTTTCATCTGTGTTCTCAACTAGCTGGTGGACAG
AAAGAGTTGCGTTGTAAGGTTCAACAACCGTGTCAGAAACCTTGGGAGACGGAACCACGGAGAACGTGTT
CATGATACGATCGGGGTATTCTTCACGAATTTTGGAGATCAAGAGGGTTCCCATGCCAGATCCAGTACCACCT
CCCAAGGAGTGGGTTAGCTGAAATCCCTGAAGGCAGTCACATCCTTCTGCTTCCTTCCTGACAACATCAAGA
ACAGAGTCAACCAATTCAGCCCCTTCAGTATAATGGCCCTTAGCCCAGTTGTTGCCAGCACCAGATTGACCA
AACACGAAGTTGTCAGGCCTGAAGATTTGGCCATAAGGACTAGATCGAACAGAGTCCATGGTACCTGGCTC
CAAATCAACAAGAACTGCTCTTGGAACATATTTCCCACCACTGGCCTCATTGTAGTAGACATCAATCCTCTCTA

GCTGCAGTTCAGACTCGCCTTGGTATACACCATTGGGCTGAATACCATGTTCATCAGAGATGATCTCCCAGAA
CTTGGCACCGATTTGATTTCCGCACTGGCCGACTTGGATGTGGACGATCTCACGCATTTTGGCAAGTTTCTTT
AAGAGGAATGGGTGGGACTGCAGCGAACAGTGCGTACTTGGGTAGTACTCGCTTTAACTTTCGTCGTCGAT
ATCGTGAGTAGCATGACATGGGGTGGTATATATACCTTCCGATCTGGCAGTGGTAGCCGTAGGCTAGCTAGCG
AAACTGTAGTGCAACGAAATCTGGCAAGCTCGCCACATTCAATTGACACGCCTATTTGAAATTATTTTCGAAA
TTTAATGATAATTTCTCAAATAATTTTTTTTCAGGTTCCATTCCATTTGTTCTCGAAGCAGTGTGCCGTTTTTTG
CTATCGATATTCTGTATCGATAAAAAATTGATACAATAGTATTTTTCTGAACATCTGCAATATTTACTGTAAAATC
TCACATGCAAAAAGGTACGTACAATTATCAAATATTGTTCATTTGAAAGTAGTATTCTCCTCTTTTTGAGTTTAT
GGCATCATGACTTTTCTGTATGCTCGATTGCCCTGATTCCTTTTAAAGAAGATTTCTAACATCCCTTAATTTTTT
GCCTAAAAGCCTTTCATTTAATGACGAAGAAAAACAGACAAGCGCAATGCAGGTTTGTGGTATGAGAAACA
TGCAATGAAACTCTAGTCACGCCATCACAGTACACAGGGGCTATTGTCTTTCAAACAGTATGCCATCAATCAC
GTACTCCTCGTCAAACTCATGGCACTATCGGAGAAATTTCGACAGGGAAAAACTTGTGCGAAAACGGTCATC
CTACGTCATCGAATTTTTATGGTGCTGTGCCTTGTGCAAAGACCATTGAAGGTCACATTTCTTAAGACTTATTT
GGAAGTTACGATTGAAAGTTGAAACGAAAAATGGAAGAAACAAGAAGGAAGAATCTCCTTTCACGCCCAC
TAAGACCATCTAACGGCGGGAATATTAAACTACGCCAATATTTATGTATCTTAGAAGATAGCACTGGATATAAG
TTTCTTTTTATTGCAACAAGTAAAATTTCTTTTAACATTTACAGCTACTGTTTTTATTTCCTCGAGATCGTGAGC
CCTCAGTCACAATGCAAGATCGTGATAAATTATTTTGGCGTACTAATCGCTTGGTAATGTTTTTGCTATTTCCG
AGATTTTCTCCATATTCCCATTTTCTGAGTGATTGTTGTGGATGCAAAAAAGCATTGAAATTCTATGCTGTCTG
GGTGCATTACTCGAGTATGCTTCTATAAAAAAGCTTGAACTCCAATTTAAAAAAATTAGTTTTTAATTCCCAAT
CTGATTTACTTTTTTATATTGTCAGAACAATCATCAGTGGTTGCTAATGAAAAGTAAATCGAGAATCCCTACTC
CCTTTCCTTCCCCCTTCCTTCTAAAAAAAGAAAACCAAACAAAAAATTTTTGGTACAAAAACTGTCAGACCA
GTGCTTTTTTAAAATATGAAATAGAATAACTTGTAAGAGATTTTGTAAAGGAAATATTTCCGTTTAATAATTTAC
ATTTTTTTTTTTTTGCTGTAATTTTAAAGTTTGGTTTACGATTTCCAACACCAGGTGCATCACCATCTGCCTGTT
TGTCGAACTGTTGATCGTTTGGAAGCTTGTAACGTAGTTTGCCAAGTGTTA

>28 c15362_g1
TTCAACTTGGCGAGGTTGTGTCCGGTCAGACGTATATAAAGGCCGCCGCTTTGCTTCAATAGAATCAAACAA
ACTCACAACAACAGACTAAAGCAGTTCTGTGTTCAAAGCATCTCGTGTATAACGCTCGTTTCAGTCCAGAAG
AACATTTTCTTTTAGAGGAAAATGGCAACTAACAACATTGTAAAGAACGTCATTGCTAAGCGCTTGATGTCG
ACATCCGGCCAAACTACTAGATACATTTCTCCGGTCTCAGCCCTAAGTCAAATTAGTCATAAGGTCAGCCCGT
TCGAGCAGCGATTGCTCGTGTGGAGCGGGCAATTCAAAAAGATTACCGATGTTCCCTCTCTTGTCTCACGCG
ATACAATGGAAAAGGCACGCAATCATTACCGGATCCGCGTTAATCTCATCATCGCCGGTCTGACGGTTCTAGG
CTCGTTCGCCATGGTAATCAGCGGAAAGAACGCAGCCCATCGCGGGGAGAACATCAACGACTACAACGCCG
AATGGCATCGAAAATACAACGAGGCCCACAAATACGATCATTAAGTGATAATAGAATTATTATGATTTCGACA
GTCCGCATGAAAAAAAACGACGCAAAGAAATGAAGTGAAATGTGGAAAAGTCAGGATGTGAACAAATTGA
CTGTTAGATGTCGTGGCTTGTGATAATGTGACCCTTTTCACTTAGATTTCAGCTTTAAAAATAATTTTTCTTTTT
GCTTTCTTGCCCTTTCCAAACGTTATGTGATCATCGTAACTGGTATTAGTAGACTTGTGAGTCCTCTTTTTTTTT
CAATAGGTAATTTTTCTTTTGAAATCGACTTGTCAAAAAACAATCGTGGCATTCCTTGTTATGTGTTATTCGAA
AATAAAAATACAGTTGAAAGTTCAAAACAATATGTGTCAAAAG

>29 c13754_g1
TTTCTATTTGCTAAACCATTTATTGAATATTACCAGAATTTCAGAGAATTGCAACACTGCTTCAGTGTTCGACG
CGGGAAAAAGTAAACCTCGTAGAGGCAACAAAATCCCTATAATTTTAAACAACACCTTTTGAAATGTCGACA
AAAAGCCAGCAGGGTAAACCGTGGAAAATTAAAAGATGATTTAATTGGGAGGGGGGTGTCCTGTAAATTTT

AAATAAAAGTAACCAATAAAAAAATTAACTTTTGCTCCGCGATGTTGAACGCATTAGGCTTCATATTCATTTTT
TGTTTGCTGGATTTCTTTTGTGATCTAGAAGCTAAAAACGTCAACTTCATCGCGTCCTCTTGACATTATTTCGG
AGTCGGGATTGAACGGGCTAAAATCATTACGGGGTTTCAACAGTTTTAGACATTTGCTGCTATCCAGTCCAA
GTATGAACCAACCCTCCCGTAAATGCCTGGATATTCCGTTACGCCGCATTCTTCACCGTAGCTGACGACGCCA
ACTTGTACGAAACGGCATTGTGACACAGATTCACCATAATCTGCCACAAGAGGGCCGCCACTGTCACCCTGA
CACGTGTCTTTTTCACCTCCATAAGCGCAGAGCATATTGTCGAGAATTTGATCTTTCTCTCCGTAGGTTTCTTG
GCACTCTTTGTTGCTCACCACTGGAAGGACGGCGTGTCGTAGATATTCGGAAGTTTCGCCTTTGTATTTGATT
GTTCCCCACCCCATGCCAATCGTTTCCATTCCGTCAGTGGGCAAACACGACGGTGCCAAGCAAATGGGTTGA
ATAGTGTCCGTATATTCCACAGGCGATTCCATCGTCAAAATAGCAAAATCGTTCTCAACTGTCTGGGCGTTGTA
TTCTTCGTGAATTTTCATGTTTACGACCTTTCGTTTTTCCTGGGAGTCGCTCGATGTTTTGGTAATCTTGTGCA
TTCCGAGTCTTACATGGATGTCATCCGTTATTAAAGTTTCAGTTTTCTCTTTGGTAACGCAGTGGGCAGCCGT
TAGGATTTTGGTCGGAGTAATCAAAGACCCGCCGCAAAAAATTTGAAGGTCTTCACTTTCCTCATCACCCATA
GCGAGCGCCACCACGAATGGATATTGATTCGGAGTGGCTTCTTCACTCTCCGTCATTTTAAACGTGCTATTTG
ACCTAGCCGTTCGAGGTAAGGAGCGAAGATTGTTGCTTTCACAGAGAGAATCTTCGGCAATGGATGGTTGA
TCACCATCGTTCTTGATAATGGCAGCGGCGTACGCAACTGGACTGGCAACAGGAGCGTCAGGGGCAGGGG
CATCTGGGACAAACGCGTACGCCACCGGAGTGTCAGCAGGAGCGGCAAGGACAGGAACATCTTCGACGG
GCATGTACGCTACCGGGGTATCGACAGGAGCGGCAAGGACAGGAACATCTTCGACGGGCATGTACGCTACC
GGGGTATCGACAGGAGCGGCAAGGACAGGAACATCTTCGACGGGCATGTACGCTACCGGGGTATCGA

>30 c16548_g1
GGGTCTTTTCAACAACTGTGGTTTTCGGACTGACAGATCTTTATTCCTCCCTCACCGAGAATACACGCGATAC
TTTCGTTAATTAACAACAATAATAAACTTATTTTTTGTGATTGGGTTTGCGATAAGTCGTCATCAAATATTTTAA
CTTAGAGAGCACAATTTGAGGTGTCAGTGTTCTGTGGCCAGTCACAAACAGTTTTGGCTGCATTCCACACG
GATTCACCCGGGCAATCCAACAATCGGGGAGATCCCTGAACGCACCAGTAGTACTTGCTGCATTTTTCGTGA
GGCCAGTATGTCTGGACGTTGCAGTCTATTCCGGCGACATCAACTGGCTTAACTACAACGTCTGGATTGCGT
GTTGTTGTTTGTTCTGCTGGTTTACTTGTTGTGTGGGCGCTCATCGACACAGTAGGGTCTGGTTTCCAAGGT
TTCCACCATGTTACTGCGGTTGTGGTTGTCATAGGAGGTGGGATTGGAACAATATAGTCTTTCATTCCGTCGT
AGATGACCTTCATCATGGAATGATGGCCCTTCCCGCACAAGTTGTGGAAGTCATCCATATCAATGGCCCAAGT
CATTGCTCCACCATATCCCCGTGCTCGAATAAAATCCATTTTAATTTTCAGGGAGTCCTCGTCTTCGTATCCGAT
CCACTGGCGATCTTTGTGTGTGAATGGAACCTTGCCGACGTCGTCGTATCGCTTAACCCAGCCGTCAGCTGG
ATCCAGAAGCTTCATGCAAATTTCGTAGTGCGCCATAAAGCCGGATGCGTTTGTATAAGGACCAGGTTCTCC
ACCCCCGGCCCATTTGACGATATATGCTCCCAAGTCATTGCTTTTGGGATCACCTAGAGTGTAAGTACGTCCG
TAAAAGGGTGTACCTACGACAAGCTTATCTCTTGGGCAACCAAATTCTTCCCACAGCAATAAGCCATCGTTCA
CGTTGAGTTTTTCATAGGCGTACTGATCCAGACCTGGGCGACGATAGAGTTGCGAATGAACGTCAGCAAATC
CTACCCAGTTTCCTCGCAGATCGTATGTCATGACGTGGATAGCATCGAGCAATTGGCATAATTCAGGGACATG
ATATCCTTCTTGCAAGCGGAATTTAGCAACAGGAACCGCAGCTGTTAGTTCCCATCCTTTTCCGACAGTGTCG
AATGCAGCGCGCAATTCCCGAACAAGAGCAAGGAAGGTTTCCTTGTCGGCATAGGTGCCTCCGCGATCTGC
AGCGCCCGGGTATTCCCAATCAAGATCAAACCCGTCAAAACCATATTGGTTCATATATTCTACGACACTGCTAA
CGAATGCTTGACGACGCGCCGGCACGGAAGCCATTTGCGAATACTTTTTGCCACCTTCACCCCATCCCCCGA
CAGCCAGCATGGGTTTCAAATGCGGATACTTCTCTTTCAAAGCTACAAATTTAGCGTAACCTCCTTGTTCTATG
TCTCTCTCTGGATCGAGAACAAGAACGCCCCAGGTGACGTTGCTGACACCACAGAAAGAATAAATTACGTG
CGTACACATTTCACCGGGGATGTCGTCGATGTCGTACGAACCCTTACCTGGGCGGTAGACTGCCCAGTTGCT
GAAGTAGCATACACGCTTAGCTAGGCGATCGGGCAAGGCATTTCGTGAATTTTGGCTTTCAATGACGTCCGA
TGAACGAGGTTGGGCAGCGAGCGCAACTTGTAGAGTAAGTGCGCATATGAAAATCAAGGGCTGCCACATTT

TAATCAGCCCTGTCTATATTTAACTTAAGTAACACTAACACACGTCTCAACCTGCTGCGTCCTCACGATACGAC
TGTCGAGCAAGC

Table S2. Primers used for qPCR.

Gene ID	Forward (5'-3')	Reverse (5'-3')
c22292_g2	GCCGATGAGCTACGATAA	GCCTTTCTTGTCTTGATTGT
c36850_g1	CGGTATCTACATCATCAG	GAATCGTAGTTCGTTGAG
c22292_g1	TGCTTACTCATCGGACTC	GTACAAGAATTGGTCGTCAG
c14229_g1	GGATACTCTAATGGCGGATA	AGAAGTGGTCGTTGGTAG
c11892_g1	AGTAGTTGCCGATGTTCA	TCTTGGACCTCTTCTTGTT
c21753_g2	GAGAAGGAATTGGATGAGATG	GAAGGCAGCGATAACAAC
c8533_g1	GGTTGACGAACTGGAGATA	AGTTGGCTGACCATAATAGT
c22798_g1	GCGTTATCGTCCAGAGTA	GTCCTCCTCACCTTCAAT
c23345_g1	GCCTACTCCTACGATGAC	AAGCCTCAGACTCAACAT
c23476_g2	ACTACTGGTTCATCTCTTCC	CTTATCGTTAGCCTTGACTTC
c18434_g2	CAAGAAGCGTCAGGTTATC	AGAGTAAGCATAGCCAGAAT
c16911_g1	CTCGGTCGTATCTGTCAT	GGATGTCCATCTTGAGTCT
c16323_g1	ATTCCAACTTGCTCATTCC	GTAAGGTTCCACAACAGTATC
c22501_g1	GTGAAGATGAAGAGATGGAAG	TGGAGGCTGCTTAGATTC
GAPDH	AGGAAGCCAGTTATGATGA	AAGTCTTGTTGAGAGCAATG
ACT	CCATCCACCATGAAGATTAAG	CTCGTCGTACTCTTGCTT
